# No evidence for sylvatic cycles of chikungunya, dengue and Zika viruses in African green monkeys (*Chlorocebus aethiops sabaeus*) on St. Kitts, West Indies

**DOI:** 10.1186/s13071-020-04419-1

**Published:** 2020-10-30

**Authors:** Matthew John Valentine, Brenda Ciraola, Matthew Thomas Aliota, Michel Vandenplas, Silvia Marchi, Bernard Tenebray, Isabelle Leparc-Goffart, Christa Ann Gallagher, Amy Beierschmitt, Tatiana Corey, Kerry McAuliffe Dore, Xavier de Lamballerie, Chengming Wang, Courtney Cuin Murdock, Patrick John Kelly

**Affiliations:** 1 One Health Centre for Zoonoses and Tropical Veterinary Medicine, Ross University School of Veterinary Medicine, West Farm, Basseterre, St. Kitts and Nevis; 2 University of Minnesota, Twin Cities, St. Paul, MN USA; 3 Department of Biomedical Sciences, Ross University School of Veterinary Medicine, West Farm, Basseterre, St. Kitts and Nevis; 4 National Reference Laboratory for Arboviruses, Institut de Recherche Biomédicale des Armées, Marseille, France; 5 Unité des Virus Emergents (UVE), Aix Marseille Université, IRD 190, INSERM 1207, IHU Méditerranée Infection, Marseille, France; 6 Center for Conservation Medicine and Ecosystem Health, Ross University School of Veterinary Medicine, West Farm, Basseterre, St. Kitts and Nevis; 7 Behavioral Science Foundation, Estridge Estate, Basseterre, St. Kitts and Nevis; 8 St. Kitts Biomedical Research Foundation, Bourryeau Estate, Christ Church Nichola Town, St. Kitts and Nevis; 9 Virscio, Inc, New Haven, CT USA; 10 Department of Anthropology, Baylor University, One Bear Place, Waco, TX USA; 11 Department of Pathobiology, College of Veterinary Medicine, Auburn University, Auburn, AL USA; 12 Department of Infectious Diseases, College of Veterinary Medicine, University of Georgia, Athens, GA USA; 13 Odum School of Ecology, University of Georgia, Athens, GA USA; 14 Department of Entomology, College of Agriculture and Life Sciences, Cornell University, Ithaca, NY USA; 15 Center for Tropical Emerging and Global Diseases, University of Georgia, Athens, GA USA; 16 Center for Ecology of Infectious Diseases, Odum School of Ecology, University of Georgia, Athens, GA USA; 17 Center for Vaccines and Immunology, College of Veterinary Medicine, University of Georgia, Athens, GA USA; 18 Department of Clinical Sciences, Ross University School of Veterinary Medicine, West Farm, Basseterre, St. Kitts and Nevis

**Keywords:** Sylvatic cycles, Dengue, Chikungunya, Zika, Arboviruses, Non-human primates, Mosquitoes, Arboviruses, Blood-meal analysis

## Abstract

**Background:**

Dengue, chikungunya and Zika viruses (DENV, CHIKV and ZIKV) are transmitted in sylvatic transmission cycles between non-human primates and forest (sylvan) mosquitoes in Africa and Asia. It remains unclear if sylvatic cycles exist or could establish themselves elsewhere and contribute to the epidemiology of these diseases. The Caribbean island of St. Kitts has a large African green monkey (AGM) (*Chlorocebus aethiops sabaeus*) population and is therefore ideally suited to investigate sylvatic cycles.

**Methods:**

We tested 858 AGM sera by ELISA and PRNT for virus-specific antibodies and collected and identified 9704 potential arbovirus vector mosquitoes. Mosquitoes were homogenized in 513 pools for testing by viral isolation in cell culture and by multiplex RT-qPCR after RNA extraction to detect the presence of DENV, CHIKV and ZIKVs. DNA was extracted from 122 visibly blood-fed individual mosquitoes and a polymorphic region of the hydroxymethylbilane synthase gene (HMBS) was amplified by PCR to determine if mosquitoes had fed on AGMs or humans.

**Results:**

All of the AGMs were negative for DENV, CHIKV or ZIKV antibodies. However, one AGM did have evidence of an undifferentiated *Flavivirus* infection. Similarly, DENV, CHIKV and ZIKV were not detected in any of the mosquito pools by PCR or culture. AGMs were not the source of any of the mosquito blood meals.

**Conclusion:**

Sylvatic cycles involving AGMs and DENV, CHIKV and ZIKV do not currently exist on St. Kitts.
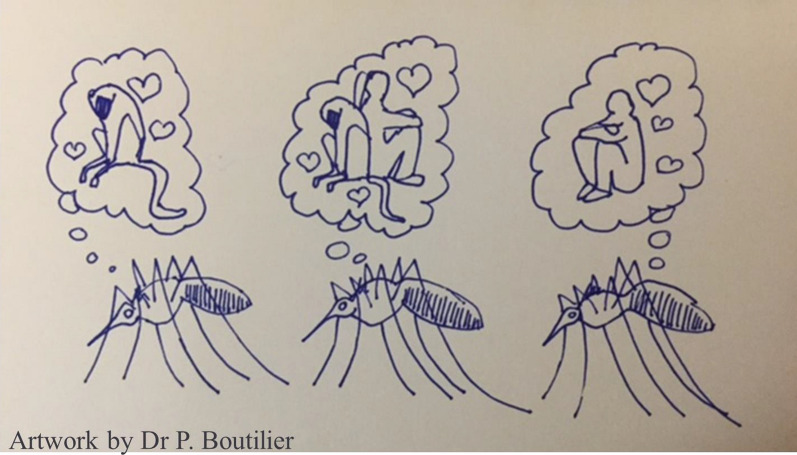

## Background

Chikungunya, dengue and Zika are arboviral diseases that are transmitted by the anthrophilic mosquitoes, *Aedes aegypti* and/or *Aedes albopictus* in an urban transmission cycle resulting in epidemics and pandemics in tropical and subtropical regions of the world [1–3]. The aetiological chikungunya (CHIKV), dengue (DENV) and Zika viruses (ZIKV) evolved in non-human primates (NHPs) and sylvatic mosquitoes in the forests of Africa in the case of CHIKV and ZIKV, and Asia in the case of DENV [4–6]. In the forests, primatophilic forest mosquitoes maintain the viruses in sylvatic (NHP-mosquito-NHP) transmission cycles which continue to this day [3, 7–9]. It remains an outstanding question whether sylvatic cycles of these arboviruses are present elsewhere in the world where there are similar non-human primate vertebrate hosts and mosquitoes [8, 10–12]. Furthermore, some researchers have identified tropical islands as ‘hotspots’ for arboviral emergence [13].

On the Caribbean island of St. Kitts there is a large population of wild and captive African green monkeys (AGMs) (*Chlorocebus aethiops sabeus*). People on the island were affected by the chikungunya pandemic in 2014 and the Zika pandemic in 2016 [14, 15]. Dengue is hyperendemic in the region and there are periodic outbreaks on St. Kitts, most recently in 2008 [16]. While there are no accurate seroprevalence data on arboviral infections of people on St. Kitts, studies from nearby islands show very high CHIKV exposure rates of 16.9% [17] on St. Maarten and 25% in Puerto Rico [18]. Similar exposure rates have been suggested for ZIKV [19]. The confirmed presence of arboviral disease in people, a suitable vertebrate host (AGMs) and a diverse mosquito community [20] suggests there is potential for sylvatic transmission on St. Kitts.

To investigate this possibility, we used previously established techniques [7, 21–24] involving serology to determine exposure of the island’s AGMs to arboviruses, and RT-qPCR and viral isolation in cell culture to determine the presence of arboviruses in potential vector mosquito genera. Additionally, DNA extracted from blood meals of engorged female mosquitoes was analyzed by PCR and sequenced to determine which mosquitoes had fed on AGMs and/or people.

## Methods

### Study area

St. Kitts is a 168 km^2^, geographically isolated, volcanic, Caribbean island located in the Lesser Antilles (17.33°N, 62.75°W). It has a population of approximately 40,000 people mostly inhabiting urban Basseterre, the capital, and a string of small village communities distributed along the main coastal road that circles the island. Rainforest covers the uninhabited, steep volcanic slopes in the center of the island, surrounded by lower gentler slopes consisting mostly of abandoned sugar cane fields or arable farmlands. The south east of the island is primarily an arid peninsula covered mainly in scrub with beaches, mangroves, and salt-ponds. AGMs were introduced to the island in the 1700s during the slave trade and are abundant with an estimated population of 55,000 [25–27]. Due to changes in land use and their adaptable and opportunistic nature they are now widely distributed, commonly encountered, a tourist attraction and also a problem for local farmers whose crops suffer from their destructive behaviour [25]. They are semi-arboreal, roosting in trees at night, but highly mobile on the ground during the day when they forage through many of the ecosystems on the island [28]. While foraging for food and water, which can be over large areas depending on availability that varies with season, it would be anticipated that AGMs should come into contact with, and potentially host a wide variety of mosquitoes although we know of no data on the species involved.

### Determining the infection status of the AGMs

**Fig. 1:**
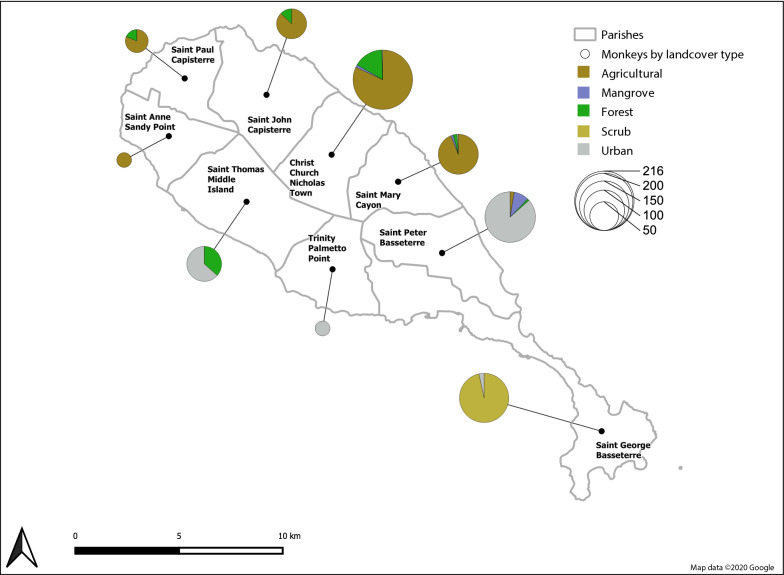
Approximate capture locations by parish and land cover of AGMs on St. Kitts. Numbers of AGMs captured in each land cover indicated by a coloured pie chart. *Key*: brown, agricultural; light blue, mangrove; light green, rainforest; dark yellow, scrub; light grey, urban. Parish boundaries are indicated by a grey line

Between January 2013 and March 2019, we obtained 851 convenience samples of sera from AGMs undergoing routine health screening after being trapped using accepted procedures [26, 27] for two primate research facilities on St. Kitts. We also obtained seven sera from AGMs trapped and immediately released by professional monkey trappers for our study (Additional file 1: Text S1). Monkeys were captured across five different land covers that we identified on the island: agricultural; mangrove; urban; rainforest; and scrub (Fig. 1 and Table 1) [20].

**Table 1:** Capture locations of AGMs on St. Kitts by parish and land cover

St. Kitts Parish	Agricultural	Mangrove	Rainforest	Scrub	Urban	Total
Christ Church Nichola Town	177	3	35	0	1	216
Saint George Basseterre	0	0	0	143	5	148
Saint John Capisterre	48	0	7	0	0	55
Saint Mary Cayon	92	2	3	0	1	98
Saint Paul Capisterre	26	0	6	0	0	32
Saint Peter Basseterre	4	15	2	0	135	156
Saint Thomas Middle Island	0	0	27	0	47	74
Trinity Palmetto Point	0	0	0	0	2	2
Saint Anne Sandy Point	1	0	0	0	0	1
Total	348	20	80	143	191	782

Sera from 268 of the AGMs captured prior to the Zika outbreak in 2016 were screened for IgG to DENV and CHIKV with commercial ELISA kits (Panbio Dengue IgG Indirect Elisa. Standard Diagnostics Inc., Yongin-si, Republic of Korea; Anti-Chikungunya virus ELISA (IgG). Euroimmune AG, Lübeck, Germany) following the manufacturer’s instructions. They were also tested for IgG to DENV and CHIKV at the National Reference Centre for Arboviruses in Marseille, France (IL-G) with an in-house ELISA for with peroxidase labelled anti-monkey IgG (KPL, Gaithersburg, MD, USA) as a secondary antibody. The remaining 590 sera were also screened for antibodies to DENV and CHIKV with the commercial ELISA kits described above and additionally for ZIKV IgG antibodies (Monkey Zika Virus IgG (ZV-IgG) ELISA Kit, MyBioSource, Inc., San Diego, CA, USA). Sera with positive or equivocal ELISA results were repeated and then tested by Plaque Reduction Neutralization Test (PRNT) [29–31] on Vero cells (ATCC #CCL-81) with a cutoff value of 90% (PRNT_90_). Neutralization curves were generated using GraphPad Prism software, and the resulting data were analyzed by nonlinear regression to estimate the dilution of serum required to inhibit 90% of infection. We considered an animal to have a confirmed ZIKV exposure if ZIKV PRNT_90_ was at least 20 and a ratio of ZIKV PRNT_90_ to DENV PRNT_90_ titre of at least 4.

### Determining the infection status of potential arboviral vector mosquito species

#### Mosquito collections

From September 2017 to March 2019 we conducted a comprehensive mosquito survey [20] and captured mosquitoes each month for a 48-h period (excluding December 2017, April 2018, and December 2018) in all five land covers described above using carbon dioxide (CO_2_) baited CDC light traps (J.W. Hock, Gainesville, FL, USA) and/or Biogents Sentinel 2 traps (BGS) (Biogents AG, Regensburg, Germany) to capture a broad range of mosquito species and target host-seeking mosquitoes which are most likely to have fed on mammals [20]. Since it was not possible for us to trap mosquitoes in the often inaccessible areas where AGMs sleep and forage, which both vary considerably, we used sites described in the mosquito survey [20] and these were set within 100–200 m of known AGM trapping locations where possible. Trapped mosquitoes were transported to the research laboratory of Ross University School of Veterinary Medicine (RUSVM), Basseterre, St. Kitts, and stored at − 80 °C for later identification. Potential arbovirus vector mosquito species (*Aedes aegypti*, *Aedes taeniorhynchus*, *Culex quinquefasciatus* and unidentified *Aedes* and *Culex* spp.) were identified using standard morphological keys [32–34]. For arboviral testing (below), 1–50 individuals of each species were pooled according to location where they were trapped, month, and sex.

#### Mosquito processing

Mosquito pools were homogenized in a 2 ml microcentrifuge tube containing 3–4, sterile, steel ball bearings (4 mm in diameter) with 600 µl of minimum essential media (MEM, Gibco, Waltham, MA, USA) with 1% penicillin and streptomycin (Penicillin-Streptomycin, 10,000 U/ml, Gibco Waltham, MA, USA) and agitated for 5 min using a vortex. Homogenates were clarified by centrifugation (5 min at 14,000× *rpm*) at 4 °C and the supernatant, approximately 300–500 µl, filtered (0.22 µm syringe filter Millipore Millex™ Sterile Syringe Filters. Merck KGaA, Darmstadt, Germany) and stored at − 80 °C.

#### RNA extraction and RT-qPCR

After thawing at room temperature, RNA was extracted from 100 µl of the mosquito lysate using the RNEasy Mini Kit (Qiagen, Hilden, Germany) and analyzed by RT-qPCR using the ZDC (Zika, Dengue and Chikungunya) Multiplex RT-PCR assay (Bio-Rad, Hercules, CA, USA) according to the manufacturer’s instructions on an ABI 7500 Fast Dx Real-Time PCR system (Applied Biosystems, Hercules, CA, USA).

#### Cell culture for viral isolation

Mosquito lysates (100 µl) were inoculated into 24 well cell culture plates seeded with approximately 5 × 10^4^ Vero cells in 0.5 ml of MEM with 1% Fetal Bovine Serum (Gibco), 1% Penicillin-Streptomycin (Gibco), 1% glutamine (Gibco) and 0.1 % Amphotericin B (Gibco). Following gentle agitation on a rocker for 1 h to allow virus adsorption, 1ml of MEM (as above) was added to each well and the plates incubated at 37 °C for 7–10 days. Every day the cells in each well were monitored for cytopathic effects with an inverted microscope (CKX53, Olympus, Tokyo, Japan).

### Blood-meal analysis

Engorged blood-fed female mosquitoes (*n* = 122) were retained individually and their abdomens aseptically separated from the head and thorax by sharp dissection. Their DNA was extracted using a DNEasy Blood Mini Kit (Qiagen, Hilden, Germany) and used in a qPCR with primers designed to anneal to the hydroxymethylbilane synthase (HMBS) gene as described by Wei et al. [35]. DNA extracted from the whole blood of five AGMs were used as positive controls and their sequences (ELIM BIOPHARM, Hayward, CA, USA) aligned with Clustal Omega [36] to obtain a 222 nucleotide sequence for the HMBS gene of *Chlorocebus aethiops sabeus* (Additional file 2: Text S2) (BankIt2363830 AGM_seq MT742560). Amplicons obtained from the blood-fed mosquitoes were also sequenced and raw sequence data was compared with the AGM HMBS (BankIt2363830 AGM_seq MT742560) and human HMBS sequences NG_008093 on GenBank using Clustal Omega.

## Results

### Serology of AGMs

The 268 sera tested with an in-house ELISA in France and with commercial ELISA test kits for antibodies to DENV and CHIKV all gave negative results. The remaining 590 samples tested with the commercial kits for antibodies to DENV, CHIKV and ZIKV were all negative except for 10 (1.7%) that were positive for ZIKV IgG. On subsequent confirmatory testing by PRNT all ten tested negative for ZIKV and DENV neutralizing antibodies using PRNT_90._

### Arbovirus detection

We captured 9704 individual mosquitoes representing 10 of the 14 mosquito species across all 6 genera previously documented on the island [20, 37] (Additional file 3: Table S1). Approximately half (3000–4000 individuals, 190 pools) of the mosquitoes from non-urban land covers were trapped within 100–200 m of where AGMs were blood-sampled for serology. All the 513 pools of mosquitoes (Tables 2, 3) tested by RT-qPCR for CHIKV, DENV and ZIKV were negative. Furthermore, all virus isolation attempts in Vero cell cultures were negative.

**Table 2:** Land cover of mosquito pools tested for DENV, CHIKV and ZIKV by multiplex RT-qPCR and numbers of individual blood-fed mosquitoes tested by qPCR for mammalian DNA (blood-meal analysis) from each land cover between September 2017 and March 2019

Land cover	Pools of mosquitoes tested for DENV, CHIKV and ZIKV	Individual blood-fed mosquitoes tested in blood-meal analysis
Agricultural	58	11
Mangrove	236	42
Rainforest	15	4
Scrub	77	20
Urban	124	42
Unidentified	3	3
Total	513	122

*Abbreviations*: CHIKV, chikungunya virus; DENV, dengue virus; qPCR, quantitative polymerase chain reaction; RT-qPCR, reverse transcriptase quantitative polymerase chain reaction; ZIKV, Zika virus

**Table 3:** Pooled mosquito species tested for DENV, CHIKV and ZIKV by multiplex RT-qPCR and species of individual blood-fed mosquitoes tested by qPCR for mammalian DNA (blood-meal analysis) between September 2017 and March 2019

Mosquito species	Pools of mosquitoes tested for DENV, CHIKV and ZIKV	Individual blood-fed mosquitoes tested in blood-meal analysis
*Aedes* spp.	44	8
*Aedes aegypti*	87	14
*Aedes taeniorhynchus*	153	12
*Culex* spp.	106	41
*Culex quinquefasciatus*	114	37
*Psorophora pygmaea*	NT	1
Unidentified	9	9
Total	513	122

*Abbreviations*: CHIKV, chikungunya virus; DENV, dengue virus; NT, not tested; qPCR, quantitative polymerase chain reaction; RT-qPCR, reverse transcriptase quantitative polymerase chain reaction; ZIKV, Zika virus

### Blood-meal analysis

Of the 122 blood meals we tested with the HMBS PCR, we obtained useable sequence data from 106 sequences (including 13 from *Ae. aegypti*, the only known vector of DENV, CHIKV and ZIKV recorded on St. Kitts) that aligned in the polymorphic 286 base pair (bp) target region of the AGM consensus sequence and human HMBS reference gene (NG_008093.1) [31] containing 5 reliable single nucleotide polymorphisms (SNP) and 2 deletions (Additional file 2: Text S2). Of these, one blood meal from *Ae. taeniorhynchus* caught in scrub land cover (mosquito 106) had 100% sequence match (227 bp) with the human reference HMBS gene (NG_008093.1) compared with a 95.5% (213 bp) with the AGM HMBS gene sequence we produced (BankIt2363830 AGM_seq MT742560). Similarly, we also considered 3 more blood meals with lower sequence matches (83.25–97.64%) from a *Culex* spp. in the mangrove (mosquito 43) and two unidentifiable urban mosquitoes (mosquitoes 64 and 65) to match best with the human reference gene (Additional file 2: Text S2).

## Discussion

Our data do not provide robust evidence for sylvatic transmission of CHIKV, DENV or ZIKV between AGMs on St. Kitts. By capturing and testing both AGMs and sympatric mosquitoes for evidence of arboviral infections we emulated studies from Africa and Asia [7, 21–24] that have produced reliable (and some of the original) data on the existence of sylvatic transmission cycles of these arboviruses [4]. It is of note, however, that some studies on sylvatic cycles relied solely on antibody detection in NHPs [11, 38–42] to provide evidence of arboviral exposure. In other cases, evidence for these cycles has only been based on the presence of arboviruses in mosquitoes captured from sylvatic habitats [3, 7, 21, 43, 44].

The actual population of AGMs on St. Kitts is unknown, but estimates are up to 55,000 [25, 26]. Calculations show that detecting a 1% disease prevalence in this highest population estimate of 55,000 animals at a 95% confidence level requires a sample size of 332 (assuming 100% test sensitivity and specificity) [45, 46]. Our convenience sample of 858 monkey sera would then appear to have been more than adequate to detect low seroprevalences.

Our inability to detect significant levels of antibodies to CHIKV, DENV, and ZIKV provides strong evidence that the AGMs on St. Kitts are not, or only very infrequently, exposed to these viruses. We contemporaneously sampled AGMs during chikungunya (2014) and Zika (2016) epidemics and it would seem reasonable to assume this would have given us a good opportunity to detect AGM infections if there was spillover.

Although the PRNT remains the gold standard serological test for the diagnosis of infections with the different arboviruses, it is time consuming, requires highly trained staff, live viruses, reliable controls and a BSL-2 laboratory (BSL-3 in the case of CHIKV). The IgG ELISAs we used for screening produce reliable negative results and are frequently used in experimental arboviral vaccine models involving NHPs to prove freedom from exposure [47–50]. They have proved to be reproducible and sensitive [51] in arboviral studies involving various NHPs in Africa, Asia and the Americas [38–42, 52–54]. The ELISAs are well suited for large-scale screening for previous infections in field studies because wild infected NHPs mount a robust antibody response although only viraemic for 1–7 days and show no obvious clinical signs [4]. The IgG antibodies can be detected for years post-infection and the commercial kits for humans are widely available and can be adapted for use in NHPs [4]. Our findings confirm the reliability of ELISA testing in the general screening of a population with all of the randomly selected 268 ELISA negative sera also being negative when tested by the National Reference Centre for Arboviruses in Marseille, France. The problem with ELISAs is a lack of specificity because of cross reactivity of antibodies against closely related viruses, although this can also be an advantage as it increases the kits’ screening potential [55]. Sera from the 10 AGMs that were ZIKV IgG antibody-positive by ELISA were confirmed ZIKV negative by PRNT_90_. This maybe within the limits of the test specificity or because of exposure to another undifferentiated *Flavivirus*. We did not have access to the variety of other flaviviruses that occur in the region for more definitive PRNT testing of this sample. The other flaviviruses that have been described in the Caribbean region and have high potential for cross reactivity include West Nile virus (WNV), and Spondweni virus (SPONV). WNV has been isolated from humans, birds and mosquitoes (suggesting active transmission) in Puerto Rico in 2007 [56], and recently 10.7% of equids on St. Kitts were reported to be seropositive to WNV [57]. SPONV is difficult to distinguish clinically and serologically from ZIKV infection and had only been recorded in Africa until 2016 when it was isolated from *Cx. quinquefasciatus* from Haiti [58]. With respect to alphaviruses, Mayaro virus (MAYV) can serologically cross react with CHIKV and was first discovered in Trinidad in 1954 and more recently antibodies have been detected in NHPs in Panama and French Guiana [4]. Further work is underway in our laboratories to identify other arboviruses such as these that might be circulating on St. Kitts.

Although our trapping methods would have influenced the numbers and diversity of mosquitoes we caught, in testing the mosquitoes our trapping selected for, including mosquitoes from areas where the monkeys we serosurveyed were trapped, we found no evidence for the presence of CHIKV, DENV or ZIKV. We used RT-qPCR and virus isolation in cell culture to improve detection rates as has been done, either singly or in combination, to detect arboviruses in mosquitoes in large studies in Africa [59, 60]. The RT-qPCR is very specific and sensitive, detecting as little as one infected mosquito in a pool of 5000, and enables high throughput rapid results with less reliance on a cold chain [60]. Virus isolation is less sensitive than RT-qPCR, detecting viable virus which requires the presence of a robust cold chain, specialized laboratory facilities, skilled labour and time, at least a week.

Our inability to demonstrate arboviruses in the mosquitoes was not unexpected in light of the lack of seropositivity in the AGMs and, in the case of the urban mosquitoes we tested, the fact that only low numbers of dengue, chikungunya and Zika cases have been reported in people on St. Kitts. Low viral prevalence in mosquitoes makes arboviral detection difficult even during disease outbreaks [60, 61]. The low infection rates in people, although likely to be a gross underestimate [62], suggest low infection rates in mosquitoes, decreasing the chances of arboviral detection in them. It would also mean fewer opportunities for infected urban mosquitoes to feed on peridomestic AGMs (‘spillback’ infection from people to NHPs) and in the process provide a source of virus for a sylvatic cycle as has been similarly investigated in Brazil [63–67].

We also found no infected mosquitoes in the more rural areas and rainforest where AGMs are more frequent. This is consistent with our finding that AGMs from these areas were seronegative and indicates that there is no sylvatic cycle on the island. In Africa and Asia, only sylvatic *Aedes* spp. are thought to play a role in the maintenance of sylvatic cycles of DENV, CHIKV and ZIKV. The only mosquito species we found in non-urban areas that is known to carry these viruses in a sylvatic setting was *Ae. aegypti* (Additional file 4: Table S2). DENV was isolated from these mosquitoes captured in forest galleries of Senegal in 1999 [21]. The *Ae. aegypti* mosquitoes we found on St Kitts (257/443; 58%) showed no morphological differences to those captured from urban environments and most likely represent ‘rewilding’. The rewilding was most likely driven by opportunistic use of more varied non-urban oviposition sites found in the Caribbean such as rock holes, calabashes, tree holes, leaf axils, bamboo joints, papaya stumps, coconut shells, bromeliads, ground pools, coral rock holes, crab holes and conch shells [68, 69]. The presence of *Ae. aegypti* in diverse habitats on St. Kitts suggests alternate non-human vertebrate blood-meal sources are available to them.

Despite high host preference of *Ae. aegypti* for humans, this species of mosquito will bite other available mammals. In Grenada, 28% (9/32) of *Ae. aegypti* blood meals were from non-human mammals (mongooses, domestic dogs and cats) [70] and similarly, on Puerto Rico, up to 21% (42/199) of *Ae. aegypti* blood meals were from domestic dogs [71]. Additionally, *Ae. aegypti* will feed on both NHPs and people where they live in close proximity as is the case on St. Kitts. For example, on the tourist island of Koh Chang in Thailand sampling mosquitoes in urban, forested and periurban areas of the national park revealed that 70% (21/30) of *Ae. aegypti* had fed on both people and NHPs suggesting less selective feeding behaviour in these situations [72].

Our blood-meal analysis showed that only a small percentage (3%) of the blood-fed mosquitoes we trapped had evidence of having fed on humans. We confirmed that the generalist feeder *Ae. taeniorhynchus* (and probably *Culex* spp.) will feed on humans and, interestingly, in Puerto Rico this species has been proven to feed on NHPs [73]. Using the specific HMBS gene sequence we determined for AGMs (BankIt2363830 AGM_seq MT742560) we found no evidence that the mosquitoes we trapped had fed on AGMs. This might be because there is a greater choice of vertebrate hosts (birds, mammals, reptiles and amphibians) in the rainforest and hence no feeding preference for AGMs. It could also be because we trapped mosquitoes only at ground level while sylvatic mosquitoes, more active at night [21, 43], might be more prevalent in the forest canopy where AGMs sleep.

## Conclusions

Overall, we believe we have sufficient evidence to discount the presence of sylvatic cycles of CHIKV, DENV and ZIKV on St. Kitts. Using criteria commonly used in similar studies [4], mainly seropositivity and infected mosquitoes, we found no evidence that AGMs were exposed to the viruses or that mosquitoes on the island were infected.

## Supplementary information

**Additional file 1: Text S1.** AGM capture and phlebotomy.

**Additional file 2: Text S2.** Blood-meal analysis.

**Additional file 3: Table S1.** Counts of mosquito species per month on St Kitts from November 2017 to March 2019 with the wet season highlighted in grey (May-November).

**Additional file 4: Table S2.** Counts of mosquito species caught across the five different land covers from November 2017 to March 2019 on St Kitts.

## Data Availability

The data supporting the conclusions of this article are included within the article and its additional files.

## Abbreviations

AGM: African green monkey
BSF: Behavioral Science Foundation
CARPHA: Caribbean Public Health Agency
CHIKV: Chikungunya virus
CNR: French National Centre for Arboviruses
DENV: Dengue virus
NHP: Non-human primate
PAHO: Pan American Health Organisation
PRNT: Plaque reduction neutralisation test
RUSVM: Ross University School of Veterinary Medicine
ZIKV: Zika virus
