# Supplementary material for: No evidence for sylvatic cycles of chikungunya, dengue and Zika viruses in African green monkeys (Chlorocebus aethiops sabaeus) on St. Kitts, West Indies
Source: Parasit Vectors. 2020 Oct 30;13:540. doi: 10.1186/s13071-020-04419-1 (PMC7598228; doi:10.1186/s13071-020-04419-1)
Supplement: Supplementary file 1 — Additional file 1: Text S1. AGM capture and phlebotomy. [file 13071_2020_4419_MOESM1_ESM.docx]

ADDITIONAL FILE 1: Document S1.

Monkey trapping on St. Kitts for biological sampling will be performed by the following means:

Humane monkey traps, used widely throughout St. Kitts, will be constructed and/or renovated in select areas of St. Kitts by experienced Kittitian monkey trappers, namely Mr. Gilbert Gordon. Mr. Gordon has trapped monkeys for over 30 years and is regularly employed on a freelance basis to capture monkeys by the 2 primate research facilities on the island, the government and Kittitian arable farmers for pest control. The traps are being placed in areas where monkeys frequent, and will be comfortable traveling through and eating at for extended periods of time. For this current project from October of 2016, 5 study areas will be selected. Monkeys from each of these selected areas will be trapped, sedated, and sampled as described below. Up to 1000 monkeys are expected to be sampled within this study.

Traps will be baited with mangoes and sugar cane secured so that animals must eat them inside the traps. Troop dynamics, specifically the dominance hierarchy and number of individuals in each troop, will be observed by the monkey trappers for approximately two to three weeks before trapping from hides placed ~20 feet from the trap. This observation enables the trappers to know when monkeys frequent the trap and in what dominance succession the monkeys feed. [Eating in the trap occurs in 2-3 waves, with the first group being the most dominant and then each subsequent group being less dominant. When they are trapping, they usually trap the least dominant group first, and this allows them to eventually trap the entire group].

After this period of observation, the animals will be trapped when an appropriate number of individuals are present in the trap. Traps are large, wire mesh with an entry door at one end, opened by a string leading up to a hide (covered on all sides to conceal the trapper within), and a funnel (made of the same wire mesh as the rest of the trap) on the other end of the trap. Trapping occurs by manually closing the door when the animals are in the trap. The monkeys naturally go to the opposite end of the trap when the trapper stands by the door, and they move (on their own) into the funnel where they can be easily and humanely sedated with ketamine 10 mg/kg intramuscular hindleg injection administered by hand held syringe. Blood samples will then be obtained by femoral phlebotomy.

Administration of sedative drugs and subsequent monitoring will be performed by a veterinarian. Blood sampling (venipuncture) will be supervised by a veterinarian with experience of handling, sedating and venipuncture in this species. Capture, handling, restraint, sedation and venipuncture will follow the Behavioral Science Foundation (BSF) standard operating procedures, an accredited primate research facility, that has decades of experience of working with this species. Any BSF staff involved are familiar and experienced with these procedures. Ross University School of Veterinary Medicine (RUSVM) students involved in the sampling procedure will be trained and directly supervised by the veterinarian following BSF standard operating procedures.

Following the sampling, the animals will be kept contained inside the trap in the shade until all animals are completely recovered (1-2 hours). During recovery the sedated monkeys will be observed by the field veterinarian from a distance from the trap so as to minimize stress on the animals. When they are completely recovered, the door will be re-opened to allow the monkeys to exit.
