# Supplementary material for: No evidence for sylvatic cycles of chikungunya, dengue and Zika viruses in African green monkeys (Chlorocebus aethiops sabaeus) on St. Kitts, West Indies
Source: Parasit Vectors. 2020 Oct 30;13:540. doi: 10.1186/s13071-020-04419-1 (PMC7598228; doi:10.1186/s13071-020-04419-1)
Supplement: Supplementary file 2 — Additional file 2: Text S2. Blood-meal analysis. [file 13071_2020_4419_MOESM2_ESM.docx]

**ADDITIONAL FILE 2 Text S2. Blood meal analysis**

**1) Table of individual mosquito identifier number and species.**

| Mosquito number | Mosquito species |
| --- | --- |
| 1 | *Aedes taeniorhynchus* |
| 2 | *Aedes taeniorhynchus* |
| 3 | *Aedes sp.* |
| 4 | *Culex sp.* |
| 5 | *Aedes sp.* |
| 6 | *Aedes aegypti* |
| 7 | *Aedes aegypti* |
| 8 | *Culex sp.* |
| 9 | *Culex sp.* |
| 10 | *Culex quinquefasciatus* |
| 11 | *Culex sp.* |
| 12 | *Aedes sp.* |
| 13 | *Culex sp.* |
| 14 | *Aedes sp.* |
| 15 | *Culex sp.* |
| 16 | *Aedes aegypti* |
| 17 | *Culex sp.* |
| 18 | *Culex sp.* |
| 19 | *Culex sp.* |
| 20 | *Culex sp.* |
| 21 | *Culex quinquefasciatus* |
| 22 | *Culex quinquefasciatus* |
| 23 | *Culex quinquefasciatus* |
| 24 | *Culex sp.* |
| 25 | *Culex quinquefasciatus* |
| 26 | *Aedes aegypti* |
| 27 | *Aedes aegypti* |
| 28 | *Psorophora pygmaea* |
| 29 | *Aedes aegypti* |
| 30 | *Culex quinquefasciatus* |
| 31 | *Culex sp.* |
| 32 | *Culex quinquefasciatus* |
| 33 | *Aedes taeniorhynchus* |
| 34 | *Aedes taeniorhynchus* |
| 35 | *Culex quinquefasciatus* |
| 36 | *Culex quinquefasciatus* |
| 37 | *Aedes aegypti* |
| 38 | *Culex quinquefasciatus* |
| 39 | *Culex sp.* |
| 40 | *Culex quinquefasciatus* |
| 41 | *Culex quinquefasciatus* |
| 42 | *Culex quinquefasciatus* |
| 43 | *Culex sp.* |
| 44 | *Culex sp.* |
| 45 | *Culex sp.* |
| 46 | *Culex sp.* |
| 47 | *Culex sp.* |
| 48 | *Culex quinquefasciatus* |
| 49 | *Culex quinquefasciatus* |
| 50 | *Aedes aegypti* |
| 51 | *Aedes aegypti* |
| 52 | *Aedes aegypti* |
| 53 | *Aedes taeniorhynchus* |
| 54 | *Aedes aegypti* |
| 55 | *Culex sp.* |
| 56 | *Culex sp.* |
| 57 | *Aedes sp.* |
| 58 | *Culex sp.* |
| 59 | *?* |
| 60 | *Culex sp.* |
| 61 | *Culex sp.* |
| 62 | *Culex sp.* |
| 63 | *Aedes aegypti* |
| 64 | *?* |
| 65 | *?* |
| 66 | *Culex sp.* |
| 67 | *Culex sp.* |
| 68 | *Culex sp.* |
| 69 | *Culex sp.* |
| 70 | *?* |
| 71 | *?* |
| 72 | *?* |
| 73 | *Culex quinquefasciatus* |
| 74 | *Culex sp.* |
| 75 | *Culex quinquefasciatus* |
| 76 | *?* |
| 77 | *Culex quinquefasciatus* |
| 78 | *Culex sp.* |
| 79 | *Culex sp.* |
| 80 | *Culex quinquefasciatus* |
| 81 | *Aedes sp.* |
| 82 | *Culex* |
| 83 | *Aedes taeniorhynchus* |
| 84 | *Culex sp.* |
| 85 | *Aedes taeniorhynchus* |
| 86 | *Aedes sp.* |
| 87 | *Aedes taeniorhynchus* |
| 88 | *Aedes sp.* |
| 89 | *Aedes taeniorhynchus* |
| 90 | *Aedes taeniorhynchus* |
| 91 | *Culex quinquefasciatus* |
| 92 | *Culex sp.* |
| 93 | *Culex sp.* |
| 94 | *Culex sp.* |
| 95 | *Aedes taeniorhynchus* |
| 96 | *?* |
| 97 | *?* |
| 98 | *Culex quinquefasciatus* |
| 99 | *Culex sp.* |
| 100 | *Culex quinquefasciatus* |
| 101 | *Culex sp.* |
| 102 | *Culex quinquefasciatus* |
| 103 | *Aedes aegypti* |
| 104 | *Culex quinquefasciatus* |
| 105 | *Culex sp.* |
| 106 | *Aedes taeniorhynchus* |
| 107 | *Culex sp.* |
| 108 | *Aedes aegypti* |
| 109 | *Culex sp.* |
| 110 | *Culex quinquefasciatus* |
| 111 | *Culex quinquefasciatus* |
| 112 | *Culex quinquefasciatus* |
| 113 | *Culex quinquefasciatus* |
| 114 | *Culex quinquefasciatus* |
| 115 | *Culex quinquefasciatus* |
| 116 | *Culex quinquefasciatus* |
| 117 | *Culex quinquefasciatus* |
| 118 | *Culex quinquefasciatus* |
| 119 | *Culex quinquefasciatus* |
| 120 | *Culex quinquefasciatus* |
| 121 | *Culex quinquefasciatus* |
| 122 | *Culex quinquefasciatus* |

**2) Aligned positive controls from AGM blood (CLUSTAL O. Madeira et al 2019).**

MV_589246-1001_POS_HMBSrevA01.ab1 ------AGCAGTGATGCCTACCAGCTGTGGGTCATCCTCAGGGCCATCTTCATGCTGTAT 54

MV_589246-2004_PosMB_HMBSrev_A04.ab1 GTTACGAGCAGTGATGCCTACCAGCTGTGGGTCATCCTCAGGGCCATCTTCATGCTGTAT 60

MV_589246-2001_POS1_HMBSrev_A01.ab1 GTTACGAGCAGTGATGCCTACCAGCTGTGGGTCATCCTCAGGGCCATCTTCATGCTGTAT 60

MV_589246-2003_POS3_HMBSrev_A03.ab1 GTTACGAGCAGTGATGCCTACCAGCTGTGGGTCATCCTCAGGGCCATCTTCATGCTGTAT 60

MV_589246-2002_POS2_HMBSrev_A02.ab1 GTTACGAGCAGTGATGCCTACCAGCTGTGGGTCATCCTCAGGGCCATCTTCATGCTGTAT 60

******************************************************

MV_589246-1001_POS_HMBSrevA01.ab1 GCGGGAAGGAGGTGGGAATTGGTGAAAACAAATGAGATTATATGCACTCCTGTTTATTAC 114

MV_589246-2004_PosMB_HMBSrev_A04.ab1 GCGGGAAGGAGGTGGGAATTGGTGAGAACAAATGAGATTATATGCACTCCTGTTTATTAC 120

MV_589246-2001_POS1_HMBSrev_A01.ab1 GCGGGAAGGAGGTGGGAATTGGTGAGAACAAATGAGATTATATGCACTCTTGTTTATTAC 120

MV_589246-2003_POS3_HMBSrev_A03.ab1 GCGGGAAGGAGGTGGGAATTGGTGAGAACAAATGAGATTATATGCACTCTTGTTTATTAC 120

MV_589246-2002_POS2_HMBSrev_A02.ab1 GCGGGAAGGAGGTGGGAATTGGTGAGAACAAATGAGATTATATGCACTCCTGTTTATTAC 120

************************* *********************** **********

MV_589246-1001_POS_HMBSrevA01.ab1 CCCCTCACCCTCCAGCTTTGGTACCTGGGCAGGGACATGGATGGTAGCCTGCATGGTGTC 174

MV_589246-2004_PosMB_HMBSrev_A04.ab1 CCCCTCACCCTCCAGCTTTGGTACCTGGGCAGGGACATGGATGGTAGCCTGCATGGTGTC 180

MV_589246-2001_POS1_HMBSrev_A01.ab1 CCCCTCACCCTCCAGCTTTGGTACCTGGGCAGGGACATGGATGGTAGCCTGCATGGTGTC 180

MV_589246-2003_POS3_HMBSrev_A03.ab1 CCCCTCACCCTCCAGCTTTGGTACCTGGGCAGGGACATGGATGGTAGCCTGCATGGTGTC 180

MV_589246-2002_POS2_HMBSrev_A02.ab1 CCCCTCACCCTCCAGCTTTGGTACCTGGGCAGGGACATGGATGGTAGCCTGCATGGTGTC 180

************************************************************

MV_589246-1001_POS_HMBSrevA01.ab1 TTGTATGCTATCTGAGCCATCTAGACTCCAGACTCCTCCAGTCAGGTAAA 224

MV_589246-2004_PosMB_HMBSrev_A04.ab1 TTGTATGCTATCTGAGCCATCTAGACTCCAGACTCCTCCNGTCAGGTAAA 230

MV_589246-2001_POS1_HMBSrev_A01.ab1 TTGTATGCTATCTGAGCCATCTAGACTCCAGACTCCTCCAGTCAGGTAAA 230

MV_589246-2003_POS3_HMBSrev_A03.ab1 TTGTATGCTATCTGAGCCATCTAGACTCCAGACTCCTCCAGTCAGGTAAA 230

MV_589246-2002_POS2_HMBSrev_A02.ab1 TTGTATGCTATCTGAGCCATCTAGACTCCAGACTCCTCCAGTCAGGTAAA 230

*************************************** **********

**3) Consensus AGM HMBS gene sequence (prior to GenBank submission).**

>REV_AGM_CON_SEQ_1

GTTACGAGCAGTGATGCCTACCAGCTGTGGGTCATCCTCAGGGCCATCTTCATGCTGTATGCGGGAAGGAGGTGGGAATTGGTGAGAACAAATGAGATTATATGCACTCNTGTTTATTACCCCCTCACCCTCCAGCTTTGGTACCTGGGCAGGGACATGGATGGTAGCCTGCATGGTGTCTTGTATGCTATCTGAGCCATCTAGACTCCAGACTCCTCCAGT

**4) Consensus AGM HMBS gene sequence (REV_AGM_CON_SEQ_1) and human reference sequence HMBS gene pairwise alignement in BLASTn.SNPs identified with ~.**

Query 1 : REV_AGM_CON_SEQ_1 Query ID: lcl|Query_63767 Length: 222

Sbjct

>Homo sapiens hydroxymethylbilane synthase (HMBS), RefSeqGene (LRG_1076) on chromosome 11

Sequence ID: NG_008093.1 Length: 15673

Range 1: 13068 to 13287

Score:367 bits(191), Expect:1e-97,

Identities:213/222(96%), Gaps:2/222(0%), Strand: Plus/Minus

Query 1 GTTACGAGCAGTGATGCCTACCAGCTGTGGGTCATCCTCAGGGCCATCTTCATGCTGTAT 60

|||||||||||||||||||||||~||||||||||||||||||||||||||||||||||||

Sbjct 13287 GTTACGAGCAGTGATGCCTACCAACTGTGGGTCATCCTCAGGGCCATCTTCATGCTGTAT 13228

Query 61 GCGGGAAGGAGGTGGGAATTGGTGAGAACAAATGAGATTATATGCACTCNTGTTTATTAC 120

|~|||||||||||||||~|||||||||||||~~|||||||||||||||| ||||||||||

Sbjct 13227 GAGGGAAGGAGGTGGGATTTGGTGAGAACAA--GAGATTATATGCACTCTTGTTTATTAC 13170

Query 121 CCCCTCACCCTCCAGCTTTGGTACCTGGGCAGGGACATGGATGGTAGCCTGCATGGTGTC 180

||||||~||||||||||||||||||||||||||||||||||||||||||||||||||~||

Sbjct 13169 CCCCTCGCCCTCCAGCTTTGGTACCTGGGCAGGGACATGGATGGTAGCCTGCATGGTCTC 13110

Query 181 TTGTATGCTATCTGAGCCATCTAGACTCCAGACTCCTCCAGT 222

||||||||||||||||||~|||||||||||||||||||||||

Sbjct 13109 TTGTATGCTATCTGAGCCGTCTAGACTCCAGACTCCTCCAGT 13068

**5)Aligned useable blood meal sequences (n=106) and positive controls in bold from AGM blood (n=5), only first 60 bp shown. Blood meals subsequently determined to be of *human origin in italics. Individual mosquito identifying number 43, 64, 65 and 106 highlighted in yellow)***

**43= *Culex* spp from mangrove land cover.**

**64= Unknown species from urban land cover.**

**65= Unknown species from urban land cover.**

**106=** ***Ae. taeniorhynchus*.**

**.**

MV_589246-1070_68_HMBSrevF10.ab1 TGCATGGAATCCTGTATGCTATCTGANACTCCTAAACCCCCNACTCCTGNAATCAGGTAACCT

MV_589246-1009_7_HMBSrevA09.ab1 TACCATAAAAACGGTAACAGACCTTAGAC--------TCCAGACTCCTCCAGTCAGGTAANTN

MV_589246-1039_37_HMBSrevD03.ab1 TACCATAGAAACGGTAACAGACCTTAGAC--------TCCAGACTCCTCCAGTCAGGTAAATG MV_589246-1052_50_HMBSrevE04.ab1 TACCATTGAACTGGTAAAAGACCGTAAAC--------TCCANACTCCTCCAGTCAGGTAACTG

MV_589246-1018_16_HMBSrevB06.ab1 TACCAGTAAAACGGTAACAGACCTTAGAC--------TCCAGACTCCTCCAGTCAGGTAANCT

MV_589246-1056_54_HMBSrevE08.ab1 CTCCAGTAAAACGGTAACAGAACTTAGAC--------TCCAGACTCCTCCAGTCAGGTAANNG

MV_589246-1053_51_HMBSrevE05.ab1 GTCAGGTAAACCGGNNACAGACCTTAGAC--------GCCAGACTCCTCCAGTCAGGTAANNA

MV_589246-1054_52_HMBSrevE06.ab1 GTCAGGTAAAACGGTAACNGACCTTANAC--------TCCNGACTCCTCNANTCCGGTAATCT

MV_589246-1071_69_HMBSrevF11.ab1 CACAAGTAAAACGGTAACAGACGTTAGAG--------TCCAGACTCCTCCAGTCAGGTAANAG

MV_589246-1069_67_HMBSrevF09.ab1 NTCAGGTAACCTGAANGCCGGATNNCTNC--------NTGGGACTCCTGACCAAATCCCAANT

MV_589246-1065_63_HMBSrevF05.ab1 GTAAGGTAATTCTTCTCTCGGCACGNTCC--------GCTAGACTCCCTTCTTCCCCGATCCT

MV_589246-1008_6_HMBSrevA08.ab1 GTCAGGTAANACGGTNTNNA-CCTTANAC--------TCCAGACTCCTCGGATCAGGTAAATA

MV_589246-2019_108_HMBSrev_B07.ab1 GTCAGGTAAAACGGCAACAGACCTTANAC--------TCCAGACTCCTCCAGTCAGGTAAACN

*MV_589246-1067_65_HMBSrevF07.ab1 TGCATGGTCTCTTGTATGCTATCTGAACCGGCTAGACACCNGACTCCTCCAGTCAGGTAATNN*

*MV_589246-1066_64_HMBSrevF06.ab1 TGCATGGTCTCTTGTATGCTATCTGAGCCGTCTAGACTCCAGACTCCTCCAGTCAGGTAANCC*

**MV_589246-2002_POS2_HMBSrev_A02.ab1 TGCATGGTGTCTTGTATGCTATCTGAGCCATCTAGACTCCAGACTCCTCCAGTCAGGTAAANN**

**MV_589246-2004_PosMB_HMBSrev_A04.ab1 TGCATGGTGTCTTGTATGCTATCTGAGCCATCTAGACTCCAGACTCCTCCNGTCAGGTAAANN**

**MV_589246-1001_POS_HMBSrevA01.ab1 TGCATGGTGTCTTGTATGCTATCTGAGCCATCTAGACTCCAGACTCCTCCAGTCAGGTAAATA**

**MV_589246-2001_POS1_HMBSrev_A01.ab1 TGCATGGTGTCTTGTATGCTATCTGAGCCATCTAGACTCCAGACTCCTCCAGTCAGGTAAAAC**

**MV_589246-2003_POS3_HMBSrev_A03.ab1 TGCATGGTGTCTTGTATGCTATCTGAGCCATCTAGACTCCAGACTCCTCCAGTCAGGTAAANN**

*MV_589246-1045_43_HMBSrevD09.ab1 TGCATGGTCTCTTGTATGCTATCTGAGCCGTCTAGACTCCAGACTCCTCCAGTCAGGTAANGN*

*MV_589246-2017_106_HMBSrev_B05.ab1 TGCATGGTCTCTTGTATGCTATCTGAGCCGTCTAGACTCCAGACTCCTCCAGTCAGGTAAAGN*

MV_589246-1021_19_HMBSrevB09.ab1 -----CATAGCTAACAGATGGAAGGCTGACATTACACTCCTCACTCCTCCAGGCAAGTAAGAT

MV_589246-2033_122_HMBSrev_C09.ab1 --------GCCTTAATTGTGCAAACCACGCTCTNCACTCCNANTCCTCCAGT-CGGGTAACTC

MV_589246-2014_103_HMBSrev_B02.ab1 --------ACCTTACTTGTTCA-CCCNCGCNCTAGACTCCATACTCCTNGGGTCAAGTAAANN

MV_589246-1058_56_HMBSrevE10.ab1 --------CCCTTAATTGCGCGAACCACGCTCTACACTCCNAACTCCTCCGGTCAGGTAAATG

MV_589246-1086_84_HMBSrevH02.ab1 --------ACCTTAATTGTGCAAACCACGCTCTAGACTCCAGACTCCTCCAGTCAGGTAAANN

MV_589246-1075_73_HMBSrevG03.ab1 GTTGACTTTTGTATTCTTGTAACTGGGACANNGATNACAGGTTNTCTTCCAGTAAGGAAGTGT

MV_589246-2007_96_HMBSrev_A07.ab1 CAGA---------CTCTTCTC--TCTCAGTAACNGACTCCAGACTCCTCCAGTCAGGTAANGT

MV_589246-1085_83_HMBSrevH01.ab1 CTGGNAAATACTGCTACCCCT--TCAAACCTCTTCACTGGCGAGGGAAACAGTAACGTAAACT

MV_589246-1055_53_HMBSrevE07.ab1 CATGGATTTATCGCGAATGCG--GGACTCCTTGAGACTTTCNACTTCTCCATTAAGGTAATTC

MV_589246-1087_85_HMBSrevH03.ab1 CATGNTTATACCGCGGATGTG--AAAATCCTTNCGACTCCCNACACCTCCATTAACGTAATCT

MV_589246-1003_1_HMBSrevA03.ab1 CAGANTTATANCGCGACTGCG--AAAAAACTTGAGACTCCCNACTCCTCCNNTCAGGTAATCN

MV_589246-1029_27_HMBSrevC05.ab1 CANGNTTATACCGCGAAAGCG--AAAATCCTTGAGACTCCCNACTCCTCCNNTAAGGTAATCN

MV_589246-1005_3_HMBSrevA05.ab1 CATGATTATATCGCGAATGCG--AACCTCCTTGAGACTCCCNACTCCTCCATTAAGGTAATCG

MV_589246-1035_33_HMBSrevC11.ab1 CAGAGTTTTATCGCGACTGCG--AAAATCCTTGAGACTCTCNACTCCTCCNTTCAGGTAATCN

MV_589246-1091_89_HMBSrevH07.ab1 CCTGAATAGATCGTGGATGTC--CGACTCCTCGCGTTGTTCTACATGAGCATTAACGTAATCG

MV_589246-1004_2_HMBSrevA04.ab1 CCTGAATATATCGTGGATGTC--CAACTCCTCGCGTTGTTCTACGCGTCCNTTAACGTAATCG

MV_589246-1088_86_HMBSrevH04.ab1 CATGATTATATCACGGATGAC--CGACTCCTTGCGATGTTCTACTCCTCCATTAACGTAATCG

MV_589246-1077_75_HMBSrevG05.ab1 -------TTCGGGCTGCTTCATAACTGAGNGTCCGACTCCGACTCAT-NCGGTAGNGNGANGA

MV_589246-1060_58_HMBSrevE12.ab1 -------ATCCGCCTCCTGGGTAANGGTGAGTCCCACTGTGGAACNA-GCCGTCCNGTGTGNA

MV_589246-1095_93_HMBSrevH11.ab1 -------GAACGCCTATTGGACAAGTCTGAGTCTCACTGCAGCAACATGTGGGCCGCCAANNA

MV_589246-1073_71_HMBSrevG01.ab1 -------ACCGGCCTCATGCATACATCTGAACCTGACTCCAGAAACCTGCCGTCAGGTAAGAC

MV_589246-1027_25_HMBSrevC03.ab1 -------ANTGGGGTGACTCATCTCTCAGTACCAGACTCCAGACTCCTCCAGTCAGGTAAGGA

MV_589246-1044_42_HMBSrevD08.ab1 -------ANAGGCGTGACTCATAACTCAGAACCNNACTCCAGACTCCTCCAGTCAGGTAAGAC

MV_589246-1057_55_HMBSrevE09.ab1 ----------GNCTTANGCAAGAACCAGGTAANTGTTATGTAAAGCTGTCCCTCTGCACAGAA

MV_589246-1059_57_HMBSrevE11.ab1 ----------CCTTCCCCTCNCTCCTTCCTGGCAGACTCCAGACTCCTCCAGTCAGGTAAGNN

MV_589246-1030_28_HMBSrevC06.ab1 --TCGTCGATTTCGACTTCTTCGACTTCGACTCNGACTCCNCACTCCTCCAGTCAGGTAATAA

MV_589246-1012_10_HMBSrevA12.ab1 ---TGATCTGGCTACAACCAGCAGCCATGGACTANACTCCAGACTCCTCCAGTCAGGTAAAGT

MV_589246-2013_102_HMBSrev_B01.ab1 ---TGATCTGGCTACAACCAGCAGCCATGGACTAGACTCCAGACTCCTCCAGTCAGGTAAAGT

MV_589246-1041_39_HMBSrevD05.ab1 ---TGATCTGGNNACNACCAGCAGCCATGGACTAGACTCCAGACTCCTCCAGTCAGGTAATGG

MV_589246-1062_60_HMBSrevF02.ab1 ---TGATCTGGCTACTACCAGCAGCCATGGACTAGACTCCAGACTCCTCCAGTCAGGTAATGG

MV_589246-1093_91_HMBSrevH09.ab1 -------ACACAGCCATCCAGCATGGTCCAGAGAGACTCCAGACTCCTCCAGTCAGGTAAGTT

MV_589246-1016_14_HMBSrevB04.ab1 --CT---CCTCCAGTCAGGTAANGATCAGAGAGAGACTCCNGACTCCTCCAGTCGGGTAATNT

MV_589246-1064_62_HMBSrevF04.ab1 --GATCATGGCCACGCGCCTGCCCTCGGCCTGTAGACTCCAGACTCCTCCAGTCAGGTAAANN

MV_589246-2026_115_HMBSrev_C02.ab1 --GGCNACTCCGCAGTCGTCCATGCTGGTAANCNGACTCCANACTCCTCCANTCAGGTAANGG

MV_589246-1026_24_HMBSrevC02.ab1 ----------CTCTGTCAAGTCCAGCTATGGCCAGACTCCAGACTCCTCCAGTCAGGTAAANN

MV_589246-1017_15_HMBSrevB05.ab1 --CAG--CACAGG-GCTCAGCTCAGGGGCAGACAGACTCCAGACTCCTCCAGTCAGGNNNNNC

MV_589246-1033_31_HMBSrevC09.ab1 --CAG--CACGGG-GCTCAGCTCAGGGGCAGACAGACTCCAGACTCCTCCAGTCAGGTAANNN

MV_589246-2018_107_HMBSrev_B06.ab1 --CAG--CACGGTAANTCAGCTCAGGGGCAGACAGACTCCAGACTCCTCCAGTCAGGTAANNG

MV_589246-2030_119_HMBSrev_C06.ab1 --CAG--CACGGG-GCTCAGCTCAGGGGCAGACAGACTCCAGACTCCTCCAGTCAGGTAAAAC

MV_589246-1047_45_HMBSrevD11.ab1 --GGG-----GCTTAAACTGCTC---CATCCAAAGACTCCAGACTCCTCCAGTCAGGTAANNG

MV_589246-1078_76_HMBSrevG06.ab1 --GGG-----GCTTAAACTGCTC---CATCCAAAGACTCCAGACTCCTCCAGTCAGGTAAAGG

MV_589246-1094_92_HMBSrevH10.ab1 --CGG-----GCTTAAACTGCTC---CATCCAAAGACTCCAGACTCCTCCAGTCAGGTAAGGG

MV_589246-1051_49_HMBSrevE03.ab1 ---------------ACCCGATACAAAAAATTTNTNATTCCACCGCATAAGGAAANGGGAAAC

MV_589246-1036_34_HMBSrevC12.ab1 --GTG--GGATCATAGGCAGCCCCAAGAGGCCCAGACTCCAGACTCCTCCAGTCAGGTAACCN

MV_589246-1061_59_HMBSrevF01.ab1 ACTCTGGAGTGGTGTGACTCATCTCTCAGTACCAGACTCCAGACTCCTCCAGTCAGGTAANGG

MV_589246-1080_78_HMBSrevG08.ab1 ACTCTGGANNGNTGTGACTCATCTCTCAGTACCAGACTCCAGACTCCTCCAGTCAGGTAATGG

MV_589246-1038_36_HMBSrevD02.ab1 ACTCTGGAATGGGGTGACTCATCTCTCAATTCCCGAATCCCNAATCCTCCCGTCAGGGAAANT

MV_589246-1083_81_HMBSrevG11.ab1 ATTCTGGATTGGGGTGACTCATATCTCAATTCCTGACTCCCGACTCCTCCAGTCAGGTAAACT

MV_589246-1050_48_HMBSrevE02.ab1 ACTCTGGAGTGGTGTGACTCATCTCTCAGTACCAGACTCCAGACTCCTCCAGTCAGGTAANAC

MV_589246-2028_117_HMBSrev_C04.ab1 CCTCTGGAGTGGTGTGACTCATCTCTCAGTACCAGACTCCAGACTCCTCCAGTCAGGTAANCT

MV_589246-2032_121_HMBSrev_C08.ab1 ACTCTGGAGTGGTGTGACTCATCTCTCAGTACCAGACTCCAGACTCCTCCAGTCAGGTAANGG

MV_589246-2012_101_HMBSrev_A12.ab1 ACTCTGGAGTGGTGTGACTCATCTCTCAGTACCAGACTCCAGACTCCTCCAGTCAGGTAAATT

MV_589246-1006_4_HMBSrevA06.ab1 ACTCTGGAGTGGTGTGACTCATCTCTCAGTACCAGACTCCAGACTCCTCCAGTCAGGTAA---

MV_589246-1014_12_HMBSrevB02.ab1 ACTCTGGAGTGGTGTGACTCATCTCTCAGTACCAGACTCCAGACTCCTCCAGTCAGGTAAACT

MV_589246-1049_47_HMBSrevE01.ab1 ACTCTANCCCCTNGTGANTCNTNNATNGNTACCAGACTCCAAACTCCTGNTTCCCGTAANGGT

MV_589246-1013_11_HMBSrevB01.ab1 ACTCTGGAGTGGTGTGACTCATCTCTCAGTACCAGACTCCAAACTCCTGNANTCANGTAATGG

MV_589246-1015_13_HMBSrevB03.ab1 ACTCTGGAGTGGTGTGACTCATCTCTCAGTACCAGACTCCAAACTCCACCAGTCAGGTAATGG

MV_589246-1010_8_HMBSrevA10.ab1 ACTCTGGAGTGGTGTGACTCATCTCTCAGTACCAGACTCCAGACTCCTGCAGTCAGGTAATGG

MV_589246-2029_118_HMBSrev_C05.ab1 ACTCTGGAGTGGTGTGACTCATCTCTCAGTACCAGACTCCAGACTCCTCCAGTCAGGTAAANG

MV_589246-1089_87_HMBSrevH05.ab1 ACTCTGGAGTGGTGTGACTCATCTCTCAGTACCAGACTCCAGACTCCTCCAGTCAGGTAANGG

MV_589246-2027_116_HMBSrev_C03.ab1 ACTCTGGAGTGGTGTGACTCATCTCTCAGTGCCAGACTCCAGACTCCTCCAGTCAGGTAANGG

MV_589246-2024_113_HMBSrev_B12.ab1 ACTCTGGAGTGGTGTGACTCATCTCTCAGTACCAGACTCCAGACTCCTCCAGTCAGGNNNNNN

MV_589246-1040_38_HMBSrevD04.ab1 ACTCTGGANTGNTGTGACTCATCTCTCAGTACCAGACTCCAGACTCCTCCAGTCAGGTAANNN

MV_589246-2021_110_HMBSrev_B09.ab1 ACTCTGGAGTGGTGTGACTCATCTCTCAGTACCAGACTCCAGACTCCTCCAGTCAGGTAAGGG

MV_589246-1042_40_HMBSrevD06.ab1 ACTCTGGAGTGGTGTGACTCATCTCTCAGTACCAGACTCCAGACTCCTCCAGTCAGGTAANCC

MV_589246-1007_5_HMBSrevA07.ab1 ACTCTGGAGTGGTGTGACTCATCTCTCAGTACCAGACTCCAGACTCCTCCAGTCAGGTAATGG

MV_589246-1090_88_HMBSrevH06.ab1 ACTCTGGANTGGTGTGACTCATCTCTCAGTACCAGACTCCAGACTCCTCCAGTCAGGTAAAGN

MV_589246-1022_20_HMBSrevB10.ab1 ACTCTGGANTGGTGTGACTCATCTCTCAGTACCAGACTCCAGACTCCTCCAGTCAGGNNNGNT

MV_589246-1037_35_HMBSrevD01.ab1 ACTCTGGAGTGGTGTGACTCATCTCTCAGTACCAGACTCCAGACTCCTCCAGTCAGGTAAACT

MV_589246-1072_70_HMBSrevF12.ab1 ACTCTGGAGTGGTGTGACTCATCTCTCAGTACCAGACTCCAGACTCCTCCAGTCAGGTAAACT

MV_589246-1046_44_HMBSrevD10.ab1 ACTCTGGAGTGGTGTGACTCATCTCTCAGTACCAGACTCCAGACTCCTCCAGTCAGGTAATTN

MV_589246-2016_105_HMBSrev_B04.ab1 ACTCTGGAGTGGTGTGACTCATCTCTCAGTACCAGACTCCAGACTCCTCCAGTCAGGTNNTTT

MV_589246-1074_72_HMBSrevG02.ab1 ACTCTGGANTGGTGTGACTCATCTCTCAGTACCAGACTCCAGACTCCTCCAGTCAGGTAAAGT

MV_589246-2025_114_HMBSrev_C01.ab1 ACTCTGGAGTGGTGTGACTCATCTCTCAGTACCAGACTCCAGACTCCTCCAGTCAGGTAAANG

MV_589246-2010_99_HMBSrev_A10.ab1 ACTCTGGAGTGGTGTGACTCATCTCTCAGTACCAGACTCCAGACTCCTCCAGTCAGGTAAACT

MV_589246-1096_94_HMBSrevH12.ab1 ACTCTGGAGTGGTGTGACTCATCTCTCAGTACCAGACTCCAGACTCCTCCAGTCAGGTAANCT

MV_589246-1023_21_HMBSrevB11.ab1 ACTCTGGAGTGGTGTGACTCATCTCTCAGTACCAGACTCCAGACTCCTCCAGTCAGGTAAAGN

MV_589246-2023_112_HMBSrev_B11.ab1 ACTCTGGAGTGGTGTGACTCATCTCTCAGTACCAGACTCCAGACTCCTCCANTCAGGTAAAAC

MV_589246-1084_82_HMBSrevG12.ab1 ACTCTGAATTGGTGTGACTCATCTCTCAATACCTGACTCCAAACTCCTCCAGTCTCGTAAACT

MV_589246-2008_97_HMBSrev_A08.ab1 ACTCTGGAGTGGTGTGACTCATCTCTCAGTACCAGACTCCAGACTCCTCCAGTCAGGTAAACT

MV_589246-1048_46_HMBSrevD12.ab1 ACTCTGGAGTGGTGTGACTCATCTCTCAGTACCAGACTCCAGACTCCTCCAGTCAGGTAANTG

MV_589246-1025_23_HMBSrevC01.ab1 ACTCTGGAGTGGTGTGACTCATCTCTCAGTACCAGACTCCAGACTCCTCCAGTCAGGTNNNNG

MV_589246-2022_111_HMBSrev_B10.ab1 ACTCTGGAGTGGTGTGACTCATCTCTCAGTACCAGACTCCAGACTCCTCCAGTCAGGTNNANT

MV_589246-2009_98_HMBSrev_A09.ab1 ACTCTGGAGTGGTGTGACTCATCTCTCAGTACCAGACTCCAGACTCCTCCAGTCAGGTAAACT

MV_589246-1011_9_HMBSrevA11.ab1 ACTCTGGAGTGGTGTGACTCATCTCTCAGTACCAGACTCCAGACTCCTCCAGTCAGGTAAGGG

MV_589246-2015_104_HMBSrev_B03.ab1 ACTCTGGAGTGGTGTGACTCATCTCTCAGTACCAGACTCCAGACTCCTCCAGTCAGGTAAACT

MV_589246-1043_41_HMBSrevD07.ab1 ACTCTGGAGTGGTGTGACTCATCTCTCAGTACCAGACTCCAGACTCCTCCAGTCAGGTAAACT

MV_589246-1024_22_HMBSrevB12.ab1 ATTCTGGAGTGGGGTGACTCATCTCTCAATACCAGACTCCNNACTCCTCCANNCANGTANNCT

MV_589246-2011_100_HMBSrev_A11.ab1 ACTCTGGAGTGGTGTGACTCATCTCTCAGTACCAGACTCCAGACTCCTCCAGTCAGGTAAACT

**6) Blood meals with successful reads in polymorphic region compared with human reference gene in BLASTn.**

**Mosquito number 43, 64, 65 and 106 had successful reads in bold and highlighted in yellow.**

**43= *Culex* spp from mangrove land cover.**

**64= Unknown species from urban land cover.**

**65= Unknown species from urban land cover.**

**106=** ***Ae. taeniorhynchus* from scrub.**

RID: ECEN5A7J114

Job Title:ref|NG_008093|

Program: BLASTN

Query: Homo sapiens hydroxymethylbilane synthase (HMBS), RefSeqGene (LRG_1076) on chromosome 11 ID: NG_008093.1(nucleic acid) Length: 15673

Subject #1:MV_589246-1045_**43**_HMBSrevD09.ab1 ID: lcl|Query_6450 Length: 160

Subject #2:MV_589246-1066_**64**_HMBSrevF06.ab1 ID: lcl|Query_6451 Length: 534

Subject #3:MV_589246-1067_**65**_HMBSrevF07.ab1 ID: lcl|Query_6452 Length: 799

Subject #4:MV_589246-2017_**106**_HMBSrev_B05.ab1 ID: lcl|Query_6453 Length: 707

Sequences producing significant alignments:

Max Total Query E Per.

Description Score Score cover Value Ident Accession

MV_589246-2017_**106**_HMBSrev_B05.ab1 420 567 1% 1e-119 100.00 Query_6453

MV_589246-1066_**64**_HMBSrevF06.ab1 372 372 1% 3e-105 97.64 Query_6451

MV_589246-1067_**65**_HMBSrevF07.ab1 195 195 1% 7e-52 83.25 Query_6452

MV_589246-1045_**43**_HMBSrevD09.ab1 110 110 0% 2e-26 86.87 Query_6450

Alignments:

**Mosquito 106 *Ae. taeniorhynchus* from scrub land cover.**

>MV_589246-2017_**106**_HMBSrev_B05.ab1

Sequence ID: Query_6453 Length: 707

Range 1: 18 to 244

Score:420 bits(227), Expect:1e-119,

Identities:227/227(100%), Gaps:0/227(0%), Strand: Plus/Minus

Query 13062 TACCTGACTGGAGGAGTCTGGAGTCTAGACGGCTCAGATAGCATACAAGAGACCATGCAG 13121

||||||||||||||||||||||||||||||||||||||||||||||||||||||||||||

Sbjct 244 TACCTGACTGGAGGAGTCTGGAGTCTAGACGGCTCAGATAGCATACAAGAGACCATGCAG 185

Query 13122 GCTACCATCCATGTCCCTGCCCAGGTACCAAAGCTGGAGGGCGAGGGGGTAATAAACAAG 13181

||||||||||||||||||||||||||||||||||||||||||||||||||||||||||||

Sbjct 184 GCTACCATCCATGTCCCTGCCCAGGTACCAAAGCTGGAGGGCGAGGGGGTAATAAACAAG 125

Query 13182 AGTGCATATAATCTCTTGTTCTCACCAAATCCCACCTCCTTCCCTCATACAGCATGAAGA 13241

||||||||||||||||||||||||||||||||||||||||||||||||||||||||||||

Sbjct 124 AGTGCATATAATCTCTTGTTCTCACCAAATCCCACCTCCTTCCCTCATACAGCATGAAGA 65

Query 13242 TGGCCCTGAGGATGACCCACAGTTGGTAGGCATCACTGCTCGTAACA 13288

|||||||||||||||||||||||||||||||||||||||||||||||

Sbjct 64 TGGCCCTGAGGATGACCCACAGTTGGTAGGCATCACTGCTCGTAACA 18

Range 2: 437 to 517

Score:147 bits(79), Expect:2e-37,

Identities:80/81(99%), Gaps:0/81(0%), Strand: Plus/Minus

Query 13062 TACCTGACTGGAGGAGTCTGGAGTCTAGACGGCTCAGATAGCATACAAGAGACCATGCAG 13121

||||||||||||||||||||||||||||||||||||||||||||||||||||||||||||

Sbjct 517 TACCTGACTGGAGGAGTCTGGAGTCTAGACGGCTCAGATAGCATACAAGAGACCATGCAG 458

Query 13122 GCTACCATCCATGTCCCTGCC 13142

||||||||||||||||| |||

Sbjct 457 GCTACCATCCATGTCCCNGCC 437

**Mosquito 64 Unknown species from urban land cover.**

>MV_589246-1066_**64**_HMBSrevF06.ab1

Sequence ID: Query_6451 Length: 534

Range 1: 35 to 246

Score:372 bits(201), Expect:3e-105,

Identities:207/212(98%), Gaps:0/212(0%), Strand: Plus/Minus

Query 13062 TACCTGACTGGAGGAGTCTGGAGTCTAGACGGCTCAGATAGCATACAAGAGACCATGCAG 13121

||||||||||||||||||||||||||||||||||||||||||||||||||||||||||||

Sbjct 246 TACCTGACTGGAGGAGTCTGGAGTCTAGACGGCTCAGATAGCATACAAGAGACCATGCAG 187

Query 13122 GCTACCATCCATGTCCCTGCCCAGGTACCAAAGCTGGAGGGCGAGGGGGTAATAAACAAG 13181

||||||||||||||||||||||||||||||||||||||||||||||||||||||||||||

Sbjct 186 GCTACCATCCATGTCCCTGCCCAGGTACCAAAGCTGGAGGGCGAGGGGGTAATAAACAAG 127

Query 13182 AGTGCATATAATCTCTTGTTCTCACCAAATCCCACCTCCTTCCCTCATACAGCATGAAGA 13241

||||||||||||||||||||||||||||||||||||||||||||||||||||||||||||

Sbjct 126 AGTGCATATAATCTCTTGTTCTCACCAAATCCCACCTCCTTCCCTCATACAGCATGAAGA 67

Query 13242 TGGCCCTGAGGATGACCCACAGTTGGTAGGCA 13273

||||||||||| | ||||||| ||||||||

Sbjct 66 TGGCCCTGAGGTTNNCCCACAGNNGGTAGGCA 35

**Mosquito 65 Unknown species from urban land cover.**

>MV_589246-1067_**65**_HMBSrevF07.ab1

Sequence ID: Query_6452 Length: 799

Range 1: 43 to 236

Score:195 bits(105), Expect:7e-52,

Identities:169/203(83%), Gaps:11/203(5%), Strand: Plus/Minus

Query 13062 TACCTGACTGGAGGAGTCTGGAGTCTAGACGGCTCAGATAGCATACAAGAGACCATGCAG 13121

|||||||||||||||||| || |||||| ||| |||||||||||||||||||||||||||

Sbjct 236 TACCTGACTGGAGGAGTCNGGTGTCTAGCCGGTTCAGATAGCATACAAGAGACCATGCAG 177

Query 13122 GCTACCATCCATGTCCCTGCCCAGGTACCAAAGCTGGAGGGCGAGGGGGTAATAAACAAG 13181

||||||||||||||||||||||||| |||||| ||||| ||||||||||||||||||

Sbjct 176 GCTACCATCCATGTCCCTGCCCAGGC-CCAAAGGN-GAGGGTGAGGGGGTAATAAACAAG 119

Query 13182 AGTGCATATAATCTC--TTGTTCTCACCAAATCCCACCTCCTTCCCTCATACAGCATGAA 13239

||||| || | | |||| | || | ||||||||||||||| || | | ||| |||

Sbjct 118 AGTGCCTANN-TNTNGGTTGT-CGCATC--ATCCCACCTCCTTCCNTC-TNCNGCA-GAA 65

Query 13240 GATGGCCCTGAGGATGACCCACA 13262

||| |||||| || |||| ||

Sbjct 64 GATC-CCCTGAAGAACACCCNCA 43

**Mosquito 43 *Culex* spp from mangrove land cover.**

>MV_589246-1045_**43**_HMBSrevD09.ab1

Sequence ID: Query_6450 Length: 160

Range 1: 63 to 160

Score:110 bits(59), Expect:2e-26,

Identities:86/99(87%), Gaps:1/99(1%), Strand: Plus/Minus

Query 13143 CAGGTACCAAAGCTGGAGGGCGAGGGGGTAATAAACAAGAGTGCATATAATCTCTTGTTC 13202

|||||||||||||||||||||||||||||| | ||||||| | ||||||| | |||

Sbjct 160 CAGGTACCAAAGCTGGAGGGCGAGGGGGTAGAATACAAGAGGGGATATAATTTGAAGTTA 101

Query 13203 TCACCAAATCCCACCTCCTTCCCTCATACAGCATGAAGA 13241

|||||||||||||| ||||||||||||||| || |||||

Sbjct 100 TCACCAAATCCCACGTCCTTCCCTCATACAACA-GAAGA 63

**7) Blood meals with successful reads in polymorphic region compared with AGM HMBS consensus sequence in BLASTn.**

**Mosquito number 43, 64, 65 and 106 had successful reads in bold and highlighted in yellow.**

**43= *Culex* spp from mangrove land cover.**

**64= Unknown species from urban land cover.**

**65= Unknown species from urban land cover.**

**106=** ***Ae. taeniorhynchus* from scrub.**

RID: ECH94RTX114

Job Title:REV_AGM_CON_SEQ_1

Program: BLASTN

Query: REV_AGM_CON_SEQ_1 ID: lcl|Query_39304(dna) Length: 222

Subject #1:MV_589246-1045_**43**_HMBSrevD09.ab1 ID: lcl|Query_39306 Length: 160

Subject #2:MV_589246-1066_**64**_HMBSrevF06.ab1 ID: lcl|Query_39307 Length: 534

Subject #3:MV_589246-1067_**65**_HMBSrevF07.ab1 ID: lcl|Query_39308 Length: 799

Subject #4:MV_589246-2017_**106**_HMBSrev_B05.ab1 ID: lcl|Query_39309 Length: 707

Sequences producing significant alignments:

Max Total Query E Per.

Description Score Score cover Value Ident Accession

MV_589246-2017_**106**_HMBSrev_B05.ab1 361 361 100% 8e-104 95.95 Query_39309

MV_589246-1066_**64**_HMBSrevF06.ab1 320 320 93% 1e-91 93.75 Query_39307

MV_589246-1067_**65**_HMBSrevF07.ab1 158 158 54% 1e-42 89.34 Query_39308

Alignments:

**Mosquito 106=** ***Ae. taeniorhynchus* from scrub.**

>MV_589246-2017_**106**_HMBSrev_B05.ab1

Sequence ID: Query_39309 Length: 707

Range 1: 19 to 238

Score:361 bits(195), Expect:8e-104,

Identities:213/222(96%), Gaps:2/222(0%), Strand: Plus/Plus

Query 1 GTTACGAGCAGTGATGCCTACCAGCTGTGGGTCATCCTCAGGGCCATCTTCATGCTGTAT 60

||||||||||||||||||||||| ||||||||||||||||||||||||||||||||||||

Sbjct 19 GTTACGAGCAGTGATGCCTACCAACTGTGGGTCATCCTCAGGGCCATCTTCATGCTGTAT 78

Query 61 GCGGGAAGGAGGTGGGAATTGGTGAGAACAAATGAGATTATATGCACTCNTGTTTATTAC 120

| ||||||||||||||| ||||||||||||| |||||||||||||||| ||||||||||

Sbjct 79 GAGGGAAGGAGGTGGGATTTGGTGAGAACAA--GAGATTATATGCACTCTTGTTTATTAC 136

Query 121 CCCCTCACCCTCCAGCTTTGGTACCTGGGCAGGGACATGGATGGTAGCCTGCATGGTGTC 180

|||||| |||||||||||||||||||||||||||||||||||||||||||||||||| ||

Sbjct 137 CCCCTCGCCCTCCAGCTTTGGTACCTGGGCAGGGACATGGATGGTAGCCTGCATGGTCTC 196

Query 181 TTGTATGCTATCTGAGCCATCTAGACTCCAGACTCCTCCAGT 222

|||||||||||||||||| |||||||||||||||||||||||

Sbjct 197 TTGTATGCTATCTGAGCCGTCTAGACTCCAGACTCCTCCAGT 238

**Mosquito 64= Unknown species from urban land cover.**

>MV_589246-1066_**64**_HMBSrevF06.ab1

Sequence ID: Query_39307 Length: 534

Range 1: 35 to 240

Score:320 bits(173), Expect:1e-91,

Identities:195/208(94%), Gaps:2/208(0%), Strand: Plus/Plus

Query 15 TGCCTACCAGCTGTGGGTCATCCTCAGGGCCATCTTCATGCTGTATGCGGGAAGGAGGTG 74

|||||||| ||||||| | |||||||||||||||||||||||||| ||||||||||||

Sbjct 35 TGCCTACCNNCTGTGGGNNAACCTCAGGGCCATCTTCATGCTGTATGAGGGAAGGAGGTG 94

Query 75 GGAATTGGTGAGAACAAATGAGATTATATGCACTCNTGTTTATTACCCCCTCACCCTCCA 134

||| ||||||||||||| |||||||||||||||| |||||||||||||||| |||||||

Sbjct 95 GGATTTGGTGAGAACAA--GAGATTATATGCACTCTTGTTTATTACCCCCTCGCCCTCCA 152

Query 135 GCTTTGGTACCTGGGCAGGGACATGGATGGTAGCCTGCATGGTGTCTTGTATGCTATCTG 194

||||||||||||||||||||||||||||||||||||||||||| ||||||||||||||||

Sbjct 153 GCTTTGGTACCTGGGCAGGGACATGGATGGTAGCCTGCATGGTCTCTTGTATGCTATCTG 212

Query 195 AGCCATCTAGACTCCAGACTCCTCCAGT 222

|||| |||||||||||||||||||||||

Sbjct 213 AGCCGTCTAGACTCCAGACTCCTCCAGT 240

**Mosquito 65= Unknown species from urban land cover.**

>MV_589246-1067_**65**_HMBSrevF07.ab1

Sequence ID: Query_39308 Length: 799

Range 1: 111 to 230

Score:158 bits(85), Expect:1e-42,

Identities:109/122(89%), Gaps:2/122(1%), Strand: Plus/Plus

Query 101 TATGCACTCNTGTTTATTACCCCCTCACCCTCCAGCTTTGGTACCTGGGCAGGGACATGG 160

|| |||||| |||||||||||||||||||||| |||||| |||||||||||||||||

Sbjct 111 TAGGCACTCTTGTTTATTACCCCCTCACCCTC-NCCTTTGG-GCCTGGGCAGGGACATGG 168

Query 161 ATGGTAGCCTGCATGGTGTCTTGTATGCTATCTGAGCCATCTAGACTCCAGACTCCTCCA 220

||||||||||||||||| ||||||||||||||||| || |||||| || ||||||||||

Sbjct 169 ATGGTAGCCTGCATGGTCTCTTGTATGCTATCTGAACCGGCTAGACACCNGACTCCTCCA 228

Query 221 GT 222

||

Sbjct 229 GT 230

**8) All useable blood meal sequences (entire sequence) aligned with consensus AGM sequence.**

Mosquito 1

REV_AGM_CON_SEQ_1 ------------------------------------------------GTTACGAGCAGT 12

MV_589246-1003_1_HMBSrevA03.ab1 NNNNNNNNNNNAANGNNNNCTCNGNTTCNNNNANGAGNTGTTTCCGGAGANTCGGTCGCT 60

* ** * *

REV_AGM_CON_SEQ_1 GATGCCTA----------CCAGCTGTGGGTCATCCTCAGGGCCATCTTCATGCTGT-ATG 61

MV_589246-1003_1_HMBSrevA03.ab1 GATTCCGAATAAATTGATCATCCTGGGCATCTTTTTNGAATGTATCTAACTTCNAGGTAC 120

*** ** * * *** * ** * * **** * *

REV_AGM_CON_SEQ_1 CGGGAAGGAGGTGGGAATTGGTGAGAACAAATGAGATTATATGCA--------------- 106

MV_589246-1003_1_HMBSrevA03.ab1 CANCCATGGCCTTGAGATTGATGAGGAAATCGGTGATTGACTGGTTGCCAAGCTGTTCGC 180

* * * * * **** **** * * * **** **

REV_AGM_CON_SEQ_1 ------------------------------------------------------------ 106

MV_589246-1003_1_HMBSrevA03.ab1 ATTTCCTAAACTTATACCTCTCCTCCAGGACGTTAGAGGTTGGAGCGAGGTTTTCCTTGA 240

REV_AGM_CON_SEQ_1 ------------------------------------------------CTCNTGTTTATT 118

MV_589246-1003_1_HMBSrevA03.ab1 AGAGAGTGAGGACATCTTGGTACGTTTTAGTACATGGATCAACAAGCGCACAGANTTATA 300

* * ****

REV_AGM_CON_SEQ_1 ACCCC------------------------------------------------------- 123

MV_589246-1003_1_HMBSrevA03.ab1 NCGCGACTGCGAAAAAACTTGAGACTCCCNACTCCTCCNNTCAGGTAATCNACCGGACTC 360

* *

REV_AGM_CON_SEQ_1 ------------------------------------------------------------ 123

MV_589246-1003_1_HMBSrevA03.ab1 TTCTCGGTGGTGCTCCGTCAAAAAATGCACCGTGCCAGTCGACTTTCTGCTGCGACTGCT 420

REV_AGM_CON_SEQ_1 ---------CTCACCCTCCAGCTTTGGTACCTGGG---CAGG------------------ 153

MV_589246-1003_1_HMBSrevA03.ab1 TCCCTTGTGCTCCTCTCTCAGGTTTNGNAGCTGCTGANCAGCTCANTGGTTACTACTCCG 480

*** * *** *** * * *** ***

REV_AGM_CON_SEQ_1 ------------------------------------------------------------ 153

MV_589246-1003_1_HMBSrevA03.ab1 ATGATTAATTGGGGTGTTATGCCGATGGACCTCNTNNNGCGCAATTTCTTCANGTGCGGA 540

REV_AGM_CON_SEQ_1 ------------------------------------------------------------ 153

MV_589246-1003_1_HMBSrevA03.ab1 TGAGGATAANATANNTCGNNNTACNCCNNNGGGTGNTNNNGTANNTTTANNNGNTCCATC 600

REV_AGM_CON_SEQ_1 ----------------GACATGGATGGTAGCCTGCATGGTGTCTTGTATGCTATCTGAGC 197

MV_589246-1003_1_HMBSrevA03.ab1 GTGCTGANTCTCAACAGACAGCTNNTGTTCTCANTNCGGACTNNNNTCNNNAATTCGAAG 660

**** ** * ** * * ** **

REV_AGM_CON_SEQ_1 CATCTAGACTCCAGACTCCTCCAGT------- 222

MV_589246-1003_1_HMBSrevA03.ab1 CGTTGAGACTCCAGACTCCTCCANTCANGTNN 692

* * ****************** *

Mosquito 2

REV_AGM_CON_SEQ_1 -------------------------------------------------GT-----TACG 6

MV_589246-1004_2_HMBSrevA04.ab1 NNNNNNNNNNNNNANATNCGCANNTACGNNTNNGAGNNTTTNNNCNAGTGTGACGCTNNT 60

** *

REV_AGM_CON_SEQ_1 AGCAGTGATGCCTACCAGCTGTGGGTCATCCTCAGGGCCATCTTCATGCTGTATGCGGGA 66

MV_589246-1004_2_HMBSrevA04.ab1 TCCCTNNANATAGACCATCTTGGACGNCTTCATTTNAGGGTCTCCATTCTACATACCGCC 120

* * **** ** * * * *** *** ** ** * *

REV_AGM_CON_SEQ_1 AGG-AGGTGGGAATTGGTGAGAACAAATGAG-----ATTATATGCACTCNTGTTTATTAC 120

MV_589246-1004_2_HMBSrevA04.ab1 ATTGGAGTAATGTCCGGTGAGCAAGGATCGGCGCGACTGGTTGNCAGTCCTGTCTCCTTC 180

* ** ****** * ** * * * ** ** *** * * *

REV_AGM_CON_SEQ_1 ------------------------------------------------------------ 120

MV_589246-1004_2_HMBSrevA04.ab1 TGCCATCNACCCTACGGGTACTGGACTGGAGAATCTTCNAGGCCCGTTTATCTTCCTCCG 240

REV_AGM_CON_SEQ_1 ----------------------CCCCTCACCCTCCAGCTTTGGTACCTGGGCAGGGACAT 158

MV_589246-1004_2_HMBSrevA04.ab1 ACTGAGGACATACTTTAATGCTATCCTCNTCCAACGCCATAGATTGCCTGAATATATCGT 300

**** ** * * * * * * * * *

REV_AGM_CON_SEQ_1 GGATGGTAGCCTGCAT-------------------------------------------- 174

MV_589246-1004_2_HMBSrevA04.ab1 GGATGTCCAACTCCTCGCGTTGTTCTACGCGTCCNTTAACGTAATCGACCGGACTCTTCT 360

***** ** *

REV_AGM_CON_SEQ_1 ------------------------------------------------------------ 174

MV_589246-1004_2_HMBSrevA04.ab1 CGGTGGCGCTCCGTCCGTAAATGCACCATCCCAGCCGAGTTTTTGCAGCGACCACTTCCC 420

REV_AGM_CON_SEQ_1 ------------------------------------------------------------ 174

MV_589246-1004_2_HMBSrevA04.ab1 CTGTGCTACTCTCTCGGCTTTTGAGCGTGCTGATCAGCTTANCGTTGTCTACTCCGATTA 480

REV_AGM_CON_SEQ_1 ------------------------------------------------------------ 174

MV_589246-1004_2_HMBSrevA04.ab1 TTAATTGTGGGGTTACATCGGTATAGCTCCTCAGGGGCAATTTCTTCAGGTGCGGATGAA 540

REV_AGM_CON_SEQ_1 ------------------------------------------------------------ 174

MV_589246-1004_2_HMBSrevA04.ab1 GATCACAAAGCTCGTCATAAACCACGGTTTGCTTGGGTAGTTTTAACTGCTCTACGGTCC 600

REV_AGM_CON_SEQ_1 ---------------------------------GGTGTCTTGTATGCTATCTGAGCCATC 201

MV_589246-1004_2_HMBSrevA04.ab1 TGGCTCTCAACGGAAAGCTCCTGTTCTCGTTACGGCCTGCAATCNNNANTNNNATGCGTT 660

** * * * * * *

REV_AGM_CON_SEQ_1 TAGACTCCAGACTCCTCCAGT--------- 222

MV_589246-1004_2_HMBSrevA04.ab1 GAGACTCCAGACTCCTCCNNNTCAGGTAAA 690

*****************

Mosquito 3

REV_AGM_CON_SEQ_1 ------------------------------------------------------------ 0

MV_589246-1005_3_HMBSrevA05.ab1 NNNNNNNNNNNNNNNNNGNANACGNNGNTACGACTCNAGACTTNTTTNNNANANNCGAAG 60

REV_AGM_CON_SEQ_1 ----GTTACGAGCAGTGATGCCTACCAGCTGTGGGTCATCCTCAGGGCCATCTTCATGCT 56

MV_589246-1005_3_HMBSrevA05.ab1 NANNAATCCGAGTNNNAGACCATACTCGACATCTTTTANTTCTGGCTCTAAATTC---CA 117

* **** * *** * * * * * * * *** *

REV_AGM_CON_SEQ_1 GTATGCGGGAAGGAGGTGGGAATTGGTGAGAACAAATGAGATTATATGCACTCNTGTT-- 114

MV_589246-1005_3_HMBSrevA05.ab1 GATTCCNNANNTGGACTTGTGATTGGTGATGAAATCTCGGATTGACTGGTTGGCAATCTG 177

* * * * * * ******** * * * **** ** *

REV_AGM_CON_SEQ_1 ------------TATTACCCCCTCACCCTCCAGCTTTGGTACCTGGGCAGGGACATGGAT 162

MV_589246-1005_3_HMBSrevA05.ab1 TTCTCATTTCCNAAACTTATACTCCTCCTCCAGGACTGGAGAATCTGGAGGNCCGTTNAA 237

* *** ******* *** * * *** * * *

REV_AGM_CON_SEQ_1 GGTAGCCTGCATGGTG-------------------------------------------- 178

MV_589246-1005_3_HMBSrevA05.ab1 CTTCCTCCGAATGAGGACATNTTGTAATGTTATACTACNTGAATCAACNAGCGCGCATGA 297

* * * *** *

REV_AGM_CON_SEQ_1 ------------------------------------------------------------ 178

MV_589246-1005_3_HMBSrevA05.ab1 TTATATCGCGAATGCGAACCTCCTTGAGACTCCCNACTCCTCCATTAAGGTAATCGACCG 357

REV_AGM_CON_SEQ_1 ------------------------------------------------------------ 178

MV_589246-1005_3_HMBSrevA05.ab1 GACTCTTCTCGGTGGCGCTCCGTCAATAAATGCACCGTCCCAGTCGACTTTTTGCAGCGA 417

REV_AGM_CON_SEQ_1 ------------------------------------------------------------ 178

MV_589246-1005_3_HMBSrevA05.ab1 CNACTTCCCCTGTGCTCCTCTCTCAGGTTTTGANNCTGTTGACCAGCTTACCGTTGACTA 477

REV_AGM_CON_SEQ_1 ------------------------------------------------------------ 178

MV_589246-1005_3_HMBSrevA05.ab1 CTCCGATTATTAANTGGGGTGTTACGATGGTAGACCTCCTGCGGCGCCACTTGNNCNNNT 537

REV_AGM_CON_SEQ_1 ------------------------------------------------------------ 178

MV_589246-1005_3_HMBSrevA05.ab1 GCGGATGAAGATCACNANCTCGTCATACACCCNGGGTGCTTGGGTAGTTTTAGCTGCTCT 597

REV_AGM_CON_SEQ_1 --------------------------------------------TCTTGTATGCTATCTG 194

MV_589246-1005_3_HMBSrevA05.ab1 ACGGTCCTGGCTCTCAACGGAAAGCTCCTGTTCTCGTTACGGCCTGCAATNNTAAATTAG 657

* * ** *

REV_AGM_CON_SEQ_1 AGCCATCTAGACTCCAGACTCCTCCAGT------ 222

MV_589246-1005_3_HMBSrevA05.ab1 AAGCGTTGAGACTCCANACTCCTCCANNCAGGTN 691

* * * ******** *********

Mosquito 4

REV_AGM_CON_SEQ_1 -----GTTACGAGCAGTGATGCCTACCAGCTGTGGGTCATCCTCAGGGCCATCTTCATGC 55

MV_589246-1006_4_HMBSrevA06.ab1 ANNNNNNCACGNNATNNCATAACTGCAGA---------CT--------CCAGACTCCTCC 43

*** ** ** * * *** ** * *

REV_AGM_CON_SEQ_1 TGTATGCGGGAAGGAGGT----GGGAATTGGTGAGAACAAATGAGATTATATGCACTCNT 111

MV_589246-1006_4_HMBSrevA06.ab1 AGTCAGGTAAAACTCTANCATCTCGNTTCAGNTCANNNNNNTGTGATAATGGCAGCTGTT 103

** * ** * * * ** *** ** ** *

REV_AGM_CON_SEQ_1 GTTTATTACCCCCTCACCCTCCAGCTTTGGTACCTGGGCAGGGACATGGATGGTAGCCTG 171

MV_589246-1006_4_HMBSrevA06.ab1 GATTACTTTTCTCCAGC----AG--------------------ACTCTTCTAGT----AC 135

* *** * * * * ** * **

REV_AGM_CON_SEQ_1 CATGGTGTCTTGTATGCTATCTGAGCCATCTAGACTCCAGACTCCTCCAGT------- 222

MV_589246-1006_4_HMBSrevA06.ab1 TCTGGAGTGGTGTGACTCATCTCTCAGTACCAGACTCCAGACTCCTCCAGTCAGGTAA 193

*** ** *** **** * ********************

Mosquito 5

REV_AGM_CON_SEQ_1 ---GT-----------TACGAGCAGTGATG--CCTACCAGCTGTGGG---TCATCCTCAG 41

MV_589246-1007_5_HMBSrevA07.ab1 NNNNNNNNNNNCNNNGNNNCNGCNNNACTNNNNANNCCTCCTGTCNNGTAANGGCGTAAC 60

** * ** **** * * *

REV_AGM_CON_SEQ_1 GGCCATCTTCATGCTGTAT--GCGGGAAGGAGGTGGGAATTGGTGAGAACAAA-TGAGAT 98

MV_589246-1007_5_HMBSrevA07.ab1 TTCGATACTCTTTAGCGATGTGATAATGGCAGCTGTTGATTACTTTTCTCCAGCAGACTC 120

* ** ** * ** * * ** ** *** * * * **

REV_AGM_CON_SEQ_1 TATATGCACTCNTGTTTATTACCCCCTCACCCTCCAGCTTTGGTACCTGGGCAGGGACAT 158

MV_589246-1007_5_HMBSrevA07.ab1 TTCTAGTACTCTGGAGTG-------GTGTGAC-TCATCTCTCAGTACCAGACTCCAGACT 172

* * **** * * * * ** ** * * * * *

REV_AGM_CON_SEQ_1 GGATGGTAGCCTGCATGGTGTCTTGTATGCTATCTGAGCCATCTAGACTCCAGACTCCTC 218

MV_589246-1007_5_HMBSrevA07.ab1 CCTCCAGTCAGGTAATGGTCTCTTGTATGCTATCTGAGCCGTCTAGACTCCAGACTCCTC 232

***** ******************** *******************

REV_AGM_CON_SEQ_1 CAGT-------------------------------------------------------- 222

MV_589246-1007_5_HMBSrevA07.ab1 CAGTCAGGTAATGCNGGCTGCTGTTGTTGGTGTGNTGGGGCGGGNAANGCGGNATTNACA 292

****

REV_AGM_CON_SEQ_1 ------------------------------------------------------------ 222

MV_589246-1007_5_HMBSrevA07.ab1 CNGNGGNGGNGGTCNNTNGAGGNCNGGGGNGACGGGCNGGNATCNATGTCGACGNCGTAT 352

REV_AGM_CON_SEQ_1 ------------------------------------------- 222

MV_589246-1007_5_HMBSrevA07.ab1 TAGNTCTCGNAAATAAGCANGGGCAGACTGTAGACNCCTCCGG 395

Mosquito 6

REV_AGM_CON_SEQ_1 ----------------------------------------------GTTACGAGCAGTGA 14

MV_589246-1008_6_HMBSrevA08.ab1 NNNNNNNNGNGANNNCGCCCTNNNNCCATTTGTAACTGTAGTATACCTATACGATAGTAA 60

* *** *

REV_AGM_CON_SEQ_1 TGCCTA------------------------------------------------------ 20

MV_589246-1008_6_HMBSrevA08.ab1 TGTGTGCACGAAGATCGATTGTCTTCGTGCGATGTTCTACCACATGGTAGAAATATTGCT 120

** *

REV_AGM_CON_SEQ_1 -------------------------CCAGCTGTGGGTCATCCTCAGGG------------ 43

MV_589246-1008_6_HMBSrevA08.ab1 CTTTATCTGTGATATCAATAAACGACTAGGAGTAGTTCATGTTCAGACTCCAGACTCCTC 180

* ** ** * **** ****

REV_AGM_CON_SEQ_1 ----C-----------------CATCTTCATGCTGTATGCGGGAAGGAGGT--------- 73

MV_589246-1008_6_HMBSrevA08.ab1 CAGTCAGGTAANACGGTNTNNACCTTANACTCCAGACTCCTCGGATCAGGTAAATATGGG 240

* * * * * * * * * * ****

REV_AGM_CON_SEQ_1 ------------------------------------------GGGAATTGGTGAGAACAA 91

MV_589246-1008_6_HMBSrevA08.ab1 CGGTGCCACGATAGTTAATTACATCGGATTTTTTGCCAGATTTGAAAATGGGAACTAGAT 300

* ** *** * * *

REV_AGM_CON_SEQ_1 ATGAGATTATATGCACTCNTGTTTATTACCCCCTCACCCTCCAGCTTTGGTACCTGGGCA 151

MV_589246-1008_6_HMBSrevA08.ab1 ATGAGCTTTT--CCACTTCTTTGGAAAGACTCCAGACTCCTCCNGTCAGGTAANNCTCCT 358

***** ** * **** * * * * ** ** * * * **** *

REV_AGM_CON_SEQ_1 GGGACATGGATGGT------------AGCCTGCA--------------TGGTGTCTTGTA 185

MV_589246-1008_6_HMBSrevA08.ab1 CTGGTATCGCCNGTCATCGGCNNGTACCTCTACTTGGCGTCCCATTTTCAGTATATAGGC 418

* ** * ** ** * ** * * *

REV_AGM_CON_SEQ_1 TGCTATCTGAGCC----------------------------------------------- 198

MV_589246-1008_6_HMBSrevA08.ab1 TGTTATCCCAGACTTGTAGGCTCTGAANTTNGATACNATGCTAATCGATCATTTTGAACG 478

** **** ** *

REV_AGM_CON_SEQ_1 ----------------------------------------ATCTAGACTCCAGACTCCTC 218

MV_589246-1008_6_HMBSrevA08.ab1 GTTGGTCCAATCATTTGCGTATTGTTGATCGACACGCCAGTAGTAGACTCCAGACTCCTC 538

*****************

REV_AGM_CON_SEQ_1 CAGT-------------------------------------------------------- 222

MV_589246-1008_6_HMBSrevA08.ab1 CAGTCAGGTAANNGATTTTCCNGATCAATGCATCTTGNTGAAAGACTCCATACTCCNCNN 598

****

REV_AGM_CON_SEQ_1 ----------------------------- 222

MV_589246-1008_6_HMBSrevA08.ab1 GTCANGTAANNGNGNNNGNANTNNNNNTN 627

Mosquito 7

REV_AGM_CON_SEQ_1 GTTACGAGCAGTGATGCCTACCAGCTGTGGGTCATCCTCAGGG----CCATCTTCATGCT 56

MV_589246-1009_7_HMBSrevA09.ab1 AATAGAAATTCTGAACCCGCCCCGCTGACTGCCGTTGGTCGGATCAACAATCTGGGGTTT 60

** * *** ** ** **** * * * ** * **** *

REV_AGM_CON_SEQ_1 GTATGCGGGAAGGAGGTGGGAATTGGTGAGAACAAAT-------GAGATTATATGCACTC 109

MV_589246-1009_7_HMBSrevA09.ab1 TGACCGGTTCCCCATCTGGGCTTCGGTGTCCGGCAAGAGAGCCGTACCATAAAAACGGTA 120

* * * **** * **** ** * ** * * *

REV_AGM_CON_SEQ_1 NTGTTTATTA-CCCCCTCACCCTCCAGCTTTGGTACCTGGG----CAGGGACATGGATGG 164

MV_589246-1009_7_HMBSrevA09.ab1 ACAGACCTTAGACTCCAGACTCCTCCAGTCAGGTAANTNANATTGGTGTGATNTCTTACG 180

*** * ** ** * * * **** * * ** * *

REV_AGM_CON_SEQ_1 TAGCCTGCATGGTGTCTTGTATGCTATCTGAGCCATCTAGACTCCAGACTCCTCCAGT-- 222

MV_589246-1009_7_HMBSrevA09.ab1 TACCCAGTAAGGCCATCCACCTTAGAGAGGGACCGCNGGGCACCNAGGTGGCGNCGATCA 240

** ** * * ** * * * ** * * ** * * *

REV_AGM_CON_SEQ_1 -------- 222

MV_589246-1009_7_HMBSrevA09.ab1 GGCTGCCG 248

Mosquito 8

REV_AGM_CON_SEQ_1 -GTTACGAGCAGTGATGCCTACCAGCTGT---------GGGTCATCCTCAGGGCCATCTT 50

MV_589246-1010_8_HMBSrevA10.ab1 NNNNNNNNNNNNNGNTGNNNGCGCNCTTTNCNTTAAGACCATCATCAGCAGTGTGGCGTA 60

* ** * ** * ***** *** * *

REV_AGM_CON_SEQ_1 CATGCTGTATGCGGGAAGGAGGTGGGAATTGGTGAGAACAAATGAGATTATAT-GCACTC 109

MV_589246-1010_8_HMBSrevA10.ab1 ACTTCGATACACTTTAGCGATGTGATAATGGCAGCTGTTGATTACTTTTCTCCAGCAGAC 120

* * ** * * ** *** *** * * * * ** * *** *

REV_AGM_CON_SEQ_1 NTGTTTATTACCCCCTCA-----CCCTCCAGCTTTGGTACCTGGGCAGGGACATGGATGG 164

MV_589246-1010_8_HMBSrevA10.ab1 TCTTCTAGTACTCTGGAGTGGTGTGACTCATCTCTCAGTACCAGACTCCAGACTCCTGCA 180

* ** *** * ** ** * * * * *

REV_AGM_CON_SEQ_1 TAGCCTGCATGGTGTCTTGTATGCTATCTGAGCCATCTAGACTCCAGACTCCTCCAGT-- 222

MV_589246-1010_8_HMBSrevA10.ab1 GTCAGGTAATGGTCTCTTGTATGCTATCTGAGCCGTCTAGACTCCAGACTCCTCCAGTCA 240

***** ******************** ***********************

REV_AGM_CON_SEQ_1 ------------------------------------------------------------ 222

MV_589246-1010_8_HMBSrevA10.ab1 GGTAAGGCATTCNCGTAATCGGTGCTCCCNCGGNNTGGTAANGNGACCCCTCTGGGNGTG 300

REV_AGM_CON_SEQ_1 ------------------------------------------------------------ 222

MV_589246-1010_8_HMBSrevA10.ab1 GATGGTNTNGTNACCCATGTGGCATNTACANANATTGCACTACAACCCCAGCCCAGATCT 360

REV_AGM_CON_SEQ_1 ------------------------------------------------------------ 222

MV_589246-1010_8_HMBSrevA10.ab1 CNAACAAAGCNGGCTCGNACTGTGNACTGGGCCTGAAACGTGGGATCAGAACCTGGGCAT 420

REV_AGM_CON_SEQ_1 ------------ 222

MV_589246-1010_8_HMBSrevA10.ab1 CAGCATGGCANN 432

Mosquito 9

REV_AGM_CON_SEQ_1 ------------------------------------GTTACGAGCAGTGATGCCT----- 19

MV_589246-1011_9_HMBSrevA11.ab1 NNNNNNNNNNNNNNNNNNCNNCTNNNNNTTNAGACCATCATCAGCAGTGTGGCGTAACTT 60

* * ******* ** *

REV_AGM_CON_SEQ_1 ------------------------------------------------------------ 19

MV_589246-1011_9_HMBSrevA11.ab1 CGATACACTTTAGCGATGTGATAATGGCAGCTGTTGATTACTTTTCTCCAGCAGACTCTT 120

REV_AGM_CON_SEQ_1 ----------------------ACCAGCTGTGGGT--CATCCTCAGGGCCATCTTCATGC 55

MV_589246-1011_9_HMBSrevA11.ab1 CTAGTACTCTGGAGTGGTGTGACTCATCTCTCAGTACCAGACTCCAGACTCCTCCAGTCA 180

** ** * ** ** *** * * *

REV_AGM_CON_SEQ_1 TGTATGCGGGAAGGAGGTGGGAATTGGTGAGAACAAATGAGATTAT-------------- 101

MV_589246-1011_9_HMBSrevA11.ab1 GGTAAGGGCGCGGCGGGTCGGGGTCCGAGGGTNNGGGCTGGCTCTCGAACCCTCCAGTCC 240

*** * * * * *** ** * * * * * *

REV_AGM_CON_SEQ_1 -----------------------------ATGCACTCNTGTTTATTACCC--------CC 124

MV_589246-1011_9_HMBSrevA11.ab1 GGTAATCNNGTAATCNNACTGCCCCATACGGGTAATNNGCCACGTGACCCCCAAACCCCC 300

* * * * * **** **

REV_AGM_CON_SEQ_1 TCACCCTCCAGCTTTGGTACCTGGGCAGGGACATGGATGGTAGCCTGCATGGTGTCTTGT 184

MV_589246-1011_9_HMBSrevA11.ab1 CCANTCTCCGAATTTGTGAGATTAGCCGACTCCN-------ACTCCGACTCCGGCTCCGG 353

** **** **** * * ** * * * * * * * *

REV_AGM_CON_SEQ_1 ATGCTATCTGAGCCATCTAGACTCCAGACTCCTCCAGT---------------------- 222

MV_589246-1011_9_HMBSrevA11.ab1 CTTTCNNATCAACCGACTCAACTCCANACTCCTCCNTCANGTAAGNGGANACTCNGNCNC 413

* * * ** ** ****** ********

REV_AGM_CON_SEQ_1 ------------------------------------------------------------ 222

MV_589246-1011_9_HMBSrevA11.ab1 NATNTNTTCTAAAGGATGGATNNNNTGNGCCAANTCCGATACNACCANGGNNTNANTTTG 473

REV_AGM_CON_SEQ_1 ----------------------------- 222

MV_589246-1011_9_HMBSrevA11.ab1 TAANATTCNTACCANACTAGACTCTATAC 502

Mosquito 10

REV_AGM_CON_SEQ_1 ------------------------------------------------------------ 0

MV_589246-1012_10_HMBSrevA12.ab1 NNNNNNNNNNNTCNNNNNGTNNTNAGTTCTACNACGNTNTGGCTNTGANNTGCTCNGGNA 60

REV_AGM_CON_SEQ_1 -------------------------GTTACGAGCAGTGATGCCT---------------- 19

MV_589246-1012_10_HMBSrevA12.ab1 CAAGAGAATGAGAGAACTATTATCAGTGTCAGCCAGTGCAGACTCCAGACTCCTCCAGTC 120

** * ***** * **

REV_AGM_CON_SEQ_1 --ACCAGCTGTGGGTCATCCTCAGGGCCATCTTCATGCTGTATGCGGGAAGGA--GGTGG 75

MV_589246-1012_10_HMBSrevA12.ab1 AGGTAATCGGTAAGTGATTCTCATCANCTTCTGCAACATGGACTGTGGGANCAAACATCC 180

* * ** ** ** **** * *** ** ** * ** * * *

REV_AGM_CON_SEQ_1 GAATT------------GGTGAGAACAAATGAGATTATA------TGCACTCNTGTTTAT 117

MV_589246-1012_10_HMBSrevA12.ab1 TCATTGGCTGGCGGAGAGGTGGAAAACGATCCACCTCTCCAAACCTCCTCTGATCTGGCT 240

*** **** ** ** * * * * ** * * *

REV_AGM_CON_SEQ_1 TACCCCCTCACCCTCCAGCTTTGGTACCTGGGCAGGGACATG---GATGGTAGCCTGCAT 174

MV_589246-1012_10_HMBSrevA12.ab1 ACAACCAGCAGCCATGGACTANACTCCAGACTCCTCCAGTCAGGTAAAGTCCNTCTCGCT 300

** ** ** ** * * * * * * ** *

REV_AGM_CON_SEQ_1 GGTGTCTTGTATGCTA-------------------------------------------T 191

MV_589246-1012_10_HMBSrevA12.ab1 GCTANACCATCTCCTGANTGAAGCTTGCAAAAGACCTCATCCANGNGGATTTTGAATCTC 360

* * * * **

REV_AGM_CON_SEQ_1 CTGAGCCATCTAGACTCCAGACTCCTCCAGT----------------------------- 222

MV_589246-1012_10_HMBSrevA12.ab1 CCCGCAGAAGGACACTCCAGACTCCTCCAGTCANGTAAGACTGTGCNTCGAAGGGGTAGG 420

* * * ******************

REV_AGM_CON_SEQ_1 --------------------------------------------------------- 222

MV_589246-1012_10_HMBSrevA12.ab1 AGGCTTCNANGAGCTCCTGGGGGGANCCGGNNACTCCAGACTCTTCCATTCAGGTAA 477

Mosquito 11

REV_AGM_CON_SEQ_1 --------------GTTACGAGCAGTGATGCCTACCAGCTGTGGGTCATCCTCAGGGCCA 46

MV_589246-1013_11_HMBSrevB01.ab1 NNNNNNNNNCNNCNNNNNNGNNCNCTTNGNGTTGNNNNCNNNTGNGNNN---CGTGGCGN 57

* * * * * * * ***

REV_AGM_CON_SEQ_1 TCTTCATGCTGTATGCGGGAAGGAGGTGGGAATTGGTGAGAACAAATGAGATTATATGCA 106

MV_589246-1013_11_HMBSrevB01.ab1 NNNTCCATCTGCTTNNNGATGTGATGTGTGGGATGTTGATTACTTTTCACANTCAAACTC 117

** *** * * ** *** * ** *** ** * * * * *

REV_AGM_CON_SEQ_1 CTCNTGTTTATTACCCCCTCACCCTCCAGCTTTGGTACCTGGGCAGGGACATGGATGGTA 166

MV_589246-1013_11_HMBSrevB01.ab1 TTCTTGTACTCTGGAGTGGTGTGACTCATCTCTCAGTACCAGACTCCAAACTCCTGNANT 177

** *** * ** ** * * * * * *

REV_AGM_CON_SEQ_1 GCCTGCATGGTGTCTTGTATGCTATCTGAGCCATCTAGACTCCAGACTCCTCCAGT---- 222

MV_589246-1013_11_HMBSrevB01.ab1 CANGTAATGGTCTCTTGTATGCTATCTGAGCCGTCTAGACTCCAGACTCCTCCAGTCAGG 237

***** ******************** ***********************

REV_AGM_CON_SEQ_1 ------------------------------------------------------------ 222

MV_589246-1013_11_HMBSrevB01.ab1 TAANCTGCGCNGATTTCGGAGTCGTCCAATCCGNNANTTCCCCAAACCTTTAGTCCNNAN 297

REV_AGM_CON_SEQ_1 ------------------------------------------------------------ 222

MV_589246-1013_11_HMBSrevB01.ab1 TCNNCCGGTNACGTCANNGAGGANACANGNCCCCACCTTATTAGANGCAAANCTCCCAGC 357

REV_AGM_CON_SEQ_1 ------------------------------------------------------------ 222

MV_589246-1013_11_HMBSrevB01.ab1 TCCNAANGAGCATGGCCAAANTAGTGAACCCCTTTNGTAACGTGAGTTCAGAACCNGGCN 417

REV_AGM_CON_SEQ_1 --------------------------------------------------- 222

MV_589246-1013_11_HMBSrevB01.ab1 NCAANNCCGNNACTNNNNACAAANNGGGGGGTTNNCCTNATANNCTGTCTN 468

Mosquito 12

REV_AGM_CON_SEQ_1 GTTACGAGCAGTGATGCCTACCAGCTGTGGGTCATCCTCAGGGCCATCTTCATGCTGTAT 60

MV_589246-1014_12_HMBSrevB02.ab1 ----NNNNNNNCGNNNNNTNCCATAACTGCANACTCCANACTCCTCCTNTCAGG---TAA 53

* * *** ** *** * * *** * **

REV_AGM_CON_SEQ_1 GCGGGAAGGAGGTGGGAATTGGTGAGAACAAATGAGATTATATGCACTCNTGTTTATTAC 120

MV_589246-1014_12_HMBSrevB02.ab1 GAG--CTGTNACNNTCGATACACTTTANTAAATGTGATAATGGCAGCTGTTGATTACTTT 111

* * * ** * ***** *** ** ** ** *** *

REV_AGM_CON_SEQ_1 CCCCTCACCCTCCAGCTTTGGTACCTGGGCAGGGACATGGATGGTAGCCTGCATGGTGTC 180

MV_589246-1014_12_HMBSrevB02.ab1 TCTCCAGCAGACT-CTTCTAGTAC-TCT--------------------------GGAGTG 143

* * * * * * **** * ** **

REV_AGM_CON_SEQ_1 TTGTATGCTATCTGAGCCATCTAGACTCCAGACTCCTCCAGT------------------ 222

MV_589246-1014_12_HMBSrevB02.ab1 GTGTGACTCATCTCTCAGTACCAGACTCCAGACTCCTCCAGTCAGGTAAACTCCNACTCC 203

*** **** * ********************

REV_AGM_CON_SEQ_1 ------------------------------------------------------------ 222

MV_589246-1014_12_HMBSrevB02.ab1 TCCAGTCANGTAANCTTCTAGACNCNNAATACGTCTAGTAGGGNAACNCCTNGCTATTAN 263

REV_AGM_CON_SEQ_1 -------------------------------------------------- 222

MV_589246-1014_12_HMBSrevB02.ab1 GNTCNNNTGNNTGGNTGGGCGGCCGGNANAGTGTNNNGGTTNNCACGTNN 313

Mosquito 13

REV_AGM_CON_SEQ_1 ---------GTTACGA--GCAGTGATGCCTACCAGCTGTGGGTCATCCTCAGGGCCATCT 49

MV_589246-1015_13_HMBSrevB03.ab1 NNNNNNNANNTNNCNTGCNCNGCTTTACTTTACAANCCTCNTGTGNGGTAANNCGTAACT 60

* * * * * * * ** * * * * **

REV_AGM_CON_SEQ_1 TCATGCTGTATGCGGGAAGGAGGTGGGAATTGGTGAGAACAAATGAGATTATATGCACTC 109

MV_589246-1015_13_HMBSrevB03.ab1 TTGATCTGTTA--GCGATGTGATGTGTGGCTGTTGATTACTTTTCACCATCTNACCCTTC 118

* **** * ** * * ** *** ** * * * * * **

REV_AGM_CON_SEQ_1 NTGTTTATTACCCCCTCACCCTCCAGCTTTGGTACCTGGGCAGGGACATGGATGGTAGCC 169

MV_589246-1015_13_HMBSrevB03.ab1 TAGTACTCTGGAGTGGTGTGACTCATCTCTCAGTACCAGACTCCAAACTCCACCAGTCAG 178

** * ** ** * * * * * * *

REV_AGM_CON_SEQ_1 TGCATGGTGTCTTGTATGCTATCTGAGCCATCTAGACTCCAGACTCCTCCAGT------- 222

MV_589246-1015_13_HMBSrevB03.ab1 GTAATGGTCTCTTGTATGCTATCTGAGCCGTCTAGACTCCAGACTCCTCCAGTCAGGTAA 238

***** ******************** ***********************

REV_AGM_CON_SEQ_1 ------------------------------------------------------------ 222

MV_589246-1015_13_HMBSrevB03.ab1 NGNNNGCAAGNTTGNNACTTCNNCGNNGGGNAAGCCCCACCCTTTCGTCTTAAGAATAGA 298

REV_AGM_CON_SEQ_1 ------------------------------------------------------------ 222

MV_589246-1015_13_HMBSrevB03.ab1 CGGGGGAAAAATCGGANGAACNGACGCCATTGCATTGGAAGCCGAACTCCCATCGCCNAC 358

REV_AGM_CON_SEQ_1 ------------------------------------------------------------ 222

MV_589246-1015_13_HMBSrevB03.ab1 AGACCTGGCCCGCCCTGTGGGGAGGCGCTGAAACGTGGGATCNGAACCTGGGCATCAGCA 418

REV_AGM_CON_SEQ_1 ----- 222

MV_589246-1015_13_HMBSrevB03.ab1 NGGNA 423

Mosquito 14

REV_AGM_CON_SEQ_1 ------------------------------------------------------------ 0

MV_589246-1016_14_HMBSrevB04.ab1 NNNNNNNNNNNNNANGNNNNNCTATACATGATTTGTTGTCNCNACTTNNTNNNCTANNNN 60

REV_AGM_CON_SEQ_1 ------------------------------------------GTTACGAGCAGTGATGCC 18

MV_589246-1016_14_HMBSrevB04.ab1 NCTGGCTCTTGAGGACCCCGATTNCGCCGCCTGNGTTCCCCCGCTAATATCTGTTGTNNG 120

* ** * * ** *

REV_AGM_CON_SEQ_1 TACCAGCTGTGGGTCAT------------------------------CCTCAGGGCCATC 48

MV_589246-1016_14_HMBSrevB04.ab1 ATCGTTATGTTGGTTTTCTGTAAGGTTCCCGGTCTGGGCTTCGGTGGGCGCCCAGACAGC 180

* *** *** * * * * ** *

REV_AGM_CON_SEQ_1 TTCATGCTGTATGCGGGAAGG---------------------------AGGTGGGAATTG 81

MV_589246-1016_14_HMBSrevB04.ab1 TTCACCATATCCACGGTANGNNAACTTTAACTGCGNACTCCTCCAGTCAGGTAANGATCA 240

**** * * *** * * **** **

REV_AGM_CON_SEQ_1 GTGAGA------------------ACAAATGAGAT---TATATGCACTCNTGT---TTAT 117

MV_589246-1016_14_HMBSrevB04.ab1 GAGAGAGACTCCNGACTCCTCCAGTCGGGTAATNTTGTGCGGTGAAGTGNTNTNTTTTCN 300

* **** * * * * ** * * ** * **

REV_AGM_CON_SEQ_1 TACCCCCTCACCCTCCAGCTTTGGTACCTGGGCAGGGACATGGATGGTAGCCTGCATGG- 176

MV_589246-1016_14_HMBSrevB04.ab1 GAAGGAGNNCNNNTCCANGCTTGTTNGCGGAGGNGTACNATGCCGCCNACCGCTCATGGG 360

* **** *** * * * * * *** * * *****

REV_AGM_CON_SEQ_1 --------------------------------------------------------TGTC 180

MV_589246-1016_14_HMBSrevB04.ab1 TGGTTGTNACNNGGNNTATNCGAAATTCTTCNGGACCGACGAAAGAATGGGCCTCCATGC 420

*

REV_AGM_CON_SEQ_1 TTGTATGCTATCTGAGCCATCTAGACTCCAGACTCCTCCAGT------------------ 222

MV_589246-1016_14_HMBSrevB04.ab1 CTCTTTGCGCCNANACAATCGGAGACTCCAGACTCCTCCAGTNNGTAANNNTGCCTCCGC 480

* * *** * ********************

REV_AGM_CON_SEQ_1 ------------------------------------------------------------ 222

MV_589246-1016_14_HMBSrevB04.ab1 GTGCCATGNTTNTNCANNNNACTGATAACTCTTCTAGNCAGGAAAGCGGCCGANGTNNCC 540

REV_AGM_CON_SEQ_1 ------------------------------------------------ 222

MV_589246-1016_14_HMBSrevB04.ab1 GTTNTTGGGNTGNCAGCCGGAAANAGTNCCNGNCTGAGGATNCGGCCA 588

Mosquito 15

REV_AGM_CON_SEQ_1 ---------------------------------------------GTTACGAGCAGTGAT 15

MV_589246-1017_15_HMBSrevB05.ab1 NNNNNNNNNNNTGNNNNNNNNAGCACTCTGGCAGCTTTTGCCCACCTTTGGGGTACGTGC 60

** * * *

REV_AGM_CON_SEQ_1 GCCTACCAGCTGTGGGTCATCCTCAGGGCCATCT---------------------TCATG 54

MV_589246-1017_15_HMBSrevB05.ab1 CCTCCCCAGATCTTACACAGCACCTGGGGCAGCAGGATGGGCTTGGGGGGGTGGCTCATG 120

* **** * * ** * * *** ** * *****

REV_AGM_CON_SEQ_1 CTGTATGCGGGAAGGAGGTGGGAATTGGTGAGA-------ACAAA--------------- 92

MV_589246-1017_15_HMBSrevB05.ab1 TCCTGTGATGGAGCACGCTCAGCACAGGGCTCAGCTCAGGGGCAGACAGACTCCAGACTC 180

* ** *** * * * * ** * *

REV_AGM_CON_SEQ_1 ------------------------------------------------------------ 92

MV_589246-1017_15_HMBSrevB05.ab1 CTCCAGTCAGGNNNNNCTGTCCCCTTTCTNCNGNTCNNTCACGTCCTNATCAACAGTTTG 240

REV_AGM_CON_SEQ_1 -------------------------TGAGATTATATGCACTC-NTGTTTATTACCCCCTC 126

MV_589246-1017_15_HMBSrevB05.ab1 CAGGCGAAACCTTAGCCCGTCGTTAACAGTTTCTANAAGCTACACATTTATTGCCAGGCA 300

** ** ** ** ****** **

REV_AGM_CON_SEQ_1 ACCCT-CCAGCTTTGG-------------------------------------------- 141

MV_589246-1017_15_HMBSrevB05.ab1 TTGCTCCAANCTTTGNNANNANANCNNCAACTATGCTCTTTAACTTCAAGGCCGCCCTTT 360

** * * *****

REV_AGM_CON_SEQ_1 ----------TACCTGGGCAGGGACATGGATGGTAGCCTGCATGGT---------GTCTT 182

MV_589246-1017_15_HMBSrevB05.ab1 ATTATCTTGTTTCCNGGGNAAGCTCCCTGANGNGACATTCCCCAGTCCTCTCACCCTTTA 420

* ** *** * * * ** * * * * ** * *

REV_AGM_CON_SEQ_1 GTATGCTATCTGAGCCATCTAGACTCCAGACTCCTCCAGT---------- 222

MV_589246-1017_15_HMBSrevB05.ab1 TCCTCCAGCATTCGAGTTCAAGACTCCANACGCCTCCGATCTGGTAAGCA 470

* * * * ** ******** ** ***** *

Mosquito 16

REV_AGM_CON_SEQ_1 ------------------------------------------------------------ 0

MV_589246-1018_16_HMBSrevB06.ab1 NNNNNNNNNGCTNCCNNNNAANCCTTNNNGTGTCTNNNANTATNCCCTCTCNATACCCAG 60

REV_AGM_CON_SEQ_1 --------------------------------------------GTTACGAGCAGTGATG 16

MV_589246-1018_16_HMBSrevB06.ab1 GGGCGCAAGGACACCAATTGTCGCCTTCTGAGCCCGCCCCGCTTGNTGCCNTTGGTGCTA 120

* * * *** *

REV_AGM_CON_SEQ_1 CCTACCAGCTGTGGGTCAT----------------------------------------- 35

MV_589246-1018_16_HMBSrevB06.ab1 TTTATCTGCTGGGGTTTTGACCGACTCCCAATCTGGGCTTCTCNGACTCCANACTCCTCC 180

** * **** ** *

REV_AGM_CON_SEQ_1 ------------------------------------------------------------ 35

MV_589246-1018_16_HMBSrevB06.ab1 NTACCAGTAAAACGGTAACAGACCTTAGACTCCAGACTCCTCCAGTCAGGTAANCTTTTT 240

REV_AGM_CON_SEQ_1 ---------CCTCAGGGCCATCTTCATGCTGTATGCGGGAAGGAGGTGGGAATTGGTGAG 86

MV_589246-1018_16_HMBSrevB06.ab1 TTTTAAGGGTCTCTCAGCCCTGGTTTTGCTCCGTTNGNCGANGGGGTTNCCGGGAGTGCC 300

*** *** * * **** * * * * *** ***

REV_AGM_CON_SEQ_1 AA-------CAAATGAGATTATATGCACTCNTGTTTATTACCCCCTCACCCTCCAGCTTT 139

MV_589246-1018_16_HMBSrevB06.ab1 TTCTANTACTCTCTCAGAATTTGAC-CCNNTNCTCTAATCCCGTCAACCCATCCTGATTC 359

* *** * * * * ** * ** * ** *** * **

REV_AGM_CON_SEQ_1 ---------GGTACCTGGGCAGGGACA--------------------------------- 157

MV_589246-1018_16_HMBSrevB06.ab1 TAAAAANTGAATACNTAGCGGAAGATANNCGGCTTCCGATTCAAATTCCNTATGTCTTTA 419

*** * * ** *

REV_AGM_CON_SEQ_1 ------------------------------------------------------------ 157

MV_589246-1018_16_HMBSrevB06.ab1 TTCCTGCCTTGCAAGTACAAATACTCTGCTGCGCCCTGNTGGTGCGTTTTCGTCCCGCCG 479

REV_AGM_CON_SEQ_1 -------------------------------TGGA--------TGGTAGCCTGCATGGTG 178

MV_589246-1018_16_HMBSrevB06.ab1 NGCCCCNCCACTGCACTTTGCTCCGATCACATGGGCGNTNNGCNCGTCGGCTGCTGCGTT 539

*** ** * **** **

REV_AGM_CON_SEQ_1 TCTTGTATGCTATC--TGAGCCAT------------CTAGACTCCAGACTCCTCCAGT-- 222

MV_589246-1018_16_HMBSrevB06.ab1 CGGTCTTTGGAGTTTCTGNCACAATGANACTTNATGATGAAGACCAGACTCNCCGAGTTG 599

* * ** * ** ** * * ******** * ***

REV_AGM_CON_SEQ_1 ------------------------------------------------------------ 222

MV_589246-1018_16_HMBSrevB06.ab1 NGTAACNNANNNNTTTNGNGATTGTTNANNTNANNNTGATTCGTGGGTTCNGACCTGGNG 659

REV_AGM_CON_SEQ_1 ----------------------------------------------------- 222

MV_589246-1018_16_HMBSrevB06.ab1 TCGTGNNTNNNNNNNACACTTCTTACTCGGAGAACCTGGGCATCANCCTGGNA 712

Mosquito 19

REV_AGM_CON_SEQ_1 GTTACGAGCAGTGATGCCTACCAGCTGTGGGTCATCCTC-------AGGGCCATCTTCAT 53

MV_589246-1021_19_HMBSrevB09.ab1 -NNNNNNNNNTNNGNGAGGNGNAGATCTGATNCGTGCTCNANNNTCTANACTTTATTCCG 59

* ** * ** * * *** * * ***

REV_AGM_CON_SEQ_1 GCTGTATGCGGGAAG----GAGGTGGGAA------------------------------- 78

MV_589246-1021_19_HMBSrevB09.ab1 AGGGTCGGAGAGGTGGCATGGGCTGCCTATGGTCACATAGCTAACAGATGGAAGGCTGAC 119

** * * * * * * ** *

REV_AGM_CON_SEQ_1 -------------TTGGTGAGAACAAATGAGATTATATGCACTCNTGTTTATTACCCCCT 125

MV_589246-1021_19_HMBSrevB09.ab1 ATTACACTCCTCACTCCTCCAGGCAAGTAAGATACTGATTCCTGTCCTCCAAAAAAAGCC 179

* * *** * **** * ** * * * *

REV_AGM_CON_SEQ_1 CACCCTCCAGCTTTGGTA-----CCTGGGCAGGGACATGGATGGTAGCCTGCATGGTGTC 180

MV_589246-1021_19_HMBSrevB09.ab1 AACTCCACAGACTCCAGACTCCTCCAGTCAGGTAAGNTTNNTGATCGCCTTCATGCTGTG 239

** * *** * * ** * * * * ** * **** **** ***

REV_AGM_CON_SEQ_1 TTGTATGC---------------------------------------------------- 188

MV_589246-1021_19_HMBSrevB09.ab1 CTCNCTTCGTGTCGAGGNNNTGCGGGGATNCGGCTGCTNNACGCGGGCCTCGGGGAGCTG 299

* * *

REV_AGM_CON_SEQ_1 ------------------------------------------------------------ 188

MV_589246-1021_19_HMBSrevB09.ab1 TCTTAGNGGNGTGCTGAGACCATGCGTGACAAACAGGTTAACAGGGGGTGATGAGTGGAA 359

REV_AGM_CON_SEQ_1 -------------------------------------------------TATCTGAGCCA 199

MV_589246-1021_19_HMBSrevB09.ab1 TCCTCACTGCACACTTTGGGGATTGCCAAGAACTCTCATATTGAGCGTGGAAGTTAAGAA 419

* * * *

REV_AGM_CON_SEQ_1 TCTAGACTCCAGACTCCTCCAGT------- 222

MV_589246-1021_19_HMBSrevB09.ab1 CACAGACTCCAGACTCCTCCAGTCAGGTAA 449

********************

Mosquito 20

REV_AGM_CON_SEQ_1 ---------------------------------------GTTACGAGCAGTGATGCCTAC 21

MV_589246-1022_20_HMBSrevB10.ab1 NNNNNNNNNNNNGNTNCGCGCGCGCTTNNCNTTAAGACCNTCATCAGCAGTGTGGTGTAA 60

* * ******* * **

REV_AGM_CON_SEQ_1 C------------------------------AGCTGTGG-GTCATCCTCAGGGCCATCTT 50

MV_589246-1022_20_HMBSrevB10.ab1 CTTCGATACACTTTAGCAATGTGATAATGGCAGCTGTTGATTACTTTTCTCCAGCAGACT 120

* ****** * * * ** ** *

REV_AGM_CON_SEQ_1 CATGCTGTATGCGGG-------------------------------------------AA 67

MV_589246-1022_20_HMBSrevB10.ab1 CTTCTAGTACTCTGGANTGGTGTGACTCATCTCTCAGTACCAGACTCCAGACTCCTCCAG 180

* * *** * ** *

REV_AGM_CON_SEQ_1 GGAGGTG------------------------------GGAATTGGTGAGAACAAATGAGA 97

MV_589246-1022_20_HMBSrevB10.ab1 TCAGGNNNGNTTTTCGGANACNNTNNNTANAGGCNTAGTGACTNTTGCCCCCCACTGTGT 240

*** * * * ** * * ** *

REV_AGM_CON_SEQ_1 TTATATGCACTCNTGTTTATTACCCCCTCACCCTCCAGCTTTGGTACCTGGGCAGGGACA 157

MV_589246-1022_20_HMBSrevB10.ab1 GTCTGTGCAGACACCNN----------ACTCCTCCAGTCNGTAAAAGATTCAAATTACCA 290

* * **** * * ** * * * * * * **

REV_AGM_CON_SEQ_1 TGGATGGTAGCCTGCATGGTGTCT---TGTATGCTATCTGAGCCATCTAGACTCCAGACT 214

MV_589246-1022_20_HMBSrevB10.ab1 ATTATGGATGGTTTTTTGTTGTGATGGACTCACTGCACATATCCGTCCAGACTCCNGACT 350

**** * * ** *** * * * ** ** ******* ****

REV_AGM_CON_SEQ_1 CCTCCAGT---------------------------------------------------- 222

MV_589246-1022_20_HMBSrevB10.ab1 CCTCCAGTCAAGTAANTNGANGTANATCATAGTTTGGAGGACGCNNGTNCNGNNGTGACC 410

********

REV_AGM_CON_SEQ_1 ---------------------------------------------------------- 222

MV_589246-1022_20_HMBSrevB10.ab1 CANNNNCCAACNNCTTCCTAATNAAATTTAGGGGGGGGGGAAAAACGNNCNNAACCTG 468

Mosquito 21

REV_AGM_CON_SEQ_1 ------------------------------------------------------------ 0

MV_589246-1023_21_HMBSrevB11.ab1 NNNNNNNNNNNNNNNNTCNNCTTANNNTNNACCACCNNNTCAGNAGTGTGACGTAACTTC 60

REV_AGM_CON_SEQ_1 ------------------------------------------------------------ 0

MV_589246-1023_21_HMBSrevB11.ab1 GATACACTTTATAAATGTGATAATGGCGGCTGTTGATTACTTTTCTCCACCAAACTCTTC 120

REV_AGM_CON_SEQ_1 --GTTACGAGCAGTGATGCCTACCAGCTGTGGGTC--ATCCTCAGGGCCATCTTCATGCT 56

MV_589246-1023_21_HMBSrevB11.ab1 TAGTACTCTGGAGTGGTGTGACTCATCTCTCAGTACCAGACTCCAGACTCCTCCAGTCAG 180

** * **** ** ** ** * ** * *** * * *

REV_AGM_CON_SEQ_1 GTATGCGGGAAGGA---------------------------------------------- 70

MV_589246-1023_21_HMBSrevB11.ab1 GTAAAGNGGGAGGGGNTCCNGTGCCTNGTGTNCCNNANGNGTCAGGTGANGANAGTNCTC 240

*** ** ***

REV_AGM_CON_SEQ_1 --------------------------------GGTGGGAATTGGTGAGAACAAATGAGAT 98

MV_589246-1023_21_HMBSrevB11.ab1 TACGCTGCCGTAGTTGGTGGCACGGGTGGCCCGGCGGGNAGTGCCGCGTAAAGGCGGGCT 300

** *** * ** * * * * * * *

REV_AGM_CON_SEQ_1 TA---------------------TATGCACTCNTGT---TTATTACCCCCTCACCCTCCA 134

MV_589246-1023_21_HMBSrevB11.ab1 TTGCCAGANAGGGANGCTCTCTCNGTACCCTNATGANGTCTNNNNCTGANTCAGANCNCA 360

* * * ** ** * * *** **

REV_AGM_CON_SEQ_1 GC----------------------------------TTTGGTACCTGGGCAGGGACATGG 160

MV_589246-1023_21_HMBSrevB11.ab1 GANNNGATNTGGGAGAAGCCCAAACTTTTCCTCCTCTNTCGTTCTTGAGGAGGCANNNNN 420

* * * ** * ** * *** *

REV_AGM_CON_SEQ_1 ATGGTAGCCTGCATGGTGTCTTGTATGCTATCTGAGCCATCTAGACTC------------ 208

MV_589246-1023_21_HMBSrevB11.ab1 TGGAT---CTGNNAAGCNNNNGNGNTGAAATCGTACGGATATCTNCCCGNAAACNNANNN 477

* * *** * ** *** * ** * * *

REV_AGM_CON_SEQ_1 CAGACTCCTCCAGT----------- 222

MV_589246-1023_21_HMBSrevB11.ab1 AAAATGCGTCTAGGCTAGAAANNAN 502

* * * ** **

Mosquito 22

REV_AGM_CON_SEQ_1 GTTACGAGCAGTGAT-----GCCTACCAGCTGTGGGTCATCCTCAGGGCCATCTTCATGC 55

MV_589246-1024_22_HMBSrevB12.ab1 -NNNNNNNNNNTNNNGCNCANTTNACATNNNAACCATCATCNGAAGTGAGACGTNNCTTC 59

* ** ***** ** * * * * *

REV_AGM_CON_SEQ_1 TGTATGCGGGAAGGAGGTGGGAATTGGTGAGAACAAATGAGATTATATGCACTCNTGTTT 115

MV_589246-1024_22_HMBSrevB12.ab1 GATACACTTT----------------------ACCGATGTGATAATGGCAGCTGTTGATT 97

** * ** *** *** ** ** ** **

REV_AGM_CON_SEQ_1 ATTACCCC-----CTCACCCTCCAGCTTTGGTACCTGGGCAGGGACATGGATGGTAGCCT 170

MV_589246-1024_22_HMBSrevB12.ab1 ACTTTTCTCCAACATACTCTTCTAGTATTCTGGAGTGGGG-------------------- 137

* * * * * ** ** ** ****

REV_AGM_CON_SEQ_1 GCATGGTGTCTTGTATGCTATCTGAGCCATCTAGACTCCAGACTCCTCCAGT-------- 222

MV_589246-1024_22_HMBSrevB12.ab1 -------------TGACTCATCTCTCAATACCAGACTCCNNACTCCTCCANNCANGTANN 184

* **** * ******* *********

REV_AGM_CON_SEQ_1 -- 222

MV_589246-1024_22_HMBSrevB12.ab1 CT 186

Mosquito 23

REV_AGM_CON_SEQ_1 -----------GTTACGAGCAGTGATGCCTACCAGCTGTGGGTCATCCTCAGGGCCATCT 49

MV_589246-1025_23_HMBSrevC01.ab1 NNNNNNNNNNNGTTTCNCGCGCNCTTTCCNTTAA---GACCATCATCAGCAGTGTGGCGT 57

*** * ** * ** * * ***** *** * *

REV_AGM_CON_SEQ_1 TCATGCTGTATGCGGGAAGGAGGTGGGAAT-------------TGGTGA---GAAC---- 89

MV_589246-1025_23_HMBSrevC01.ab1 AACTTCGATACACTTTAGCGATGTGATAATGGCAGCTGTTGATTACTTTTCTCCAGCAGA 117

* * ** * * ** *** *** * * *

REV_AGM_CON_SEQ_1 -AAATGAGATTATATGCACTCNTGTTTATTACCC-----CCTCACCCTCCAGCTT--TGG 141

MV_589246-1025_23_HMBSrevC01.ab1 CTCTTCTAGTACTCTGGAGTGGTGTGACTCATCTCTCAGTACCAGACTCCAGACTCCTCC 177

* * * ** * * *** * * * ** ****** * *

REV_AGM_CON_SEQ_1 TACCTGGGCAGGGACATGGATGGTAGCCTGCATGGTG--------TCTTGTATGC---TA 190

MV_589246-1025_23_HMBSrevC01.ab1 AGTCAGGTNNNNGGAGTGGGTNNCAGAGTGCAGAGNCNNNAAGAGTCTCCTCCNNCAACG 237

* ** * *** * ** **** * *** *

REV_AGM_CON_SEQ_1 TCTGAGCCATCTAGACTCCAGACTCCTCCAGT---------------------------- 222

MV_589246-1025_23_HMBSrevC01.ab1 TATTGCTNAGCCGTACTCACTACTCCTCCAGTGTGGTAAAGCGGAATCTGACGGATNGGT 297

* * * * **** ***********

REV_AGM_CON_SEQ_1 ------------------------------------------ 222

MV_589246-1025_23_HMBSrevC01.ab1 GATGGTTTTATGTGGTCATGGCTCCTCCATTGANATAAGTNN 339

Mosquito 24

REV_AGM_CON_SEQ_1 GTTACGAGCAGTGATGCCTACCAGCTGTGGGTCATCCTCA-------------------- 40

MV_589246-1026_24_HMBSrevC02.ab1 NNNNNNNNNNNNNCTGTNTNNTNGCNCTGTGTCCTGCTCTGTGGCTGTGGGGAGATCAGG 60

** * ** ** *** * ***

REV_AGM_CON_SEQ_1 -------------------------GGGCCATCTTCATGCTGTATGCGGGA--------- 66

MV_589246-1026_24_HMBSrevC02.ab1 AAACTGCAACAGTGTCTGCATTCGGAGGCATTTTCCATAGTCTGGAAGAGCCAGGAACTG 120

*** * * *** * * * *

REV_AGM_CON_SEQ_1 ---------------AGGAGGTGGGAATTGGTGAGAACAAATGAGATTATATGCACTCNT 111

MV_589246-1026_24_HMBSrevC02.ab1 CNCCTGTTAACTGATGGGGCTGGAGTGCTCCACAGAGTGAGTCCTTCCATCTGCAGCCCT 180

** * * * *** * * ** **** * *

REV_AGM_CON_SEQ_1 GTTTATTACCCCCT---------------------------------------------- 125

MV_589246-1026_24_HMBSrevC02.ab1 CTCTGTCAAGTCCAGCTATGGCCAGACTCCAGACTCCTCCAGTCAGGTAAANNTCTCTGN 240

* * * * **

REV_AGM_CON_SEQ_1 -CACCCTCCAGCTTTGGTACCTGGGCAGGGACATGGATGGTAG------CCTGCATGGTG 178

MV_589246-1026_24_HMBSrevC02.ab1 TCTGGNNCTTCCTTTCNATCAGGNACAGGGGTGGCTCTGCTGCCCGGCTCCCGTTTTGCA 300

* * **** * * ***** ** * ** * * *

REV_AGM_CON_SEQ_1 TCTTGTATGCTATCTGAG-----CCATCTAGA---------------------------- 205

MV_589246-1026_24_HMBSrevC02.ab1 NCTGGTGNGACATCNTACTCGTCCCTTCTGNCTTTGGCNGCCCTTCCTTTGNNTGCTNCC 360

** ** * *** * ** ***

REV_AGM_CON_SEQ_1 ------------------------------------------------------------ 205

MV_589246-1026_24_HMBSrevC02.ab1 CNCNGAGACTNCCTGACCATCCCTCACATTATGTTTCCTGTCATTCCTTGGTNNNAGCTG 420

REV_AGM_CON_SEQ_1 ------------------------------------------------------------ 205

MV_589246-1026_24_HMBSrevC02.ab1 GGCGGCACCCCGAGANGGCCCGCTTGTAGCTCCTTTNTGGCTGCTCGGAGGCGGGAGAAN 480

REV_AGM_CON_SEQ_1 ------------------------------------------------------------ 205

MV_589246-1026_24_HMBSrevC02.ab1 NNGGGNGCNTCNTGNNANNANCGGGGAATCTCNNNGGGAAANNNTCCCACNTCANTGTTC 540

REV_AGM_CON_SEQ_1 -----CTCCAGACTCCTCCAGT-------------------------------------- 222

MV_589246-1026_24_HMBSrevC02.ab1 ACANACTCNNNACTCCTCCNGGCANGTAANNNNNTNGNNANNNGNGAGANGGNNNNAANA 600

*** ******** *

Mosquito 25

REV_AGM_CON_SEQ_1 ------------------------------------------------------------ 0

MV_589246-1027_25_HMBSrevC03.ab1 NNNNNNNNNNNNNNNTNNCNTNNCANCNNACTCNGACTCCTCCNCTCTGGTAAGNCGTAA 60

REV_AGM_CON_SEQ_1 ------------------------------------------------------------ 0

MV_589246-1027_25_HMBSrevC03.ab1 GAATGATACAGATCTTAGAGTTTGGATGAGGCACNCAGGCTGTTTTACACTCCATAATCC 120

REV_AGM_CON_SEQ_1 --------GTTACGAGCAGTGATGCCTACCAGCTGTGGG--------------------- 31

MV_589246-1027_25_HMBSrevC03.ab1 TCCAGTNNGGTAANGANTGGGGTGACTCATCTCTCAGTACCAGACTCCAGACTCCTCCAG 180

* ** * * ** ** ** *

REV_AGM_CON_SEQ_1 -----TCATCCTCAGGGCCATCTTCATGCTGTATGCGGGAAGGAGGTGGGAATTGGTGAG 86

MV_589246-1027_25_HMBSrevC03.ab1 TCAGGTAAGGACCCAGCTCCGCATCATACCTTATNNCCNAGGATCTCTAGGACTNGAATG 240

* * * * * * **** * *** * * * * * * *

REV_AGM_CON_SEQ_1 AACAAATGAGATTATATGCACTCNTGTTTATTACCCCCTCACCCTCCAGCTTTGGTACCT 146

MV_589246-1027_25_HMBSrevC03.ab1 CTTTGCN-TATTCCAATATAAGTGTGANNNATGCCCCTGCTCC-CCGCGCCGTGGTTAAT 298

* ** * ** * **** * ** * ** **** *

REV_AGM_CON_SEQ_1 G----------------------------------------------------------- 147

MV_589246-1027_25_HMBSrevC03.ab1 GCATGCGGTGCNAGATCGGATGCTTCCTCTGNGCATTAACTGAGTTCTCCNTTTGAGTGA 358

*

REV_AGM_CON_SEQ_1 -------------------------------------------------------GGCAG 152

MV_589246-1027_25_HMBSrevC03.ab1 CTTTCACTGAGAGAGTCAGTGATAAGCCCAAACNNTACCTTTCCTGTCTTTCTTGAGGAG 418

* **

REV_AGM_CON_SEQ_1 GGACATGGATGGTAGCCTGCATGGTGTCTTGTAT--------GCTATCTGA----GCCAT 200

MV_589246-1027_25_HMBSrevC03.ab1 GNATTGCNATGGAAGCGGGAAGGGTCGCGCGTCATTCGCTCGGATGGCTGCNCGNCGGNG 478

* * **** *** * * *** * ** * * ***

REV_AGM_CON_SEQ_1 CTAGACTCCAGACTCCTCCAGT------- 222

MV_589246-1027_25_HMBSrevC03.ab1 GNANANNNNANGCTTCTTGNCNGGAAANN 507

* * * ** **

Mosquito 27

REV_AGM_CON_SEQ_1 ----------------------GTTAC----GAGCAGTGATGCCTACCAGCTGTGGGT-- 32

MV_589246-1029_27_HMBSrevC05.ab1 NNNNNNNNNNNNNNNATNCTCNGNNACNATNANGAGNTNTTTCNNAGAGTCTGAGGNTGA 60

* ** * * * * * *** ** *

REV_AGM_CON_SEQ_1 -------------CATCCTCAGGGCCATCTTCATG---------------CTGTATGCGG 64

MV_589246-1029_27_HMBSrevC05.ab1 TCCCGTCTAAATTGATCATCCTGGACATCTTTGATGAATGTGTCGCACTTCTNGGACCNN 120

*** ** ** ****** ** *

REV_AGM_CON_SEQ_1 GAAGGAGGTGGGAATTGGTGAGAACAAATGAGATTATATGCAC----------------- 107

MV_589246-1029_27_HMBSrevC05.ab1 CCATGGCCTTGAGATTGATGAGGAAANACCGGATCGACTGGTTGCCAACCTGTTCGCATT 180

* * * * **** **** * * * *** **

REV_AGM_CON_SEQ_1 ------------------------------------------------------------ 107

MV_589246-1029_27_HMBSrevC05.ab1 TCCTAAACTTATACCTCTCCTCCAGGACTGTAGAATCTGGANGAAGGTTNNNCTTGANNC 240

REV_AGM_CON_SEQ_1 ---------------------TCNTGTTTATTACCCCCTCACCCTCCAGCTTTGGTACCT 146

MV_589246-1029_27_HMBSrevC05.ab1 NACTGAGGACATCTTGTAACGTTATACTACATGGANCAACAAGCGCGCANGNTTATACCG 300

* * * * * ** * * * ****

REV_AGM_CON_SEQ_1 GGGCAGGGACATGGATGGTAGCC------------------------------------- 169

MV_589246-1029_27_HMBSrevC05.ab1 CGAAAGCGAAAATCCTTGAGACTCCCNACTCCTCCNNTAAGGTAATCNACCCGACTCTTC 360

* ** ** * * * *

REV_AGM_CON_SEQ_1 ------------------------------------------------------------ 169

MV_589246-1029_27_HMBSrevC05.ab1 NCGGTGGNGCTCCCTCAAAAAATGCGCCATCCCAGTCGACTTTCTGCTGCGACCACTTCC 420

REV_AGM_CON_SEQ_1 ------------------------------------------------------------ 169

MV_589246-1029_27_HMBSrevC05.ab1 CTTGTGCTCCTCTCTCATGTTTTGAAGCGGCTGACCACCTCACTGGCTACTACGGCGATT 480

REV_AGM_CON_SEQ_1 ------------------------------------------------------------ 169

MV_589246-1029_27_HMBSrevC05.ab1 ATTAATTGGGGGGTTATGCCGANGGACCTCTTNCGGCGCTACTTCCTCNNGTGCGGATGA 540

REV_AGM_CON_SEQ_1 ------------------------------------------------------------ 169

MV_589246-1029_27_HMBSrevC05.ab1 AGATAACGANNNTCGNCATTCACCACNNNGTGCTGGNGTAGTTTTANNNGNNCCATCGGA 600

REV_AGM_CON_SEQ_1 -----------------------------TGCATGGTGTCTTGTATGCTATCTGAGCCAT 200

MV_589246-1029_27_HMBSrevC05.ab1 NTGANTCTCAGNAGANAGCTANTGTTCTNANTNCGGACTNNNNTCTTNAATTNGAANNGT 660

** * * * ** ** *

REV_AGM_CON_SEQ_1 CTAGACTCCAGACTCCTCCAGT------- 222

MV_589246-1029_27_HMBSrevC05.ab1 TGANACTCCANACTCCTCCANNCNNNNAA 689

* ****** *********

Mosquito 28

REV_AGM_CON_SEQ_1 ------------------------------------------------------------ 0

MV_589246-1030_28_HMBSrevC06.ab1 NNNNNNNNNNNNATNNCGACTCCGACTTTGACTCCGACTTCGACTTCGACTTCACTTCGT 60

REV_AGM_CON_SEQ_1 --GTTAC---------------------GAGCAGTGATGCCTACCAGCTGTGGGT-CATC 36

MV_589246-1030_28_HMBSrevC06.ab1 CGATTTCGACTTCTTCGACTTCGACTCNGACTCCNCACTCCTCCAGTCAGGTAATAANGN 120

** * ** * *** * * * *

REV_AGM_CON_SEQ_1 CTCAGGGCCATCTTCATGCTGTATGCGGGAAGG--------------------------- 69

MV_589246-1030_28_HMBSrevC06.ab1 TTCATCTNNNTNTTTTTNNAGTTTNCCGGCCCGGGTTGAGCGCAGCACTTTGCGCTCATN 180

*** * ** * ** * * ** *

REV_AGM_CON_SEQ_1 AGGTGGGAATTGGTGAGAACAAATG----AGATTATATGCACTCNTGTTTAT-------- 117

MV_589246-1030_28_HMBSrevC06.ab1 GAGTCNCCAATCCTCAAAGCAGGTAAAGGCCTGCATGTGNNNNNGGNTCTGGNNCCGGCA 240

** * * * * * ** * ** ** * *

REV_AGM_CON_SEQ_1 ----------TACCCCCTCACCCTCCAGCTTTGGTACCTGGGCAGGGACATGGATGGTAG 167

MV_589246-1030_28_HMBSrevC06.ab1 CTGGNTTCGACTCTGAATCAGACTCCACGTGTGATAGTGGGACTGGCTCATNT------- 293

* *** ***** * ** ** ** * ** ***

REV_AGM_CON_SEQ_1 CCTGCATGGTGTCTTGTATGCTATCTGAGCCATCTAGACTCCAGACTCCTCCAGT----- 222

MV_589246-1030_28_HMBSrevC06.ab1 TCTGACTCTGGCNCTG---------GATCCGACTCGGACTCNNNNNTCNTCNAGTCAGGT 344

*** * * ** * * ***** ** ** ***

REV_AGM_CON_SEQ_1 -------------------------------------------- 222

MV_589246-1030_28_HMBSrevC06.ab1 AAACTTGGACTCCTACTTCNAATTCGANNCACACNTCGACTCNG 388

Mosquito 29

REV_AGM_CON_SEQ_1 ---------------------------------------------GTTACG--------- 6

MV_589246-1031_29_HMBSrevC07.ab1 NNNNNNNNNNNNNNNNNNNTACNNNNNCNNTTGGGNNCNNNNNNGGTTANNNCCCTTNTT 60

****

REV_AGM_CON_SEQ_1 ------------------------------------------------------------ 6

MV_589246-1031_29_HMBSrevC07.ab1 GTTCCCNCTTCTTGGGACGGGCGGTTACCTCGGATTTNACTGACTTCTCTCTCNGGGNNA 120

REV_AGM_CON_SEQ_1 ---------------------------------------AGCAGT--------------- 12

MV_589246-1031_29_HMBSrevC07.ab1 GNANACCTCCNTCGGCCNAACTTGAATACTCCAGACTCCTGCAGTCAGGTAAATACAGGG 180

*****

REV_AGM_CON_SEQ_1 ------------------------------------------------GATGCCTACCAG 24

MV_589246-1031_29_HMBSrevC07.ab1 TTCTCGTGGCAGCCCCNCGGCGCGAGAGGCACTTGGGGGCGCACTGTGGATGTCNNNAAC 240

**** * *

REV_AGM_CON_SEQ_1 CTGTGGGTC-----------------ATCCTCAGGGCCATCTTCATGCTGTATGCGGGAA 67

MV_589246-1031_29_HMBSrevC07.ab1 CTTGTNATCATNTTTNNTATACATCCNNCNTNNAAANGATCGNTTTGGTTTACCCAGAAA 300

** ** * * *** ** * ** * * **

REV_AGM_CON_SEQ_1 GGAGGTGGGAAT---------TGGTGAGAACAAATGAGATTATATGCACTCNTGTTTATT 118

MV_589246-1031_29_HMBSrevC07.ab1 GAATATGGNGGAGAAAGGACANNTCCNNNAATGNAGACAATCTTTCTAATCCTCTTAATG 360

* * *** * ** * * * * * ** * ** **

REV_AGM_CON_SEQ_1 ACCCCCTCACCCTCCAGCTTTGGTA----CCTGGGCAGGGACATGGATGGTAGCCTGCAT 174

MV_589246-1031_29_HMBSrevC07.ab1 ATTACATCACCNGGCCTCTATTCGAGGTCCTTGTTTATTTACATAATGTTTTAAACNNGG 420

* * ***** * ** * * * ** * **** *

REV_AGM_CON_SEQ_1 GGTGTCTTGT--------------------------ATGCTATCTG-------------- 194

MV_589246-1031_29_HMBSrevC07.ab1 GAAGTAATGACTCATGCCGGACTGTACGCGAATCCTTTGTAATCTTGTCCATGTTTGCGA 480

* ** ** ** ****

REV_AGM_CON_SEQ_1 ---AGCCATCTAGACTCCAGACTCCTCCAGT----------------------------- 222

MV_589246-1031_29_HMBSrevC07.ab1 AGAAGTTCCNTAGCCTTCAGACTCCTCGCGTTTAGCTCCTNCTTTNTNCTCTTCAGGACA 540

** *** ** ********** **

REV_AGM_CON_SEQ_1 ------------------------------------------------------------ 222

MV_589246-1031_29_HMBSrevC07.ab1 ACNGTTTTCCCAAAATGATGTCCAAGATCAANTACGCGNNACTNNNNNGGTTCNGNANNN 600

REV_AGM_CON_SEQ_1 ------------------------------------------------------------ 222

MV_589246-1031_29_HMBSrevC07.ab1 CTGATCNNNNGGGATTGCNCTCNNTNNCNCNTCNCNTNANNCNNANACNGNGNNNNTCNN 660

REV_AGM_CON_SEQ_1 --------- 222

MV_589246-1031_29_HMBSrevC07.ab1 NNNNANNNN 669

Mosquito 31

REV_AGM_CON_SEQ_1 -------------------------------------------------GTTACGAGCAG 11

MV_589246-1033_31_HMBSrevC09.ab1 NNNNNNNNNNNNGNTGNANNNNNNNAGCACTCTGGCAGCTTTTGCCCACCTTTGGGGTAC 60

** * * *

REV_AGM_CON_SEQ_1 TGATGCCTACCAGCTGTGGGTCATCCTCAGGGCCATCTTCATGCTGT------------- 58

MV_589246-1033_31_HMBSrevC09.ab1 GTGCCCTCCCCAGATCTTACACAGCACCTGGGGCAGCAGGATGGGCTTGGGGGGGTGGCT 120

* **** * * ** * * *** ** * *** *

REV_AGM_CON_SEQ_1 --------ATGCGGGAAGGAGGTGGGAATTGGTGAGAACAAATGAGATTATATGCACTCN 110

MV_589246-1033_31_HMBSrevC09.ab1 CGTGTCCTGTGATGGAGCACGCTCAGCACGGGGCTCAGCTCAGGGGCAGACA--GACTCC 178

** *** * * * * ** * * * * * * * ****

REV_AGM_CON_SEQ_1 TGTTTATTACCC---CCTCACCCTCCAGCTTTGGTAC--------------CTGGGCAGG 153

MV_589246-1033_31_HMBSrevC09.ab1 AGACTCCTCCAGTCAGGTAANNNTNATTATTTTNTNCNNATNGAAAACAAAATGANAAAA 238

* * * * * * * *** * * ** *

REV_AGM_CON_SEQ_1 GACATGGATGGTAGCCTGCATGGTGTCTTGTATGCTAT----------------CTGAGC 197

MV_589246-1033_31_HMBSrevC09.ab1 CATATAAACAAAAAACTATNAAAAANCTTAANTGCTTTAGGTCACCTACAAATTCAAAGN 298

* ** * * ** *** **** * * **

REV_AGM_CON_SEQ_1 CA--------------------TCTAGACTCCAGACTCCTCCAGT--------------- 222

MV_589246-1033_31_HMBSrevC09.ab1 CNNGNAAACCAGAAAANAATCCCTTNAACTCCCNACTCCTCCTCTCGAGTAANNGGTCAC 358

* * ***** ******** *

REV_AGM_CON_SEQ_1 ------------------------------------- 222

MV_589246-1033_31_HMBSrevC09.ab1 CCTTTATTATCTAGTTTCCTGAGNAGGCTTCCNTACN 395

Mosquito 33

REV_AGM_CON_SEQ_1 --------------------------------------------------GTTACGAGCA 10

MV_589246-1035_33_HMBSrevC11.ab1 NNNNNNNNNCNNNNNNNNCAGAGTTNNNCTCCGACTNCATTGGNNNAGTTGCTCCCTACA 60

* * * **

REV_AGM_CON_SEQ_1 GTGATGCCTACCAGCTGTGGGTCATCCTCAGGG-------CCATCTTCATGCTGTATGCG 63

MV_589246-1035_33_HMBSrevC11.ab1 GTCAATAATCNNAGATCNTACTCGACAATCTNGANTTATGGCTCTNNACTCCAGAGTCCC 120

** * * ** * ** * * * * * * * *

REV_AGM_CON_SEQ_1 GGAAGGAGGTGGGAATTGGTGAGAACAAATGA--------------GATTATATGCA--- 106

MV_589246-1035_33_HMBSrevC11.ab1 CCCATGGCCTACNGATTGGTGAGAAAGTAGAGGTTCGGCTGGNTGTCAAGCTGTGCACAT 180

* * * *********** * * * ****

REV_AGM_CON_SEQ_1 ---CTCNTGTTTATTACCCCCTCACCCTCCAGCTTTGGTACCT----------------- 146

MV_589246-1035_33_HMBSrevC11.ab1 TTCCGCAACCTAATCACCCCCTCCAGTACGGGAGATTCTACAAGGAAGGTNACTGTNTCT 240

* * * ** ******** * * * ***

REV_AGM_CON_SEQ_1 ------GGGCAGGGACATGGATGGTAGCCTGCATG------------------------- 175

MV_589246-1035_33_HMBSrevC11.ab1 CCAAGTGAGACCACACATTTATGCTATCCTGCAAGAAGGNACTAGAGCGCAGAGTTTTAT 300

* * **** *** ** ****** *

REV_AGM_CON_SEQ_1 ------------------------------------------------------------ 175

MV_589246-1035_33_HMBSrevC11.ab1 CGCGACTGCGAAAATCCTTGAGACTCTCNACTCCTCCNTTCAGGTAATCNACNATACTCT 360

REV_AGM_CON_SEQ_1 ------------------------------------------------------------ 175

MV_589246-1035_33_HMBSrevC11.ab1 TCTCGGCGGCGCTCCGTCGTTAAATGCGCCATCCCAGTGGAGTTTTTGCCNGGACCACTT 420

REV_AGM_CON_SEQ_1 ------------------------------------------------------------ 175

MV_589246-1035_33_HMBSrevC11.ab1 CCCCNNNNCTACTCTCTCGGCTTTTGAGCGTGCTGATCACCTTAGCGTTNTCTACTCCGA 480

REV_AGM_CON_SEQ_1 ------------------------------------------------------------ 175

MV_589246-1035_33_HMBSrevC11.ab1 TTATTAATTGNGGGGTTACATCGGTATANCTCCTCAGGGGCAATTTCTTCANGTGCGGAT 540

REV_AGM_CON_SEQ_1 ------------------------------------------------------------ 175

MV_589246-1035_33_HMBSrevC11.ab1 GAAGATCACAAAGCTCGTCATAAACCACGGTTTGCTTGNGTAGTTTTAACTGCTCTACGG 600

REV_AGM_CON_SEQ_1 -------------------------------------GTGTCTTGTATGCTATCTGAGCC 198

MV_589246-1035_33_HMBSrevC11.ab1 TCCTGNCTCTCAACGGAAAGCTCCTGTTCTCGTTACGGCCTGCAATCNTNNNNNCNAAGC 660

* * * * *

REV_AGM_CON_SEQ_1 ATCTAGACTCCAGACTCCTCCAGT------- 222

MV_589246-1035_33_HMBSrevC11.ab1 GTTGAGACTCCAGACTCCTCCAGTCAGGTAA 691

* ********************

Mosquito 34

REV_AGM_CON_SEQ_1 ------------------------------------------------------------ 0

MV_589246-1036_34_HMBSrevC12.ab1 NNNNNNNNNNCTNAGNNNNNNTAACTGCNNANNCCANACTCCTCCAGTCAGGTAANANTC 60

REV_AGM_CON_SEQ_1 ------------------------------GTTACGAG-----CAGTGATGCCTACCAGC 25

MV_589246-1036_34_HMBSrevC12.ab1 CANTACTCCTNNAGTCAGAGTAANGCATGGGTCACGGGGTGGCTGGGCCTGCCCACTACC 120

** *** * * **** ** * *

REV_AGM_CON_SEQ_1 TGTGGGTCATC----------------------CTCAGGGCCATCTTCATGCTGTATGCG 63

MV_589246-1036_34_HMBSrevC12.ab1 TACAGGCCCCACCCCACTCCACCAGTGCAGGGAGCCAGGACCAGGTGCAGACAGGCTTTG 180

* ** * **** *** * ** * * * *

REV_AGM_CON_SEQ_1 GGAAGGA-----GGTGGGA-ATTGGTGAGAA--------------------------CAA 91

MV_589246-1036_34_HMBSrevC12.ab1 GCAGGCCCCAGCGGTGGGATCATAGGCAGCCCCAAGAGGCCCAGACTCCAGACTCCTCCA 240

* * * ******* * * ** * *

REV_AGM_CON_SEQ_1 ATGAGATTATATGCACTCNTGTTTAT-TACCCCCTCACCCTCC----------------A 134

MV_589246-1036_34_HMBSrevC12.ab1 GTCAGGTAACCNTCGGACCTCCTNACTCCTCCACTCACGCCACCAGTCAGGTAACAGCCA 300

* ** * * * * * * * ** ***** * * *

REV_AGM_CON_SEQ_1 GCTTTGGTACCTGGGCAGGGACATGGATGGTAGCCTGCA--------------------- 173

MV_589246-1036_34_HMBSrevC12.ab1 GTTTCAGATNCTGTTCCGAGACTTGCCNGGTTCNNTGGCCAGGGCTGTAACCCCGACCGG 360

* ** * *** * * *** ** *** **

REV_AGM_CON_SEQ_1 -------------------TGGTGTCTTG------------------------------- 183

MV_589246-1036_34_HMBSrevC12.ab1 CCTNNCCGTGTCGCTTTANCGGTGCCTCCGANAACCTCGCCTGATTGCGGGNCAATGCCA 420

**** **

REV_AGM_CON_SEQ_1 -----------------------------------------------------TATGCTA 190

MV_589246-1036_34_HMBSrevC12.ab1 GCATACTCCCGCNNCGCTAACAGCCACAGGCGGGGGGGGGGGATNANNGGGGGTATCAGG 480

***

REV_AGM_CON_SEQ_1 TCTGAGCCATCTAGACTCCAGACTCCTCCAGT---------------------------- 222

MV_589246-1036_34_HMBSrevC12.ab1 TAGAGGGGGNCTTGNCACGGGCCGTCTACTGAAAATGGGGGANGGGAGNAACAANACCAG 540

* * ** * * * * * ** * *

REV_AGM_CON_SEQ_1 --------------- 222

MV_589246-1036_34_HMBSrevC12.ab1 CACTGGANANCGTCT 555

Mosquito 35

REV_AGM_CON_SEQ_1 ------------------------------------------------------------ 0

MV_589246-1037_35_HMBSrevD01.ab1 NNNNNNNNNGNAGNTNNCNTGCNCAGCTTNNNNNNNNANACCANCATCAGCAGTANNGGC 60

REV_AGM_CON_SEQ_1 --------------GTTACGAGCAGTGATGCCTACCAGCTGTGGGTCATCCTCAGGGCCA 46

MV_589246-1037_35_HMBSrevD01.ab1 GTAACTTCGATACACTTTAGCGATGTGATAATGGCAGCTGTTGATTACTTTTCTCCAACA 120

** * * ***** * ** * * ** **

REV_AGM_CON_SEQ_1 TCTTCATGCTGTATGCGG------------------------------------------ 64

MV_589246-1037_35_HMBSrevD01.ab1 GACTCTTCTAGTACTCTGGAGTGGTGTGACTCATCTCTCAGTACCAGACTCCAGACTCCT 180

** * *** * *

REV_AGM_CON_SEQ_1 ------------------------------GAAGGAGGTGGGAATTGGTGAGAACAAATG 94

MV_589246-1037_35_HMBSrevD01.ab1 CCAGTCAGGTAAACTCCAGACTCCTCCAGTCAGGTAAGTNGAGANGGNTCTGGTACNGAA 240

* * * ** * * * * *

REV_AGM_CON_SEQ_1 AGATTATATGCACTCNTGTTTATTACCCCCTCACCCTCCAGCTTTGGTACCTGGGCAGGG 154

MV_589246-1037_35_HMBSrevD01.ab1 AGTTTGGCTTTGCTGTAGTCGTNGCACCACTNGACAGGTAAAGCGGANNCTTCNCNGAAG 300

** ** * ** ** ** ** * * * * * *

REV_AGM_CON_SEQ_1 ACATGGATGGTAGCCTGCATGGTGTCTTGTATGCTATCTGAGCCATC------------- 201

MV_589246-1037_35_HMBSrevD01.ab1 AGATGGTTTTTTGTGGACATGGACGCAGTGANGATATCAGTCATGACTCNNTCNCCCCCC 360

* **** * * * ***** * * * **** * *

REV_AGM_CON_SEQ_1 ----------------TAGACTCCAGACTCCTCCAGT----------- 222

MV_589246-1037_35_HMBSrevD01.ab1 AGTCAAGAAACCGACTCANACNAAANACTCCTCGANTCNNGTAANGAN 408

* ** * ******* * *

Mosquito 36

REV_AGM_CON_SEQ_1 -----------GTTACGAGCAGTGATGCCTACCAGC------------------------ 25

MV_589246-1038_36_HMBSrevD02.ab1 CNNNNCNCNNNNTACCNTAANCTGCTNACTCCNAACTCCTCCACTCAGGTAAGGTGTAAC 60

* * ** * ** * * *

REV_AGM_CON_SEQ_1 -----------------------TGTGGGTCATCC----------TCAGGGCCATCTTCA 52

MV_589246-1038_36_HMBSrevD02.ab1 TTCGATTTCTTTAANAAAGTTGTTATGGGAGCTGTTGATTTCTTTTTTCCAACATACTCT 120

* **** * * *** **

REV_AGM_CON_SEQ_1 TGCTGTATGCGGGAAGGAGGTGGGAATTGG------------------------------ 82

MV_589246-1038_36_HMBSrevD02.ab1 TCTACTACTCTGGAATGGGGTGACTCATCTCTCAATTCCCGAATCCCNAATCCTCCCGTC 180

* ** * **** * **** *

REV_AGM_CON_SEQ_1 -------------------------TGAGAACAAATGAGATTATATGCACTCNTGTTTAT 117

MV_589246-1038_36_HMBSrevD02.ab1 AGGGAAANTCCAGACTCCTCCNTCAGGTAANTTANNCNGAGTATCGGACCGGATAGTTTC 240

* * * ** *** * * * **

REV_AGM_CON_SEQ_1 TACCCCCTCACCCTCCAGCTTTGGTACCTGGGCAGGGACATGGATGGTAG-CCTGCATGG 176

MV_589246-1038_36_HMBSrevD02.ab1 GATATCCTGATGGCNTGGCTGCGGTGNCGGGGCANNTTTNNNGTTACTCCAACTGGGTGG 300

* *** * *** *** * ***** * * * *** ***

REV_AGM_CON_SEQ_1 TGTCTT---------------------------------------------GTATGCTAT 191

MV_589246-1038_36_HMBSrevD02.ab1 TTTTTTGTGGCTTTGGGGGANCTGAAGACACCCNTNNTGACTCCGGCTNCGGCTTTCNNA 360

* * ** * * *

REV_AGM_CON_SEQ_1 CTGAGCCATCTAGACTCCAGACTCCTCCAGT--------- 222

MV_589246-1038_36_HMBSrevD02.ab1 GAAANCGACTCANACTCCANACTCCTCCAGTCAGGTAANN 400

* * * * ****** ***********

Mosquito 37

REV_AGM_CON_SEQ_1 ------------------------------------------------------------ 0

MV_589246-1039_37_HMBSrevD03.ab1 NNNNNNNNNNNNNNNNNNNNNNTAGNTGGTTNTCCGTCCCCNGTACCTCCTCTATGACCN 60

REV_AGM_CON_SEQ_1 ------------------------------------------------------------ 0

MV_589246-1039_37_HMBSrevD03.ab1 TGGGACTCTTTGACTCCTAAANAGGCCTTGGGNGCCCGACNNTCATCTGCCGTTGGTCGG 120

REV_AGM_CON_SEQ_1 --------------GTTACGAGCAG------------------------------TGA-- 14

MV_589246-1039_37_HMBSrevD03.ab1 ATCCACATTCTGGGGTTTTGACCGGTTCCCCATCTGGGCTTCGGTGTCCGGCAAGAGAGC 180

*** ** * * **

REV_AGM_CON_SEQ_1 ---TGCCTACCAGCTGTGGGTCATCCTCAGGGCCATCTTCATGCTGTATGCGGGAAGGAG 71

MV_589246-1039_37_HMBSrevD03.ab1 CGTACCATAGAAACGGTAACAGACCTTAGACTCCAGACTCCTCCAGTCAGGTAAATGGGT 240

* ** * * ** * * * *** ** * * ** * * **

REV_AGM_CON_SEQ_1 GTGGG---------------AATTGGTGAGAACAAATGAGATTATATGCACTC---NTGT 113

MV_589246-1039_37_HMBSrevD03.ab1 TTCCGGACTCCGTCTCTCAGTTTTTCTTGGAGCCTTTTTGGGTCTATACCTGTTTTCCCT 300

* * ** * ** * * * * *** * *

REV_AGM_CON_SEQ_1 TTATTACCCCCTCACCCTCCAGCTTTGGTACCTGGGCAGGGACATGGATGGTAGC----- 168

MV_589246-1039_37_HMBSrevD03.ab1 TGGCTTCCACTACTCCCTCNAAGTNTGACACCCAACCTCCAATCTCGAGCCCACCTTTAG 360

* * ** * * ***** * * ** *** * * * ** * *

REV_AGM_CON_SEQ_1 ----CTGCATGGTGTCTTGTATGCTATCTGAG------CCATCTAGACTCCAGACTCCTC 218

MV_589246-1039_37_HMBSrevD03.ab1 CGTGTGTACTNGTACCTCCTGTNGTGTCTGTGGGTTTCCGTCTTTAACTCCACACTCCTC 420

* ** ** * * * **** * * * ****** *******

REV_AGM_CON_SEQ_1 CAGT-------------------------------------------------------- 222

MV_589246-1039_37_HMBSrevD03.ab1 CAATCACGGGANNTCCTGANTCCTCCGCATTGNNTCAAGCTGGGATAATAGTCTTGTTCG 480

** *

REV_AGM_CON_SEQ_1 ------------------------------------------------------------ 222

MV_589246-1039_37_HMBSrevD03.ab1 GATGTCTGCGTCCTTCGACGATTGGAGCCCACTGGNGAAAATAAGGCTTTTTAATTGATC 540

REV_AGM_CON_SEQ_1 ------------------------------------------------------------ 222

MV_589246-1039_37_HMBSrevD03.ab1 CACCGTGATCTTGCTGAGCTCNANGTCTTCACAGCATCGGTTGGCAGTCCCNNCGAACGT 600

REV_AGM_CON_SEQ_1 ------------------------------------------------------------ 222

MV_589246-1039_37_HMBSrevD03.ab1 TAANGAATCGTCTGCGTCATTCTTCGTGAGCTGGAAGCACTGTTACCGTTTGCTGAGCAG 660

REV_AGM_CON_SEQ_1 ------------------------------------------------------------ 222

MV_589246-1039_37_HMBSrevD03.ab1 GGAGATTCNNNNNNNNAATAGCTCTTTCAGCTTCTTCACCATTTCATCGAACGAGTACTC 720

REV_AGM_CON_SEQ_1 ------------------------------------------------------------ 222

MV_589246-1039_37_HMBSrevD03.ab1 ACGATGGTGCTTCGGGAGCTGAAGTTGACGTACTTGTCGTGCACCGTCACNCTGANGCTT 780

REV_AGM_CON_SEQ_1 ------------------------------------------------------------ 222

MV_589246-1039_37_HMBSrevD03.ab1 CTTAACCGCACTTTTTNGCGCATCGTCCAACTTCGCTCCGTCCTGGAGGAAGAGGTCTTC 840

REV_AGM_CON_SEQ_1 ------------------------------------------------------------ 222

MV_589246-1039_37_HMBSrevD03.ab1 GTACNTTCGGNACCACCGGGTCGAAAACGANCCCGNTGTCTGGATCGTNAGACGAATTCC 900

REV_AGM_CON_SEQ_1 ----------------------------------------------- 222

MV_589246-1039_37_HMBSrevD03.ab1 CGGATGTTGGNNGCANAGACTCCAGACNTCCTCCNNTCAGGTAANNN 947

Mosquito 38

REV_AGM_CON_SEQ_1 ---------------------GTTACGAGCAGTGATGCCTACCAGCTGTGGGTCATCCTC 39

MV_589246-1040_38_HMBSrevD04.ab1 NNNNNNNNNNNNNNNNNNNNCTTTNNNNTTANACCNTCATCAGCGGTGTGGTGTAACTTC 60

** * * * * ***** * * **

REV_AGM_CON_SEQ_1 AGGGCCATCTTCATGCTGTATGCGGGAAGGAGGTGGGAATTGGTGAGAA-----CAAATG 94

MV_589246-1040_38_HMBSrevD04.ab1 GATNCNCTTTANAAAAGTCATAATGCCAGCTGTTGATTACTTTTCTCCAGCACACTCTTC 120

* * * * ** * ** * ** * * * * * *

REV_AGM_CON_SEQ_1 AGATTATATGCACTCNTGTTTATTACCC-----CCTCACCCTCCAGCTTTGG--TACCTG 147

MV_589246-1040_38_HMBSrevD04.ab1 TAGTACTCTGGANTGNTGTGACTCATCTCTCAGTACCAGACTCCAGACTCCTCCAGTCAG 180

* * ** * * **** * * * ** ****** * * *

REV_AGM_CON_SEQ_1 GGCAGGGACATGGATGGTAGCCTGCATGGTGTCTTGTATGCTATCTGAGCCATCTAGACT 207

MV_589246-1040_38_HMBSrevD04.ab1 GTAANNNNNTCGGGTAACCNGTTGCTGAGGGTACTAGAGGAGTCNGGNGNCNTAAAGTAT 240

* * ** * *** * ** * * * * * * * ** *

REV_AGM_CON_SEQ_1 CCAGACTCCTCCAGT--------------------------------------------- 222

MV_589246-1040_38_HMBSrevD04.ab1 GTGAAATGCTGTAGATGTTGATTTTTCTGGGNGGGTAANGAGGNAAATTANGAAATATGG 300

* * ** **

REV_AGM_CON_SEQ_1 ---------------- 222

MV_589246-1040_38_HMBSrevD04.ab1 NTNGTTTTATGTTNNN 316

Mosquito 39

REV_AGM_CON_SEQ_1 ---------------------GTTACGAGCAGTGATG----------------------- 16

MV_589246-1041_39_HMBSrevD05.ab1 NNNNNNNNNNNNNNNNNTTTTNGTTCTNCNAGTTATGGCTATNAACTGATCTGGGACATN 60

* * *** ***

REV_AGM_CON_SEQ_1 ------------------------------------------------------------ 16

MV_589246-1041_39_HMBSrevD05.ab1 AGAATGAGACAACTATTATTTTGNCNGCCTCTGTCGACGCCAAACTCCTCGANTCATGCA 120

REV_AGM_CON_SEQ_1 ---------------CCTACCAGCTGTGGGTCATCCTCAGGGC-------CATC------ 48

MV_589246-1041_39_HMBSrevD05.ab1 ANCCTTAATGTGATTCTCATCAGCTTCTGGGCATGAGCTGTGGGACCAAACATCCTCATT 180

* * ***** ** *** * * * ****

REV_AGM_CON_SEQ_1 ------------------------------------------------------------ 48

MV_589246-1041_39_HMBSrevD05.ab1 GGCTGGTGGAGAGGTGGAGAACGATCCACCTCTCCCAACCTCCTCTGATCTGGNNACNAC 240

REV_AGM_CON_SEQ_1 -----------------------------------------------TTCATGCTGTATG 61

MV_589246-1041_39_HMBSrevD05.ab1 CAGCAGCCATGGACTAGACTCCAGACTCCTCCAGTCAGGTAATGGACTTCAAGCTGCTTT 300

**** **** *

REV_AGM_CON_SEQ_1 CGGGAAGGAGGTGGGAATTGGTGAGAACAAATGAGATTATATGCACTCNTGTTT-ATTAC 120

MV_589246-1041_39_HMBSrevD05.ab1 GCCGCTGCAGGTTTAGAGCAGAGTTGACAATCTTCATTTGATTCTCCAGTGCAGCAATGC 360

* * **** * * * **** *** ** * * ** * * *

REV_AGM_CON_SEQ_1 CCC----------CTCACCCTCCAGCTTTGGTACCTGGGCAGGGACATGGATGGTAGCCT 170

MV_589246-1041_39_HMBSrevD05.ab1 CAGAGAAGCCAGCGGCTGCGGCCGGCAATTCTGACTGTGCATCGAATGGGGTAGGAGGCT 420

* * * ** ** * * *** *** ** ** * * ** **

REV_AGM_CON_SEQ_1 GCATGGTGTCTTGTATGCTATCTGAGCCATCTAGACTCCAGACTCCTCCAGT------ 222

MV_589246-1041_39_HMBSrevD05.ab1 TCGAGGAGCTCCT-------GGGGCCATCCTGAGACTCCAGACTCCTCCAGTCAGGTN 471

* ** * * ********************

Mosquito 40

REV_AGM_CON_SEQ_1 ------------------------------------------------------------ 0

MV_589246-1042_40_HMBSrevD06.ab1 NNNNNNNNNNNTNCCNTGCACTGNNTTACTCCNNANNNCTCCTCTCCGGTAANTCGTAAG 60

REV_AGM_CON_SEQ_1 ---------GTTACGAGCAGTGATGCCTACCAGCTGTGGGTCATCCTCAGGGCCATCTTC 51

MV_589246-1042_40_HMBSrevD06.ab1 TTCGATACACTTTGGCGGAGTGATAATGGCAGCTGTTGATTACTTTTCTCAAGCAGACTC 120

** * * ****** * ** * * ** ** **

REV_AGM_CON_SEQ_1 ATGCTGTATGCGGGAAGGAGGTGGGA-ATTGG---------------------------T 83

MV_589246-1042_40_HMBSrevD06.ab1 TTCTAGTACTCTGGAGTGGTGTGACTCATCTCTCAGTACCAGACTCCAGACTCCTCCAGT 180

* *** * *** * *** ** *

REV_AGM_CON_SEQ_1 GA---GAACAAATGAGATTATATGCACTCN--------TGTTTATTACCCCCTCAC---- 128

MV_589246-1042_40_HMBSrevD06.ab1 CAGGTAANCCCCNCATATAAAATAAATTNCTTCTTACCGTTTGAGTACTAAATAGCCACA 240

* * * * ** * ** * * ** * *** * *

REV_AGM_CON_SEQ_1 ----CCTCCAGCTTTGGTACCTGGGCAGGGACATGGATGGTAGCCTGCATGGTGTCTTGT 184

MV_589246-1042_40_HMBSrevD06.ab1 GAAACATAGAAATGCTGGAG-TTGGAAGAGACTCCGNACTCCTCCAGTCAGGT--AANCT 297

* * * * * * * ** ** *** * ** * *** *

REV_AGM_CON_SEQ_1 ATGCTATCTGAGCCATCTAGACTCCAGACTCCT--------------------------- 217

MV_589246-1042_40_HMBSrevD06.ab1 NGNCTCTGTCNCCTTCCGGGGCTGCAGGATCNGACCCCCTTGCGGGNAANCCNAGCCCCC 357

** * * * * * ** *** **

REV_AGM_CON_SEQ_1 -------CCAGT------------------------------------------------ 222

MV_589246-1042_40_HMBSrevD06.ab1 ATCTCAGACAGNGCTTGCCTCACCTGTGCGGAGGGTGTGAAGCGTGNGNTCANAACCTGG 417

***

REV_AGM_CON_SEQ_1 ----------------- 222

MV_589246-1042_40_HMBSrevD06.ab1 GCATCAGNATGGAANNN 434

Mosquito 41

REV_AGM_CON_SEQ_1 -------------------------------------GTTACGAGCAGTGATGCCTACCA 23

MV_589246-1043_41_HMBSrevD07.ab1 NNNNNNNGNTNNNNTGCANCANCTTTNNNTNNNNACCNNCATCAGCAGTGNGGCGTAACT 60

* ******* ** ** *

REV_AGM_CON_SEQ_1 ------------------------------GCTGT-GGGTCATCCTCAGGGCCATCTTCA 52

MV_589246-1043_41_HMBSrevD07.ab1 TCGATACACTTTAGCGATGTGATAATGGCAGCTGTTGATTACTTTTCTCCAGCAGACTCT 120

***** * * * ** ** **

REV_AGM_CON_SEQ_1 TGCTGTATGCGGGAAGGAGGTGGGA-ATTGGTGAGAACAA--ATGAGATTATATGCACTC 109

MV_589246-1043_41_HMBSrevD07.ab1 TCTAGTACTCTGGAGTGGTGTGACTCATCTCTCAGTACCAGACTCCAGACTCCTCCAGTC 180

* *** * *** * *** ** * ** ** * * * ** **

REV_AGM_CON_SEQ_1 NTGTTTATTACCC----------------------------------------------- 122

MV_589246-1043_41_HMBSrevD07.ab1 AGGTAAACTCCAGACTCCTCCAGTCAGGTAAGGNNTCTGGGTCTCGTGNCGNNTNGNNNT 240

** * * *

REV_AGM_CON_SEQ_1 --------------------------------------------CCTCACCCTCCAGCTT 138

MV_589246-1043_41_HMBSrevD07.ab1 GANNCGCTGCTATTNCNGNTGCCTAGNGNGGTTATATGGANGTGACTCCGACTCCGTCTN 300

*** **** **

REV_AGM_CON_SEQ_1 TGGTACCTGGGCAGGGACATGGATGGTAGCCTGCATGGTGTCTTGTATGCTATCTGAGCC 198

MV_589246-1043_41_HMBSrevD07.ab1 TTCTCCGGCTTTGGGGGANTAGCCGGTACCCTTNCGACTCCNGCTCCNGCTTTCANATAC 360

* * * *** * * **** *** * *** ** * *

REV_AGM_CON_SEQ_1 ATCTA---GACTCCAGACTCCTCCAGT- 222

MV_589246-1043_41_HMBSrevD07.ab1 ACCGACTCANACTCCAACTCCTCCNTCA 388

* * * * ********

Mosquito 42

REV_AGM_CON_SEQ_1 ------------------------------------------------------------ 0

MV_589246-1044_42_HMBSrevD08.ab1 NNNNNNNNNNNNCNNNNNNNGNNNNNNTNNNNACTCATCNNNTNTAGTAANAGCGTAAGT 60

REV_AGM_CON_SEQ_1 ------------------------------------------------------------ 0

MV_589246-1044_42_HMBSrevD08.ab1 TAGNCTGCTTTAGNGAGGTGAAGGGNGATCTGTTGATTACTGTTCTCCAGANCACTCTTC 120

REV_AGM_CON_SEQ_1 ----GTTACGAGCAGTGATGCCTACCAGCTGTGGGTCATCCTCAGGGCCATCTTCATGCT 56

MV_589246-1044_42_HMBSrevD08.ab1 TCANACTCTGGANAGGCGTGACTCATAACTCAGAACCNNACTCCAGACTCCTCCAGTCAG 180

* * ** ** ** * ** * * *** * * *

REV_AGM_CON_SEQ_1 GTATGCGGGAAGGAGGT---GGGAATTG-------GTGAG------AACAAATGAGATTA 100

MV_589246-1044_42_HMBSrevD08.ab1 GTAAGACNNCNGGTTGNNGGTGCCTTTCATCNGTGGAGCTCTGCANTANAATGCTTTGGA 240

*** * ** * * ** * * * ** *

REV_AGM_CON_SEQ_1 TATGCACTCNTGTTT--ATTAC--CCCCTCACCC---TCCAGCTTTGGTACCTGGGCAGG 153

MV_589246-1044_42_HMBSrevD08.ab1 TATTCANTTTCGTGTAAAGGATGCCCCTGCTCCCAGCGCCGGGGTTAATGCATGCGGTGC 300

*** ** * ** * * * *** * *** ** * ** * * ** * *

REV_AGM_CON_SEQ_1 GACATGGATGGTAGCCTGCATGGTGT---------------------------------- 179

MV_589246-1044_42_HMBSrevD08.ab1 A---AGANCGGACGCTTCCTCTGGGTCCAAACTCAGCTCTTCCTTTGAGTGACTTTCACT 357

* ** ** * * * **

REV_AGM_CON_SEQ_1 -----------------------------------------CTTGTATGCTATCTGAGCC 198

MV_589246-1044_42_HMBSrevD08.ab1 GCNAGAGTCCCTGAGAAGCCCTCACACTGCCTTCCCTGTCTCTCTTGAGGAGGGATCGCC 417

** * * ***

REV_AGM_CON_SEQ_1 A-------------------------------------------------TCTAGACTCC 209

MV_589246-1044_42_HMBSrevD08.ab1 ATGGANCCGCGAANNGNCGCGCGNGNTTCGCACGTATATCTGCCTGNAGGTGNNNACNCA 477

* * ** *

REV_AGM_CON_SEQ_1 AGACTCCTCCAGT---------- 222

MV_589246-1044_42_HMBSrevD08.ab1 AAATGCCTCTAGTCGNNNNNNNN 500

* * **** ***

Mosquito 43

REV_AGM_CON_SEQ_1 -----------------GTTACGAGCAGTGATGCCTACCAGCTGTGGGTCATCCTCAGGG 43

MV_589246-1045_43_HMBSrevD09.ab1 NNNNNNNNNNCNNNNANGNNCNNNNNACNGNTGCNTACCTANTGTGNGGNAACCTCA--N 58

* * * *** **** **** * * *****

REV_AGM_CON_SEQ_1 CCATCTTCATGCTGTATGCGGGAAGGAGGTGGGAATTGGTGAGAACAAATGAGATTATAT 103

MV_589246-1045_43_HMBSrevD09.ab1 GGATTCTTCTGTTGTATGAGGGAAGGACGTGGGATTTGGTG--ATAACTTCAAATTATAT 116

** * ** ****** ******** ****** ****** * * * * *******

REV_AGM_CON_SEQ_1 GCACTCNTGTTTATTACCCCCTCACCCTCCAGCTTTGGTACCTGGGCAGGGACATGGATG 163

MV_589246-1045_43_HMBSrevD09.ab1 CCCCTCTTGTATTCTACCCCCTCGCCCTCCAGCTTTGGTACCTGGGCNGGGACATGGATG 176

* *** *** * ********* *********************** ************

REV_AGM_CON_SEQ_1 GTAGCCTGCATGGTGTCTTGTATGCTATCTGAGCCATCTAGACTCCAGACTCCTCCAGT- 222

MV_589246-1045_43_HMBSrevD09.ab1 GTAGCCTGCATGGTCTCTTGTATGCTATCTGAGCCGTCTAGACTCCAGACTCCTCCAGTC 236

************** ******************** ***********************

REV_AGM_CON_SEQ_1 ------------------------------------------------------------ 222

MV_589246-1045_43_HMBSrevD09.ab1 AGGTAANGNTTTNGCANCTTNNNTNGTTNNATCATGAAANAGCCCCACCCTTTTGACTAN 296

REV_AGM_CON_SEQ_1 ------------------------------------------------------------ 222

MV_589246-1045_43_HMBSrevD09.ab1 ACATCAGGGGGGTCTGGGGCTCCNNGCCAGGACCCCNTTGCATTGCAAAGCAATCCCCCA 356

REV_AGM_CON_SEQ_1 ------------------------------------------------------------ 222

MV_589246-1045_43_HMBSrevD09.ab1 TCTTCAACAGCCCATCACTGCCCTGTCCCCCGCCAGGAAACCTGGGATCAGAACCTGGGC 416

REV_AGM_CON_SEQ_1 --------------- 222

MV_589246-1045_43_HMBSrevD09.ab1 ATCAGCANGGCANNN 431

Mosquito 44

REV_AGM_CON_SEQ_1 ------------------------------------------------------------ 0

MV_589246-1046_44_HMBSrevD10.ab1 NNNNNNGNNTCNNNGCNNCTTNTNATTAANACCAGCATCAGCAGTGTGGCGTAACTTCGA 60

REV_AGM_CON_SEQ_1 ------------------------------------------------------------ 0

MV_589246-1046_44_HMBSrevD10.ab1 TACACTTTAGTAAATGTGATAATGGCAGCTGTTGATTACTTTTCTCCAGCAGACTCTTCT 120

REV_AGM_CON_SEQ_1 -GTTACGAGCAGTGATGCCTACCAGCTGTGGGTC--ATCCTCAGGG-------------- 43

MV_589246-1046_44_HMBSrevD10.ab1 AGTACTCTGGAGTGGTGTGACTCATCTCTCAGTACCAGACTCCAGACTCCTCCAGTCAGG 180

** * **** ** ** ** * ** * *** *

REV_AGM_CON_SEQ_1 -CCATCTTCATGCTGTATGCGGGAAGGAGGTGGGA----------------------ATT 80

MV_589246-1046_44_HMBSrevD10.ab1 TAATTNGAGATCGACNGGGAGGTAAGAGNGCAGCAGCTGCGGCAGCTGATTCAGNNTATT 240

* ** * ** *** * * * ***

REV_AGM_CON_SEQ_1 GGTGAGAACAAAT--GAGATTATATGCACTCNTGTTTATTACCCCCTCACCCTCCAGCTT 138

MV_589246-1046_44_HMBSrevD10.ab1 ACTCTGAAGCAAATGGANAGCTTAATCAGGTGCCGTAGCTGCTGCGCCATCATCTCCCTT 300

* *** ** ** * ** ** * * * * ** * ** ***

REV_AGM_CON_SEQ_1 TGGTACCTGGGCAGGGACATGGA-----------TG------------GTAGCCTGCATG 175

MV_589246-1046_44_HMBSrevD10.ab1 TCCATCATAAAATGGCTGATGACGATGAGGGCTTTGGCCAGCTGCTACTCCTCCACCAGG 360

* * * ** *** ** ** ** *

REV_AGM_CON_SEQ_1 GTGTCTTGTATGCTATCTGAGCCATCTAGACT-----------------------CCAGA 212

MV_589246-1046_44_HMBSrevD10.ab1 ATGTGCTGGAGCTCATCTGTGCGGTCCCGCTGACAGTGCGTCTGANTCAGGAGCTGCAGA 420

*** ** * ***** ** ** * ****

REV_AGM_CON_SEQ_1 CTCCTCCAGT-------------------------------------------------- 222

MV_589246-1046_44_HMBSrevD10.ab1 TTGATCNATTCGATCNNTCNTAACGATCAGANTGCNNGCGNNNTNNTNNNNNTCCTCNAC 480

* ** * *

REV_AGM_CON_SEQ_1 ------------------------------------------------------- 222

MV_589246-1046_44_HMBSrevD10.ab1 AACNANNTNNANNNGANTGNCNNNCTCTATCAGAANCNGGGGCATCANCCTGGNN 535

Mosquito 45

REV_AGM_CON_SEQ_1 ------------------------------------------------------------ 0

MV_589246-1047_45_HMBSrevD11.ab1 NNNNNNNTTGTNNCGGCACACTGGAATTTCCCGGCCTGGAGAGCTGTAGCTCCCTCCCGG 60

REV_AGM_CON_SEQ_1 --------------------GTTACG---------------------------------- 6

MV_589246-1047_45_HMBSrevD11.ab1 ACCAAAGCCTACGAGGGCAGCTTATCGGCGGCGCGACAGATCAGCCGTCGGGGCTCAAGC 120

***

REV_AGM_CON_SEQ_1 -------AGCAGTGATGCCTACCAGCTGTGGGTC---------------ATCC------- 37

MV_589246-1047_45_HMBSrevD11.ab1 CCTGAGCAAATGTGGTGCGGGACTGCTGGGTGTCTGTCACCTCTGAGCTGTCCTAGAAGG 180

* *** *** * **** * *** ***

REV_AGM_CON_SEQ_1 ------------------------------------------------------------ 37

MV_589246-1047_45_HMBSrevD11.ab1 CAAGGGGGCTTAAACTGCTCCATCCAAAGACTCCAGACTCCTCCAGTCAGGTAANNGTTT 240

REV_AGM_CON_SEQ_1 -------------------------------TCAGGGCCATCTTC-----ATGCTGTATG 61

MV_589246-1047_45_HMBSrevD11.ab1 GCGTGACACAGCACACTGCCTGGCTTAAACAGCTTTGCCCTGTCNGACCACTGCTCGGTG 300

* *** * * **** **

REV_AGM_CON_SEQ_1 CGGGAAGGAGGTGGGAATTGGTGAGAACAAATGAGATTATATGCACTCNTGTTTATTACC 121

MV_589246-1047_45_HMBSrevD11.ab1 TGAAGGAGATCTNGGAGTAGTTCACAA---GGGAACTGGCCTGACATTTGGNAGGCTCCC 357

* ** * *** * * * * ** ** * ** * * * **

REV_AGM_CON_SEQ_1 CCCTCACCC-----TCCAGCTTTGGTACCTGGGCAGGGACATGGATGGTA--GCCTG--- 171

MV_589246-1047_45_HMBSrevD11.ab1 ACGTCAGTTGAGTGCGGGACTGAGATTTCTGGGTCGGGCGAGGCAGAATATGGGCTCGGT 417

* *** ** * * ***** *** * * * ** * **

REV_AGM_CON_SEQ_1 -----CATGGT--------------------------------------GTCTT------ 182

MV_589246-1047_45_HMBSrevD11.ab1 GCCCTTATCCTGACCACACATCACNCCTGGGGGTGCAACCAGCCTTCCTGCCCTGCCTGC 477

** * * * *

REV_AGM_CON_SEQ_1 ------------------GTATGCTATCTGAGCCATCTAGACTCCAGACTC--------- 215

MV_589246-1047_45_HMBSrevD11.ab1 CACNGCTNTGAATTCCTTGGGGTGCATCTGTGTGGNGAGNNNTTGAGATAANNNGGCTGT 537

* ***** * * ***

REV_AGM_CON_SEQ_1 -CTCCAGT------ 222

MV_589246-1047_45_HMBSrevD11.ab1 CNACCAGCCANNAA 551

****

Mosquito 46

REV_AGM_CON_SEQ_1 ------------------------------------------------------------ 0

MV_589246-1048_46_HMBSrevD12.ab1 NNNNNNNNNTNNNNNNCNCNGCTTNACTNNNNNNTCNTCCTCTNNGGTAANNTGTAACTT 60

REV_AGM_CON_SEQ_1 ------------------------------------------------------------ 0

MV_589246-1048_46_HMBSrevD12.ab1 CGATACACTTTAGCGATGTGATAATGGCAGCTGTTGATTACTTTTCTCCAGCAGACTCTT 120

REV_AGM_CON_SEQ_1 ---GTTACGAGCAGTGATGCCTACCAGCTGTGGGTC--ATCCTCAGGGCCATCTTCATGC 55

MV_589246-1048_46_HMBSrevD12.ab1 CTAGTACTCTGGAGTGGTGTGACTCATCTCTCAGTACCAGACTCCAGACTCCTCCAGTCA 180

** * **** ** ** ** * ** * *** * * *

REV_AGM_CON_SEQ_1 TGTATGCGGGAAGGAGGTGGGAATTGGTGAGAACAAATGAGATTATATGCACTCNTGTTT 115

MV_589246-1048_46_HMBSrevD12.ab1 GGTAANTGGGTTGGGTTTCAAAGGCTGAGNGTGGNAAAAGAGTCNAGTGCAGAAAA-GTN 239

*** *** ** * * * * * ** * **** *

REV_AGM_CON_SEQ_1 ATTACCCCCTCACCCTCCAGCTTTGGTACCTGGGCAGGGACATGGATGGTAGCCTGCATG 175

MV_589246-1048_46_HMBSrevD12.ab1 ANAATCCGCTGCAGTTGCNGCNTTNNTTGGGNGGTTACCACGAAACTGACGAATTGG-TG 298

* * ** ** * * ** ** * ** ** ** ** **

REV_AGM_CON_SEQ_1 GTGTCTTGTATGCTATCTGAGCCATCTA---------------GACTCCAGACTCCTCCA 220

MV_589246-1048_46_HMBSrevD12.ab1 GT-GGTTTTATGAGTTCAGGGGCCTCCGGTCGGGATAATNNNANACTCCTTACTCATCCA 357

** ** **** ** * * * ** ***** **** ****

REV_AGM_CON_SEQ_1 GT---------------------------------------------------------- 222

MV_589246-1048_46_HMBSrevD12.ab1 NAAAGNNACACNTCNGGCACTCTTAAANGNAAATGAAAAATTNGACAGNGAGGACTGACA 417

REV_AGM_CON_SEQ_1 ------------------------------------------------------------ 222

MV_589246-1048_46_HMBSrevD12.ab1 TACAACAAAGCAAGTTCCGAACCATAGGNGCGGGCGGCTTAAAACAATNATATCCTNGGT 477

REV_AGM_CON_SEQ_1 --- 222

MV_589246-1048_46_HMBSrevD12.ab1 ACN 480

Mosquito 47

REV_AGM_CON_SEQ_1 ---GTTACGAGCAGTGATGCCTACCAGCTGTGGGTCATCCTCAGGGCC--------ATCT 49

MV_589246-1049_47_HMBSrevE01.ab1 NNNNNNNNNNNNNGNNGCTGCNCTTNNNGTTGCCTNNTNCTCNGNGTCGTGGCGTNGACT 60

* * ** * * *** * * * **

REV_AGM_CON_SEQ_1 TCATGCTGTATGCGGGAAGGAGGTGGGAATTGGTGAGAACAAATGAGATTATATGCACTC 109

MV_589246-1049_47_HMBSrevE01.ab1 TGNTANTGCNNGTNGATGTGATGTGTGGGATGTTGATTTCTTTTCACAATCAAACCCTTC 120

* * ** * * ** *** * ** *** * * * * * * * **

REV_AGM_CON_SEQ_1 NTGTTTATTACCCCCTCACCCTCCAGCTTTGGTACCTGGGCAGGGACATGGATGGTAGCC 169

MV_589246-1049_47_HMBSrevE01.ab1 TCGTACTCTANCCCCTNGTGANTCNTNNAT-NGNTACCAGACTCCAAACTCCTGNTTCCC 179

** ** ***** * * * * * ** * **

REV_AGM_CON_SEQ_1 TGCATGGTGTCTTGTATGCTATCTGAGCCATCTAGACTCCAGACTCCTCCAGT------- 222

MV_589246-1049_47_HMBSrevE01.ab1 GTAANGGTCTCTTGTATGCTATCTGAGCCGTCTAGACTCCAGACTCCTCCAGTCAGGTAA 239

* *** ******************** ***********************

REV_AGM_CON_SEQ_1 ------------------------------------------------------------ 222

MV_589246-1049_47_HMBSrevE01.ab1 NGATATACGAGTCCNNATTCCTCCAATCCGGTAANTAGCNCNCCCTTTCGGGCGGTTNCT 299

REV_AGM_CON_SEQ_1 ------------------------------------------------------------ 222

MV_589246-1049_47_HMBSrevE01.ab1 TTCTCCNTCTGGGGCTCNNGAACGGACCCCCTTGCATTGGAACCCAATCTCCCAGCTCCA 359

REV_AGM_CON_SEQ_1 ------------------------------------------------------------ 222

MV_589246-1049_47_HMBSrevE01.ab1 ACAGCGCANGCCTGACCTGTGCGGAGGCCCGGANACCTGGGATCGGAACCTGGGNNACAG 419

REV_AGM_CON_SEQ_1 ---------- 222

MV_589246-1049_47_HMBSrevE01.ab1 CANGGCAANN 429

Mosquito 48

REV_AGM_CON_SEQ_1 ------GTTACGAGCAGTGATGCCTACCA----GCTGTGGGTCATCCTCAGGGCCATCTT 50

MV_589246-1050_48_HMBSrevE02.ab1 NNNNNNNNNNCGNGNTNNNNNANCTGCNGNACTCCNGACTCCTCCAGTCAGGTAANACTC 60

** * ** * * * ***** **

REV_AGM_CON_SEQ_1 CATGCTGTATGCGGGAAGGAGGTGGGAATTGGTGAGAACAAATGAGATTATATGCACTCN 110

MV_589246-1050_48_HMBSrevE02.ab1 CAGACTC----CTCCAGTCAGGT----------------AANTGTGGTTCTGGCTGCTGT 100

** ** * * **** ** ** * ** * **

REV_AGM_CON_SEQ_1 TGTTTATTACCCCCTCACCCTCCAGCTTTGGTACCTGGGCAGGGACATGGATGGTAGCCT 170

MV_589246-1050_48_HMBSrevE02.ab1 TGATTACTTTTCTCG--AGCTCACTCTTCTAGTACTCTGGAGTGGTGTGACTCATCTCTC 158

** *** * * * *** *** ** * ** * ** * * *

REV_AGM_CON_SEQ_1 GCATGGTGTCTTGTATGCTATCTGAGCCATCTAGACTCCAGACTCCTCCAGT-------- 222

MV_589246-1050_48_HMBSrevE02.ab1 AGTACCAGACTCCAGACTCCTCCAGTCAGGTAANACTCCNGACTCCTCCAGTCAGGTAAG 218

* ** ** * * ***** ************

REV_AGM_CON_SEQ_1 ----------------------------------------------------- 222

MV_589246-1050_48_HMBSrevE02.ab1 CNNCCTCGNACGNNNCNGGAGNGTNNTCCNAAACNCNAGCCAACTNNTCATGT 27

Mosquito 49

REV_AGM_CON_SEQ_1 ---GTTACGAGCAGTGATGCCTACCAGCTGTGGGTCATCCTCAGGGCCATCTTCATGCTG 57

MV_589246-1051_49_HMBSrevE03.ab1 NNNNNNNNNNNNNTNNTNNNCNNNTGACTNNNACTCNTCCTCTNGGTAANNNNGATTTNA 60

* ** ** ***** ** * **

REV_AGM_CON_SEQ_1 T----ATGCGGGAAGGAGGTGGGAATTGGTGAGAA------------------------- 88

MV_589246-1051_49_HMBSrevE03.ab1 TACTTTTGCAATGTGATAATGGNAATTGTTGAGNATTTTCTTCTTGGACTCTNNCCTACT 120

* *** * *** ***** **** *

REV_AGM_CON_SEQ_1 ------------------------------------------------------------ 88

MV_589246-1051_49_HMBSrevE03.ab1 CCTCCATGGTGGTAATCATCTCTCAGTACCAGACTCCAGACTCCTCCAGTCAGGTAANGA 180

REV_AGM_CON_SEQ_1 ------------------------------------------------------------ 88

MV_589246-1051_49_HMBSrevE03.ab1 NAGGCGGGCTCACCACGGGCGCGTCCCNNNANCTACACCCGATACAAAAAATTTNTNATT 240

REV_AGM_CON_SEQ_1 ----CAAATG-------------------------------------------------- 94

MV_589246-1051_49_HMBSrevE03.ab1 CCACCGCATAAGGAAANGGGAAACANTGTCTCCCGGTGCAGAATNCTGTGGAGCTCGGTT 300

* **

REV_AGM_CON_SEQ_1 --------------------------------AGATTATATGCACTCNTGTTTATTACCC 122

MV_589246-1051_49_HMBSrevE03.ab1 GANTTCNNACCGTAGGANGGTGAGTGGCTCANAAGATACTGGGCCTGGTGTT-----CAC 355

* ** * ** **** * *

REV_AGM_CON_SEQ_1 CCTCACCCTCCAGCTTTGGTACCTGGGCAG----------------GGACATG-----GA 161

MV_589246-1051_49_HMBSrevE03.ab1 CCGGACGCTGAATCAANGNTNCCCGAACTGNNAANANATTAAGTAGGGTTTTGACTGCCN 415

** ** ** * * * * ** * * * ** **

REV_AGM_CON_SEQ_1 TGGTAGCCTGCATGGTGTCTTGTATGCTATCTGAGC---------------------CAT 200

MV_589246-1051_49_HMBSrevE03.ab1 TGGATGATGCNGAGGTGCCTTTGATGCCTGCGCCAGTCACGACAACCCTCGCGCGACACT 475

*** * **** *** **** * *

REV_AGM_CON_SEQ_1 CTAGACTCCAGACTCCTCC----------------------------------AGT---- 222

MV_589246-1051_49_HMBSrevE03.ab1 CNNNTGTCCAAGTTTCTNCTAANANGTCTGCCNNAGAANGCANTNCCTCTGGCTGTTCTT 535

* **** * ** * **

REV_AGM_CON_SEQ_1 ------------------------------------------------------------ 222

MV_589246-1051_49_HMBSrevE03.ab1 GGNNNNNGCGNTNAGAGAACTGTCTGTGCATNNGTNAGAAGNACNNGCAACCCTCGNCTG 595

REV_AGM_CON_SEQ_1 ------------------------------------ 222

MV_589246-1051_49_HMBSrevE03.ab1 CAGATAACCATGNANAANCTGGACCNCNNNNNNNNN 631

Mosquito 50

REV_AGM_CON_SEQ_1 ------------------------------------------------------------ 0

MV_589246-1052_50_HMBSrevE04.ab1 NNNNNNNNNNNNNNNNNNNANTNNTTAATCGTTTCCNNTACCTCCTCTATCGGCCGTGGG 60

REV_AGM_CON_SEQ_1 ---------------------GTTACGA----GCAGTGATGCCTACC------------- 22

MV_589246-1052_50_HMBSrevE04.ab1 ACTCGAGGACCCCATATGGGCCTTCTGAGCCCGCCCCGTTGACTGCCGTTGGTCGGATCN 120

** ** ** * ** ** **

REV_AGM_CON_SEQ_1 -AGCTGTGGGTCATCCTCAGGGCCA----------TCTTCATGCTGTATGCGGGAAGGAG 71

MV_589246-1052_50_HMBSrevE04.ab1 CTGCANGGGGTTTTCCCCGGTTCCCCCTCAGNACTTCGGTGTCCGGTAAGAGAGCCGTAC 180

** **** *** * * ** ** * * *** * * * * *

REV_AGM_CON_SEQ_1 GTGGGAATTGGTGAGAACAAATGAGATTATATGCACTCNTGTTTATTA----CCCCCTCA 127

MV_589246-1052_50_HMBSrevE04.ab1 CATTGAACTGGTAAAAGACCGTAAACTCCANACTCCTCCAGTCAGGTAACTGAGACTCCA 240

*** **** * * * * * *** ** ** * **

REV_AGM_CON_SEQ_1 CCCTCCAGCTTTG----------------------------------------------- 140

MV_589246-1052_50_HMBSrevE04.ab1 GACNCCTGCTGTCNNGTAANGTTGGGCTTNGGNCGTTCNTNCGTGGTTTGGTTGGCCTTC 300

* ** *** *

REV_AGM_CON_SEQ_1 -GTACCTGGGCAGGGACA----------------TGGATGGTAGCCTGCATGGTGTCTTG 183

MV_589246-1052_50_HMBSrevE04.ab1 GATACCTCTCTAAGAAGTCGTCTGCNNNANTGCGTNGCAGAATGCCTTCTTGAGCTGATA 360

***** * * * * * * **** * ** * *

REV_AGM_CON_SEQ_1 T--------------------------ATGCTATCTGAGCCATCTAGACTCCAGACTCCT 217

MV_589246-1052_50_HMBSrevE04.ab1 GAANTTACGTGTTGCGTGAGACCGNCNCTGTCGTTCTCNAATTCCAGACTCCNNAATNCT 420

** * ** ******* * * **

REV_AGM_CON_SEQ_1 CCAGT------------------------------------------------------- 222

MV_589246-1052_50_HMBSrevE04.ab1 CNNGTNATGTAAACGAACTCNGGGCTGCCCGCGGATCGATTGGAAACGTTTTCTTGATTG 480

* **

REV_AGM_CON_SEQ_1 ------------------------------------------------------------ 222

MV_589246-1052_50_HMBSrevE04.ab1 CCAAAACGTTCCACANGATTGNTCCCCCGGGCGNTNTGCTCCGCTNTCACTTTGTTCCGC 540

REV_AGM_CON_SEQ_1 ------------------------------------------------------------ 222

MV_589246-1052_50_HMBSrevE04.ab1 CGCGTGATTGTCCAGATCNNTGCNTCTTGATGAACGACTCNCAACTCCTCCNATCGNGAA 600

REV_AGM_CON_SEQ_1 ------------------------------------------------------------ 222

MV_589246-1052_50_HMBSrevE04.ab1 AGANTCNTCTGCGTCNTTCGTCNTGAGCTGNANNTCCTGTTACCGNTTGCTNANNNNGGA 660

REV_AGM_CON_SEQ_1 ------------------------------------------------------------ 222

MV_589246-1052_50_HMBSrevE04.ab1 GATTCGGACACTGAATAGCTCTTTCANCTTCTTCACCATTTCNNNNACGAGTACTCACGA 720

REV_AGM_CON_SEQ_1 ------------------------------------------------------------ 222

MV_589246-1052_50_HMBSrevE04.ab1 TGGNGCNTCGGGAGCTGAANTTGACNTACTTGTCGTGCACCGTCACNCTGAGGCTTNCNT 780

REV_AGM_CON_SEQ_1 ------------------------------------------------------------ 222

MV_589246-1052_50_HMBSrevE04.ab1 AACCGCACTTTTTGCGCATCGTCCAACTTCGCTCNGTCCTTGAGGAAGAGGTCTTCGTAC 840

REV_AGM_CON_SEQ_1 ------------------------------------------------------------ 222

MV_589246-1052_50_HMBSrevE04.ab1 TTTCGGTACCACCGGTCGAAAANGAGCCCGTTGNNCTGGNTCGTNACNAATTCCCGGNTG 900

REV_AGM_CON_SEQ_1 ----------------------------------------- 222

MV_589246-1052_50_HMBSrevE04.ab1 TTGNNNGGCNAAGACNTCCAGANCTNCNTCAGNNAGGTAAA 941

Mosquito 51

REV_AGM_CON_SEQ_1 ------------------------------------------------------------ 0

MV_589246-1053_51_HMBSrevE05.ab1 NNNNNNNNNGNNTNNGCCNTTGTAGCCNTCNNNCGTCTGNANTATACCNATAAGATAGNA 60

REV_AGM_CON_SEQ_1 -----GTTACGAGCAGTGATGCCTACCA-------------------------------- 23

MV_589246-1053_51_HMBSrevE05.ab1 AGGTGNGCACGAAGATCGAGGTCTTCGNGCGATGTTCTACCACATGGTAGAAATATTGCT 120

**** * ** * ** *

REV_AGM_CON_SEQ_1 ----------------------------GCTGTGGGTCATCCT----------------- 38

MV_589246-1053_51_HMBSrevE05.ab1 CTTTATCTGTGATATCGTTAAACGACTAGGAGTAGTTCATGTTCAGACTCCAGACTCCTC 180

* ** * **** *

REV_AGM_CON_SEQ_1 ------------------------------------------------------------ 38

MV_589246-1053_51_HMBSrevE05.ab1 CAGTCAGGTAAACCGGNNACAGACCTTAGACGCCAGACTCCTCCAGTCAGGTAANNACCG 240

REV_AGM_CON_SEQ_1 -------------CAGGGCCATCTTCATGCTGTATG------------------------ 61

MV_589246-1053_51_HMBSrevE05.ab1 TTTGGGGCGCCATCGCCGCATCCTTGTCTGNGTCCCTTTGTANAATGGGCTGTTNGGCAC 300

* ** *** **

REV_AGM_CON_SEQ_1 -----CGGGAAGGAGG-----------------------------TGGGAATTGGTGAGA 87

MV_589246-1053_51_HMBSrevE05.ab1 CNAGGNGNNGGCGATGTCNNAGTTGGNCGCNGAGGCTCCAACGCCNNCAAANNGGGGATA 360

* ** * ** ** ** *

REV_AGM_CON_SEQ_1 ACAAATGAGATTATA-----TGCACTCNTGTTTATTACCCCCTCACCCTCCAGCTTTGGT 142

MV_589246-1053_51_HMBSrevE05.ab1 ACCCAGGNGNGTACATCCTGCGCAAGCTTTACGGCTTCCGCTTCAAACTCCAGACTCCTC 420

** * * * ** * *** * * * ** * *** ****** *

REV_AGM_CON_SEQ_1 ACCTGGGCAGGGACATGGATGGTAGCCTGCATGGTGTCTTGTA---TGCTATCTGAGCCA 199

MV_589246-1053_51_HMBSrevE05.ab1 AANTCTGGGAAANNCCTGACGCCCCCGTGGCTGNAANCTGCTCTTTTGGAATCGTGNNCN 480

* * * ** * * ** ** ** * ** *** *

REV_AGM_CON_SEQ_1 TCTAGACTCCAGACTCCTCCAG---------------------------T---------- 222

MV_589246-1053_51_HMBSrevE05.ab1 NNTTGACAGNANGGTGCCCNTGCTTGNTCNTGGAGCTTTACTTCNCCNNTCAGGNNNNNC 540

* *** * * * * * *

REV_AGM_CON_SEQ_1 --------------------------------------------- 222

MV_589246-1053_51_HMBSrevE05.ab1 NNNANNGGNTTTCCGGATGANNTATCATGATGAACGNCTCGGACT 585

Mosquito 52

REV_AGM_CON_SEQ_1 ------------------------------------------------------------ 0

MV_589246-1054_52_HMBSrevE06.ab1 NNNNNNNNNNNNNNNCCCATANAGCCNNNTNNNTCTGTANTATATCTATTNTATAGTAAG 60

REV_AGM_CON_SEQ_1 --------------------------------------------GTTACGAGCAGTGATG 16

MV_589246-1054_52_HMBSrevE06.ab1 GGGTGCTCGGACATCTTTTGTCTTCGTGCGATGTTCTACCGCCTGGTAAAATTATTGCTC 120

* ** * * ** *

REV_AGM_CON_SEQ_1 CCTAC------------------------------------------------------- 21

MV_589246-1054_52_HMBSrevE06.ab1 TTTATCTGTCATATCTTAAACGACTCCGAATCNTTCCTGTTCAGACTCCANACTCCTCCA 180

**

REV_AGM_CON_SEQ_1 ------------------------------------------------------------ 21

MV_589246-1054_52_HMBSrevE06.ab1 GTCAGGTAAAACGGTAACNGACCTTANACTCCNGACTCCTCNANTCCGGTAATCTGGTAA 240

REV_AGM_CON_SEQ_1 ------CA---------------------------------------GCTGTGGGTCATC 36

MV_589246-1054_52_HMBSrevE06.ab1 TCNTGAGATTTNTCCGTACCCNGCTCGGCCCTCCTCCTCACAAAGGGCCCGCNGGGNNCC 300

* * * ** *

REV_AGM_CON_SEQ_1 CTCAGGGCCATCTTCATGCTGTATG----------------CGGGAAGGAGGTGGGAATT 80

MV_589246-1054_52_HMBSrevE06.ab1 CTCGAGACGGCTCTCANGCTGCAGGTCGCNGTGGCTCACATAGCCAACAAACTGGGCATA 360

*** * * *** **** * * * ** * **** **

REV_AGM_CON_SEQ_1 GGTGAGAACAAATGAGATTATATGCACTCNTGTTTATTA---CCCCCTCACCCTC---CA 134

MV_589246-1054_52_HMBSrevE06.ab1 TCCCAGGGANAAAACNCT---TTNAGCTCNATTTNCTAGATCCCTCCGCAATGACACGAT 417

** ** * * **** ** * ** ** ** *

REV_AGM_CON_SEQ_1 GCTTTGGTACCTGGGCAGGGACATGG--------ATGGTAGCCTGCATGG--------TG 178

MV_589246-1054_52_HMBSrevE06.ab1 GCTCTAATCCCGGGGAAAAATGACTCATTCNGGGCTGTAAGCGNAAGTNTTGTTTCNNTT 477

*** * * ** *** * * ** *** * *

REV_AGM_CON_SEQ_1 TCTTGTATGCTATCTGAGCCATCTAGACTCCAGACTCCTCCAGT---------------- 222

MV_589246-1054_52_HMBSrevE06.ab1 TCTTGTTTGCCAAGANTTTCCACTCGACTTCCTACTCCTCGCGTTTAGGTGCNNCTTCCC 537

****** *** * * ** **** * ******* **

REV_AGM_CON_SEQ_1 ------------------------------------------------------------ 222

MV_589246-1054_52_HMBSrevE06.ab1 TTTGTTCTGGACAATGTTTTCCNCNNANGTGCTTCAAGATGTCGTACNCGNNNNNCNGAG 597

REV_AGM_CON_SEQ_1 ---------------------------------- 222

MV_589246-1054_52_HMBSrevE06.ab1 GTTGGTTTANNGTTGACCATTTTGGANTGCCCTC 631

Mosquito 53

REV_AGM_CON_SEQ_1 ------------------------------------------------------------ 0

MV_589246-1055_53_HMBSrevE07.ab1 NNNNNNNNGATNCAGACTCCNAACCTCAGACTCAAGACTGCAGGCTCGGGAGTACAGACT 60

REV_AGM_CON_SEQ_1 --------------------------------GTT------------------------- 3

MV_589246-1055_53_HMBSrevE07.ab1 CCAGACTCCTCCCCCCAGGTAANTANTCGGGTCTTCCGGTAACTCCAAACTTCNCCCCGC 120

**

REV_AGM_CON_SEQ_1 ------------------------------------------------------------ 3

MV_589246-1055_53_HMBSrevE07.ab1 CACAGACTCCGAACTCCTCCATTCGGGTAAAGCGTCNCGACTGGTTGCCAGTCTGGTCTC 180

REV_AGM_CON_SEQ_1 --ACGAGCAGTGATGCC------------------------------TACCAGCTGTGGG 31

MV_589246-1055_53_HMBSrevE07.ab1 ATACNGCCATTTATACTTCTCCTCCTGTACTGGAGAGCCAGCCACGCCCCTNCATCTTTC 240

** ** * ** * * * *

REV_AGM_CON_SEQ_1 TCATCCTCAGGGCCATC---------------------------------------TTCA 52

MV_589246-1055_53_HMBSrevE07.ab1 TCCGACTGAGGACANNCTTTAATGCTATGCTACNCCAAGGGCCTAGCGCGCATGGATTTA 300

** ** *** * * ** *

REV_AGM_CON_SEQ_1 TGCTGTATGCGGGAAG-------------------------------------------- 68

MV_589246-1055_53_HMBSrevE07.ab1 TCGCGAATGCGGGACTCCTTGAGACTTTCNACTTCTCCATTAAGGTAATTCNNNCATCNT 360

* * ********

REV_AGM_CON_SEQ_1 ---------------GAGGTGGGAATT--------------------------------- 80

MV_589246-1055_53_HMBSrevE07.ab1 CTTCTCGGCGGGGCTCCCTTGTTAAATGCGCCGTCCCAGTGGATTTTCTGCCGGGACCGC 420

** ** *

REV_AGM_CON_SEQ_1 ---------------------------------------------GGTGAGAACAAATGA 95

MV_589246-1055_53_HMBSrevE07.ab1 TGCCCTTNNGCTACTCTCTCGGGTTTTGAACGTGCTGATCAGCTTAGCGGTTACTACTCC 480

* * ** * *

REV_AGM_CON_SEQ_1 GATTATATGCACTCNTGTTTAT------TACCCCCTCACCCTCCAGCTT----------- 138

MV_589246-1055_53_HMBSrevE07.ab1 GATTATTAATTGGGGGGTTACATNGNNNGACCTCCTCAGGCGCAACTTCTTCNNGTGCGG 540

****** *** *** ***** * * * *

REV_AGM_CON_SEQ_1 ---TGGTACCTGGGCAGGGACATGGATGGTAGC--------------------------- 168

MV_589246-1055_53_HMBSrevE07.ab1 ATGAAGATCANGAAGNTCGTCATANACCACNGNGTGNTGGGGTAGTTTTAACTGCNCCAT 600

* * * * *** * *

REV_AGM_CON_SEQ_1 ------------------------------CTGCAT--GGTGTCTTGTATGCTATCTGAG 196

MV_589246-1055_53_HMBSrevE07.ab1 NGTCCTGNGTCTCAACAGACAGCTCNTGTTCTNANTNCGGACTGCAATCNTAAANTCGAA 660

** * ** * * * **

REV_AGM_CON_SEQ_1 CCATCTAGACTCCAGACTCCTCCAGT------ 222

MV_589246-1055_53_HMBSrevE07.ab1 GCGTTGAGACTCCNNACTCCTCCANTCAGNNN 692

* * ******* ********* *

Mosquito 54

REV_AGM_CON_SEQ_1 ------------------------------------------------------------ 0

MV_589246-1056_54_HMBSrevE08.ab1 NNNNNNNNNNNNNNNNNNNNTAGAATCCTTTTGTGTCTGNANTATATCCTCTATATACCN 60

REV_AGM_CON_SEQ_1 ---------------------------------------------GTTACGAGCAGTGAT 15

MV_589246-1056_54_HMBSrevE08.ab1 TGGGGNGCGCGGACATCNATTGTCTTNNCNGATGTNNTACCGCATGGTGCAGTTATTGCT 120

* * * * ** *

REV_AGM_CON_SEQ_1 GCCTAC------------------------------------------------------ 21

MV_589246-1056_54_HMBSrevE08.ab1 CTTTATCTGTTATGGTTTTGACCGACTCCCAATATTGCNCGTTCNGACTCANNACTCCTC 180

**

REV_AGM_CON_SEQ_1 ------------------------------------------------------------ 21

MV_589246-1056_54_HMBSrevE08.ab1 CACTCCAGTAAAACGGTAACAGAACTTAGACTCCAGACTCCTCCAGTCAGGTAANNGGNA 240

REV_AGM_CON_SEQ_1 ------------------------------------------------------------ 21

MV_589246-1056_54_HMBSrevE08.ab1 TTCCGGACTCCTTCNNTCNGGTAATGTGNGGTCCCTNNGCCGGCCNGGCTGTTTGNCTTG 300

REV_AGM_CON_SEQ_1 ------------------------------------------------------------ 21

MV_589246-1056_54_HMBSrevE08.ab1 ACTTCNANTNCTTTCNNAATCCTTGACGCNCAACCTCAAAACTNGTACCCCACTTTTGAN 360

REV_AGM_CON_SEQ_1 ------------------------------------------------------------ 21

MV_589246-1056_54_HMBSrevE08.ab1 TGTATAAAATGTACCTCNTGCGCAANCNATACGGCCTGCGTTTAAGACTTCAGACTTCTC 420

REV_AGM_CON_SEQ_1 ------------------------------------------------------------ 21

MV_589246-1056_54_HMBSrevE08.ab1 AACTCACGGCAATNNGNACTCCTCATATCTTTNAACCAGNTACTGCATTTTGATCCNGCC 480

REV_AGM_CON_SEQ_1 ------------------------------------------------------------ 21

MV_589246-1056_54_HMBSrevE08.ab1 CCGANGTCTGGTTCCATTGTGNACCGAAACCATCAGNGGAAANGAAGGTNTCTGACGCGC 540

REV_AGM_CON_SEQ_1 CAGCTGTGGGTCATCCTCAGGGCCAT---------------------------------- 47

MV_589246-1056_54_HMBSrevE08.ab1 CCGCTCTTGGGCNNGCTGACCACAAAGACATCACTGATTCAGGAGGCNGCNNACNGCGAN 600

* *** * ** * ** * * *

REV_AGM_CON_SEQ_1 --------------------------------------CTTCATGCTGTATGCGGGAAGG 69

MV_589246-1056_54_HMBSrevE08.ab1 GTANNACANACNNNTGCNNCNNTTNTCCAAACCTGACTGATTCTGNNGCCTGNTTNCNGA 660

* ** * ** *

REV_AGM_CON_SEQ_1 AGGTGGGAATTGGTGAGAACAAATGAGATTATATGC-------ACTCNTGTTTATTACCC 122

MV_589246-1056_54_HMBSrevE08.ab1 GCGNNGAGATTCNAGANNNCAATTCTNNCTCTCGAGAACCTGGGCATCTGCCTGGAANNN 720

* * *** ** *** * * * * ** * *

REV_AGM_CON_SEQ_1 CCTCACCCTCCAGCTTTG------------------------------------------ 140

MV_589246-1056_54_HMBSrevE08.ab1 NCTCGCGACGGNGCTTCGGGAGCTGAANTTGACGTACTGGTCGNGCACCGGCCNNCNNAG 780

*** * **** *

REV_AGM_CON_SEQ_1 ----------------------------------------GTACCTGGGCAGGGACATGG 160

MV_589246-1056_54_HMBSrevE08.ab1 GCTTCATTACCCGCTCTTTTTGNNCATGCNNCCAACTTCNCTCCNNCNTGAGGAANANGT 840

* * *** * * *

REV_AGM_CON_SEQ_1 ATGGTAGCCTGC-----------------------ATGGTGTCT--------------TG 183

MV_589246-1056_54_HMBSrevE08.ab1 CTNCTTACTTTCCGTACCNCCGGNCNAAAACGAACCCGTTGTCTGGTCGTAANNNANTTC 900

* * * * * * ***** *

REV_AGM_CON_SEQ_1 TATGCTATCTGAGCCATCTAGACT-CCAGACTCCTCCAGT----------- 222

MV_589246-1056_54_HMBSrevE08.ab1 TCGGATGTNGGATGNCNTAANNCTCCNANACTCCTCNNTGCAGGGTAANNN 951

* * * * ** * ** * * *******

Mosquito 55

REV_AGM_CON_SEQ_1 ------------GTTACGAGCAGTGATGCCTA----------------CCAGCTGTGGGT 32

MV_589246-1057_55_HMBSrevE09.ab1 NNNNNNNNNNNNNNNNNNNGNNNTGNTGTCTCTGTGCTTGNNACTCTATACTCCTGCAGT 60

* ** ** ** * **

REV_AGM_CON_SEQ_1 CATCCTCAGGGCCATCTTCATGCTGTATGCGGGAAGGAGGTGGGAATTGGTGAGAACAAA 92

MV_589246-1057_55_HMBSrevE09.ab1 CATGTAACCTTGTCTTTTTCTNCTGGTTCANGTGGNCTAGTGGNCTTANGCAAGAACCAG 120

*** * ** * *** * * **** * * ***** *

REV_AGM_CON_SEQ_1 TGAGATTATATGCACTC------------------------------------------- 109

MV_589246-1057_55_HMBSrevE09.ab1 GTAANTGTTATGTAAAGCTGTCCCTCTGCACAGAAAANCGGTAGGGAGACTGGCCGNAGT 180

* * **** *

REV_AGM_CON_SEQ_1 ------------------------------------------------------------ 109

MV_589246-1057_55_HMBSrevE09.ab1 TCGGTGCNTGCTCCCTCCTAANGCNACGCAGCGCATGTNCGACCGTCTGTGANTTCCAAG 240

REV_AGM_CON_SEQ_1 ------------------------------------------------------------ 109

MV_589246-1057_55_HMBSrevE09.ab1 ATGGGGAAATGTTTGCCCGNACCCATGGGACACGGATGCCCCCGGTGGCCCTGGTAGCCA 300

REV_AGM_CON_SEQ_1 ------------------------------------------NTGTTTATTACCCCCTCA 127

MV_589246-1057_55_HMBSrevE09.ab1 TGGANGCANGANGAGGTGCACACCCTACCCAGNGGCGTGCNGTNGCTGACCACGCACCCA 360

* * * ** * * **

REV_AGM_CON_SEQ_1 CCCTCCAGCTTTGGTACCTGGGCAGGGACATGGATGGTAGCCTGCATGGT-----GTCTT 182

MV_589246-1057_55_HMBSrevE09.ab1 CCCCGGCCCTGCNGCCCCTGGGNCTGTCAAGGGGGGGGGAACCCCAGGGCCTGCCATAAT 420

*** ** * ****** * * ** ** * ** ** * *

REV_AGM_CON_SEQ_1 GTATG-------------------------------CTATCTGAGCCATCTAGACTCCAG 211

MV_589246-1057_55_HMBSrevE09.ab1 GTATGCCAGTGGCTCTTCAATTACCTGTATGGTGTTTTTCCTGAGGCTTTGAGTTTCCNG 480

***** * ***** * * ** *** *

REV_AGM_CON_SEQ_1 ACTCCTCCAGT------------------------------------- 222

MV_589246-1057_55_HMBSrevE09.ab1 TNNCCNNNCAGNCGNANTTGCTCAGANCNGGGGNATCNNCCNGGAAAA 528

Mosquito 56

REV_AGM_CON_SEQ_1 -----------GTTACGAGCAGTGATGCCTACCAGCTGTGGGTCATCCTCAGGGCCATCT 49

MV_589246-1058_56_HMBSrevE10.ab1 NNNNNNCNNNNNNNNGGNNANGTNAGGTNNNNNCTC-CTCNGCTGCNNATGGGCGNGTCT 59

* ** * * * * * ** ***

REV_AGM_CON_SEQ_1 TCATGCTGTATGCGGGAAGGAGGTGGGAATTGGTGAGAACAAATGAGATTATATGCACTC 109

MV_589246-1058_56_HMBSrevE10.ab1 TCTTTCTCTAGCGGGGGTAACACTGGGGCGGGATGCCCCTTAATTGCGCGAACCACGCTC 119

** * ** ** *** **** * ** *** * * ***

REV_AGM_CON_SEQ_1 NTGTTTATTACCCCCTCACCCTCCAGCTTTGGTACCTGGGCAGGGACATGGATGGTAGCC 169

MV_589246-1058_56_HMBSrevE10.ab1 TACACTCCNAACTCCTCCGGTCAGGTAAATGGGAAATCGCNCCGGNGAAGGTGGAAAGCC 179

* * * **** *** * * * ** * ** * ****

REV_AGM_CON_SEQ_1 TGCATGGTGTCTTGTATGC------------------------TATCTGAGCCATCTAGA 205

MV_589246-1058_56_HMBSrevE10.ab1 GCAAGGGTGACGTGACCATCGACCACGACGAGCAGCCCTTCAAAGCGAACCTCGACAAGA 239

* **** * ** * * ***

REV_AGM_CON_SEQ_1 CTCCAGACTCCTCCAGT------- 222

MV_589246-1058_56_HMBSrevE10.ab1 CTCCAGACTCCTCCAGTCAGGTAA 263

*****************

Mosquito 57

REV_AGM_CON_SEQ_1 ------------------------------------------------------------ 0

MV_589246-1059_57_HMBSrevE11.ab1 NNNNNNNNNCNNTNNCNTNNNNCTCNNTCNCTGTGNNGNNAANTCTATATTCCTGCTGTC 60

REV_AGM_CON_SEQ_1 --------GTTACGAGCAGTGATGCCTACCAGCTGT--GGG----------TCATCCTCA 40

MV_589246-1059_57_HMBSrevE11.ab1 NTGTAACTTTTTCAATGGCTGCTTCTCCTCACNTNNTCCAGCCTTCCCCTCNCTCCTTCC 120

** * * ** * * ** * * * * **

REV_AGM_CON_SEQ_1 GGGCCATCTTCATGCTGTATGCGGGAAGGAGGTGGGAATTGGTGAGAACAAATGAGATTA 100

MV_589246-1059_57_HMBSrevE11.ab1 TGGCAGACTCCAGACTC----CTCCAGTCAGGTAAGNN-----TGNTATCACTCAGATTT 171

*** ** ** ** * * **** * * * * *****

REV_AGM_CON_SEQ_1 TATGCACTCNTGTT---------------TATTACCCCCTCACCCTCCAGCTTT------ 139

MV_589246-1059_57_HMBSrevE11.ab1 CCCGGAGTCAACTNTGTATCCCCTCTCCTCCTTCCCCTCTACCCGTCCGACAGTCTCCCC 231

* * ** * ** *** ** ** *** * *

REV_AGM_CON_SEQ_1 ------------------------------------------------------------ 139

MV_589246-1059_57_HMBSrevE11.ab1 NTCTCATCGNNNAGGTAAGTTTGNAAGCACGCATAGGNCCCGGATGNTTCAGGGGGCCCT 291

REV_AGM_CON_SEQ_1 ------------------------------------------------------------ 139

MV_589246-1059_57_HMBSrevE11.ab1 GATTGCCNCGGCTCNGGAGGAGGANCGCCCTCTTNCCAGTGGTATTGCGNACACTAGGTA 351

REV_AGM_CON_SEQ_1 ---------------------GGTACCTGGGCAGGGAC---------------------- 156

MV_589246-1059_57_HMBSrevE11.ab1 AGGCTCCACCCAGGGCCGAATGCCCCTTGGGCCGTGAGGGGGGGGGGAACCCCAGGGCCT 411

* * ***** * **

REV_AGM_CON_SEQ_1 --------------------ATGGATGGTAGCCTGCATGGTGTCTTGTATGCTATCTGAG 196

MV_589246-1059_57_HMBSrevE11.ab1 GGCTTCATGTAGGCCAGTGCCTCGTGAATTGCCTGTTTGGTGTTTTTC-------CTGAG 464

* * * ***** ****** ** *****

REV_AGM_CON_SEQ_1 CCATCTAGACTCCAGACTCCTCCAGT---------------------------------- 222

MV_589246-1059_57_HMBSrevE11.ab1 CATTTGCGTTTCANTTCCCNNNNNANACGCAANNNNTAAAANCNTGTNNNNNNNCCNGGA 524

* * * ** * *

REV_AGM_CON_SEQ_1 ---- 222

MV_589246-1059_57_HMBSrevE11.ab1 AAAA 528

Mosquito 58

REV_AGM_CON_SEQ_1 ------------------------------------------------------------ 0

MV_589246-1060_58_HMBSrevE12.ab1 NNNNNNNNNNNNNNNNNTTCNNNNCNNACANTTNNNNCNTNCCTGTATGGTCCCGGANAC 60

REV_AGM_CON_SEQ_1 --------------------------------------------------------GTTA 4

MV_589246-1060_58_HMBSrevE12.ab1 TANNCTGTTNNNNANNGCTCCNGGCTCCTCCTGCCGACTAANCNNANNNGACNACTGNTN 120

* *

REV_AGM_CON_SEQ_1 CGAGCAGTGAT--GCCTACCAGCTGTGGGTCATCCTCAGGG---CCATCTTCATG----C 55

MV_589246-1060_58_HMBSrevE12.ab1 NGNCNAGAGATCCGCCTCCTGGGTAANGGTGAGTCCCACTGTGGAACNAGCCGTCCNGTG 180

* ** *** **** * * * *** * * ** * * *

REV_AGM_CON_SEQ_1 TGTATGCGGGAAGGAGGTGGGAATTGGTGAGAAC-AA----ATGAGATTATATGC----- 105

MV_589246-1060_58_HMBSrevE12.ab1 TGNATGAAGGGTCGACGTTGCCTTTTNNAGGGAGGANCTNTANGACACTGNATGCTTTGA 240

** *** ** ** ** * ** * * * * ** * * ****

REV_AGM_CON_SEQ_1 --ACTCNTGTTTATTACCCCCTCACCCTCCAGCTTTGGTACCTGGGCAGGGACATGGATG 163

MV_589246-1060_58_HMBSrevE12.ab1 AANTTCCAATTTCTTGTGACATGATGTCCNTGCTTTTAACAC-CGCCGNNAATGCNTGCN 299

** *** ** * * * * ***** * * * *

REV_AGM_CON_SEQ_1 GTAGCCTG---CATGGTGTCTTGTAT----GCTATCTGAGCCATCTAGACTCCAGACTCC 216

MV_589246-1060_58_HMBSrevE12.ab1 GTGCCATGTCGGACNCTTTCTCTTGTGCTNCNNAGCTCTCCAGTTCAGTCACTATACTGA 359

** * ** * * *** * * * ** * * ** * * * ***

REV_AGM_CON_SEQ_1 TCCAGT------------------------------------------------------ 222

MV_589246-1060_58_HMBSrevE12.ab1 NAGAGTCTGTGATAAGCCCTCACTTTATCTTNCATGTCTNTCTTNANGAGGGATCACNAT 419

***

REV_AGM_CON_SEQ_1 ------------------------------------------------------------ 222

MV_589246-1060_58_HMBSrevE12.ab1 GGANCTGGGAAGCNNNGNGNNNNANTCNNNCGGATAGGTNCNTGGNGGNCTNNANNCNNN 479

REV_AGM_CON_SEQ_1 --------------------------------------- 222

MV_589246-1060_58_HMBSrevE12.ab1 ANGCTTCTNGGCGNGANNNACTCCTCCAGTCAGGTAANN 518

Mosquito 59

REV_AGM_CON_SEQ_1 GTTACGAGCAGTGATGCCTACCAGCTGTGGGTCATCCTCAGGGCCATCTTCATGCTGTAT 60

MV_589246-1061_59_HMBSrevF01.ab1 -----NN-------------NNNNNNNNNNNNNNNNNNNNNNNNNNNNNNNGACGGNNCT 42

*

REV_AGM_CON_SEQ_1 GCGGGAAGGAGGTGGGAATTGGTGAGAACAAATGAGATTATATGCACTCNTGTTTATTAC 120

MV_589246-1061_59_HMBSrevF01.ab1 GCTGGACCGTGGNGTGACTCCNACTCCTCC----------T---GTCNGGTAAATATTCC 89

** *** * ** * ** * * * * * **** *

REV_AGM_CON_SEQ_1 CCCCTCACCCTCCAGCTTTGGTACCTGGGCAGGGACATGGATGGTAGCCTGCATGGTGTC 180

MV_589246-1061_59_HMBSrevF01.ab1 TCCGGTCAGGNAANGATTACTTTTCTCCA-GCAGACTCTTCTAGT----ACTCTGGAGTG 144

** * ** * ** *** * ** *** **

REV_AGM_CON_SEQ_1 TTGTATGCTATCTGAGCCATCTAGACTCCAGACTCCTCCAGT---------- 222

MV_589246-1061_59_HMBSrevF01.ab1 GTGTGACTCATCTCTCAGTACCAGACTCCAGACTCCTCCAGTCAGGTAANGG 196

*** **** * ********************

Mosquito 60

REV_AGM_CON_SEQ_1 ----------------------GTTACGAGCAGTGATGCCTACCAGCTGTGGGTCATCC- 37

MV_589246-1062_60_HMBSrevF02.ab1 NNNNNNNNNNNGNAGNNTNNGNNCTNANAGNTANGNNGANNAGCTGNTNCGGNACATAAN 60

* ** * * * * * * ** ***

REV_AGM_CON_SEQ_1 -----TCAGGGCCATCTTCATGCTGTATGC------------------------------ 62

MV_589246-1062_60_HMBSrevF02.ab1 CNGNAACAGGCNTATCTTTATNNAGAATCTGTCAATGGGATANCCTTGATGGTTGCAACA 120

**** ***** ** * **

REV_AGM_CON_SEQ_1 ------------------------------------------------------------ 62

MV_589246-1062_60_HMBSrevF02.ab1 CATAGTGTGATTCTCATCAGCTTCTGCAACATGAGCTGTGGGAGCAAACATCCTCATTGG 180

REV_AGM_CON_SEQ_1 ------------------------------------------------------------ 62

MV_589246-1062_60_HMBSrevF02.ab1 CTGGTGGAGAGGTGGAGAACGATCCACCTCTCCAAACCTCCTCTGATCTGGCTACTACCA 240

REV_AGM_CON_SEQ_1 -----------------------------------------------------------G 63

MV_589246-1062_60_HMBSrevF02.ab1 GCAGCCATGGACTAGACTCCAGACTCCTCCAGTCAGGTAATGGACTTCAAGCTGCTTTGC 300

REV_AGM_CON_SEQ_1 GGAAGGAGGTGGGAATTGGTGAGAACAAATGAGATTATATGCACTCNTGTTT-ATTACC- 121

MV_589246-1062_60_HMBSrevF02.ab1 CGCTGCAGGTTTAGAGTAGAGTTGACAATCTTCATTTGATTCTCCAGTGCAGCAATGCCA 360

* * **** * * * * **** *** ** * * ** * * **

REV_AGM_CON_SEQ_1 ---------CCCTCACCCTCCAGCTTTGGTACCTGGGCAGGGACATGGATGGTAGCCTGC 172

MV_589246-1062_60_HMBSrevF02.ab1 GAGAAGCCAGCGGCTGCGGCCGGCAATTCTGACTGTGCATCGAATGGGGTAGGAGGCTTC 420

* * * ** ** * * *** *** ** ** * * ** ** *

REV_AGM_CON_SEQ_1 ATGGTGTCTTGTATGCTATCTGAGCCATCTAGACTCCAGACTCCTCCAGT---------- 222

MV_589246-1062_60_HMBSrevF02.ab1 GAGGAGCTCCTGG-------GGCCATCCTGAGACTCCAGACTCCTCCAGTCAGGTAATGG 473

** * * ********************

REV_AGM_CON_SEQ_1 ------------------------------------------------------------ 222

MV_589246-1062_60_HMBSrevF02.ab1 AGCTCNCNAGNGGTCTGATATTCNNNGNTAGNCACCTTTGGTCNAGGACGCCAAACANAG 533

REV_AGM_CON_SEQ_1 ------------------------------------------------------------ 222

MV_589246-1062_60_HMBSrevF02.ab1 CTNAGTCAGANCATACTANCAANCATTNAANTCCCCCTACCTTTGCNNCTNCTGTACTTA 593

REV_AGM_CON_SEQ_1 ------------------------------------------------------------ 222

MV_589246-1062_60_HMBSrevF02.ab1 TGGGTATGATACATGNGCTGACCNNCCCGGTNNATGAANAGGTTGCATTGGNTGCCCNTA 653

REV_AGM_CON_SEQ_1 ------------------------------------------------------------ 222

MV_589246-1062_60_HMBSrevF02.ab1 CAAACAAATGANGGAGNNTNNGTNTGACTACCTTCCTGCTATATNCTTTGAAGGAAAGGA 713

REV_AGM_CON_SEQ_1 ------------------------------------------------------------ 222

MV_589246-1062_60_HMBSrevF02.ab1 AAGACTTACTCANAGTCCGGCACACGAGCAANTTGNTGTGCATGTATGTTGAGGNTGAGG 773

REV_AGM_CON_SEQ_1 --- 222

MV_589246-1062_60_HMBSrevF02.ab1 NTN 776

Mosquito 62

REV_AGM_CON_SEQ_1 -----GTTACG-------AGCAGTGATGCCTACC--------AGCTGTGGGTCATCCTCA 40

MV_589246-1064_62_HMBSrevF04.ab1 NNNNNNNNNNGNNNNNNNNTNNCTGCTGACTNTCAGANNNCTCGATTNNGGTAACGCCCA 60

* ** ** ** * * * *** * * **

REV_AGM_CON_SEQ_1 GGGCCATC-----------TTCATGCTGT-----------------ATGCGGGAAGGAG- 71

MV_589246-1064_62_HMBSrevF04.ab1 GGACGACGAAGTCGGCTTTTGCACGCGACATCGGAACCGACTCACCAAACGCGAACGACG 120

** * * * ** ** * ** *** **

REV_AGM_CON_SEQ_1 -------------------GTGGGAATTGGTGAGAACAAATGAGATTATATGCACTCNTG 112

MV_589246-1064_62_HMBSrevF04.ab1 CATCGGCTCGAGCGAGGCTCGGTCCGTCGTTCAAGCCGTCACCGATCATGGCCACGCGCC 180

* * * * * * *** ** *** *

REV_AGM_CON_SEQ_1 TT---------------------------------------------------------- 114

MV_589246-1064_62_HMBSrevF04.ab1 TGCCCTCGGCCTGTAGACTCCAGACTCCTCCAGTCAGGTAAANNCGGACTCGTCCAGTNT 240

*

REV_AGM_CON_SEQ_1 ---------TATTACCCCCTCACCCTCCAGCTTTGGTACCTGGGCAGGGACATGGATGGT 165

MV_589246-1064_62_HMBSrevF04.ab1 TTGGNTNCTGAGTTCGCACTGANCGTCATGCTCCC--AACANNANATGGACTCGGACTCT 298

* * * * ** * * ** *** * * * **** *** *

REV_AGM_CON_SEQ_1 AGCCTGCATGGTGTCTTGTATGCTATCTGAGCCATCTAGACTCCAGACTCCTCCAGT--- 222

MV_589246-1064_62_HMBSrevF04.ab1 ATATTGCCGT---GTTCGCNCGGTGAATGTTCACTGCANACTCCAGACTCCTCCAGTCAG 355

* *** * * * * ** * * * ******************

REV_AGM_CON_SEQ_1 ---------------------------------- 222

MV_589246-1064_62_HMBSrevF04.ab1 GTAANCCGNGGGTTTCTGGGGCANANNCTGTGNN 389

Mosquito 63

REV_AGM_CON_SEQ_1 --------------------------------------------GTTACGAGCAGTGATG 16

MV_589246-1065_63_HMBSrevF05.ab1 NNNNNNNNNNNNCNCNNNNNCANCCATTTGTAACTGTNNTATACCTATACNATAGTGATG 60

* *******

REV_AGM_CON_SEQ_1 CCTACCAGCTGTGGGTCATCCTCAGGGCCAT----------------------------- 47

MV_589246-1065_63_HMBSrevF05.ab1 TGTGCACGAAGATCGATTGTCTTCGTGCGATGTTCTACCACATGGTAGAAATATTGCTCT 120

* * * * * ** * ** **

REV_AGM_CON_SEQ_1 ------------------------------------------------------------ 47

MV_589246-1065_63_HMBSrevF05.ab1 TTATCTGTGATATCAATAAACGACTAGGAGTAGTTCATGTTCAGACTCCAGACTCCTCCA 180

REV_AGM_CON_SEQ_1 ---------------------------------------CTTCATGCTGTATGCGGGAAG 68

MV_589246-1065_63_HMBSrevF05.ab1 GTAAGGTAATTCTTCTCTCGGCACGNTCCGCTAGACTCCCTTCTTCCCCGATCCTGGAAN 240

**** * * ** * ****

REV_AGM_CON_SEQ_1 GAGGT------------------------------GGGAATT--------------GGTG 84

MV_589246-1065_63_HMBSrevF05.ab1 GCCTTCNACATTNCCGCTCCTTGCGCNTCCACANTNCGNCTTTCTGGTAAGGAACTGTTG 300

* * * ** * **

REV_AGM_CON_SEQ_1 AGAACAAA-TGAGATTA---------------------------------------TATG 104

MV_589246-1065_63_HMBSrevF05.ab1 ACCANAATNTCNACTTCTNTGGAANGGCTACAANTCCTCCAGTCAGGAAATNATCTTTCA 360

* * ** * ** *

REV_AGM_CON_SEQ_1 CACTCNTGTTTATTACCCCCTCACCCTCCAGCTTTGGTACCTGGGCAGGGACATGGATGG 164

MV_589246-1065_63_HMBSrevF05.ab1 AGCTNGTGGTTAANGTCCGCCGCCATGGCNATTCTGCTGCCTACNNNCNAACATCGGTCT 420

** ** *** ** * * * * ** * *** **** * *

REV_AGM_CON_SEQ_1 TAGCCTGCATGGTGTCTTGTATGCTATC-------------------------------- 192

MV_589246-1065_63_HMBSrevF05.ab1 ACTCGAAGACGTTAACTCACTTCCGGACTCCTCCNNTCATGTAAAGCTACNCNNACCTGG 480

* * * * ** * * *

REV_AGM_CON_SEQ_1 ------------------------------------------------------------ 192

MV_589246-1065_63_HMBSrevF05.ab1 GTGCCAAAACGTANCAAACGCTTCATCCCCGCGNGGNTTAGCNCCTCNATCACTTTGTTC 540

REV_AGM_CON_SEQ_1 -------------------------TGAGCCATCTAGACTCCAGACTCCTCCAGT----- 222

MV_589246-1065_63_HMBSrevF05.ab1 TGCCGNAAGATTTTCCGGATCNNNNNTCNNATGANCGACTCNNNACTCCTCCGGTCGCGT 600

***** ******** **

REV_AGM_CON_SEQ_1 ------------------------- 222

MV_589246-1065_63_HMBSrevF05.ab1 ANAGAGTCNCTNTNGATTTTTCNNN 625

Mosquito 64

REV_AGM_CON_SEQ_1 --------------------GTTACGAGCAGTGATGCCTACCAGCTGTGGGTCATCCTCA 40

MV_589246-1066_64_HMBSrevF06.ab1 NNNNNNNNNNTNNNGNNANGTTACNGAGCNNNGNTGCCTACCNNCTGTGGGNNAACCTCA 60

* **** * ******** ******* * *****

REV_AGM_CON_SEQ_1 GGGCCATCTTCATGCTGTATGCGGGAAGGAGGTGGGAATTGGTGAGAACAAATGAGATTA 100

MV_589246-1066_64_HMBSrevF06.ab1 GGGCCATCTTCATGCTGTATGAGGGAAGGAGGTGGGATTTGGTGAGAACA--AGAGATTA 118

********************* *************** ************ *******

REV_AGM_CON_SEQ_1 TATGCACTCNTGTTTATTACCCCCTCACCCTCCAGCTTTGGTACCTGGGCAGGGACATGG 160

MV_589246-1066_64_HMBSrevF06.ab1 TATGCACTCTTGTTTATTACCCCCTCGCCCTCCAGCTTTGGTACCTGGGCAGGGACATGG 178

********* **************** *********************************

REV_AGM_CON_SEQ_1 ATGGTAGCCTGCATGGTGTCTTGTATGCTATCTGAGCCATCTAGACTCCAGACTCCTCCA 220

MV_589246-1066_64_HMBSrevF06.ab1 ATGGTAGCCTGCATGGTCTCTTGTATGCTATCTGAGCCGTCTAGACTCCAGACTCCTCCA 238

***************** ******************** *********************

REV_AGM_CON_SEQ_1 GT---------------------------------------------------------- 222

MV_589246-1066_64_HMBSrevF06.ab1 GTCAGGTAANCCCGGGTTGAAGGCGACCACCCTGCTGTGCGNTANTGCGCGGGACATCCA 298

**

REV_AGM_CON_SEQ_1 ------------------------------------------------------------ 222

MV_589246-1066_64_HMBSrevF06.ab1 CTCGAACATTCTATTCCCCNGTTTGCNGGGGACGCTTNGNGCGTCGATGATCCCGAGAGT 358

REV_AGM_CON_SEQ_1 ------------------------------------------------------------ 222

MV_589246-1066_64_HMBSrevF06.ab1 GNNNCCCGGCTCGTGGATGATGTGCCCCGGAAATCATTGAGCGATTGCGTCCCCAACTNA 418

REV_AGM_CON_SEQ_1 ------------------------------------------------------------ 222

MV_589246-1066_64_HMBSrevF06.ab1 ATACGNACTAGCCCCNNGCGCACCCACCGGCGGATCGGTGTGCAGCCAATGGATCACGTT 478

REV_AGM_CON_SEQ_1 -------------------------------------------------------- 222

MV_589246-1066_64_HMBSrevF06.ab1 NCAGTGGTCATGTCNGTGGNTGTGCTATCAAACTGTGGGTATCCNTGTGANANNTT 534

Mosquito 65

REV_AGM_CON_SEQ_1 ------------------GTTACGAGCAGTGATGCCTACCAGCTGTGGGTCATCCTCAGG 42

MV_589246-1067_65_HMBSrevF07.ab1 NNNNNNNNNNNNNNNNANGNNANNAGCGNTGATGCNNN-NNNNTGNGGGTGTTCTTCAGG 59

* * *** ****** ** **** ** *****

REV_AGM_CON_SEQ_1 GCCATCTTCATGCTGTATGCGGGAAGGAGGTGGGAATTGGTGAGAACAAATGAGATTATA 102

MV_589246-1067_65_HMBSrevF07.ab1 GGATCTTCT--GC-NGNAGANGGAAGGAGGTGGGAT----GATGCGACAACCNANANNTA 112

* * ** * ************** * ** **

REV_AGM_CON_SEQ_1 TGCACTCNTGTTTATTACCCCCTCACCCTCCAGCTTTGGTACCTGGGCAGGGACATGGAT 162

MV_589246-1067_65_HMBSrevF07.ab1 GGCACTCTTGTTTATTACCCCCTCACC--CTCNCCTTTGGGCCTGGGCAGGGACATGGAT 170

****** ******************* * * ** * *******************

REV_AGM_CON_SEQ_1 GGTAGCCTGCATGGTGTCTTGTATGCTATCTGAGCCATCTAGACTCCAGACTCCTCCAGT 222

MV_589246-1067_65_HMBSrevF07.ab1 GGTAGCCTGCATGGTCTCTTGTATGCTATCTGAACCGGCTAGACACCNGACTCCTCCAGT 230

*************** ***************** ** ****** ** ************

REV_AGM_CON_SEQ_1 ------------------------------------------------------------ 222

MV_589246-1067_65_HMBSrevF07.ab1 CAGGTAATNNGCTGTATCACTNTCCTTGNTCNTGCTTACTTNNCCCCTTCNCTTGCTGTT 290

REV_AGM_CON_SEQ_1 ------------------------------------------------------------ 222

MV_589246-1067_65_HMBSrevF07.ab1 TTTACCCANGCNNCCGTTGGAACTCCAAGNCTAGNNANTTCAGGATNCNTTAAACCTTCT 350

REV_AGM_CON_SEQ_1 ------------------------------------------------------------ 222

MV_589246-1067_65_HMBSrevF07.ab1 TNAANCNTTGTTTTNATGTCTTCATTTCACTATGCTNGGACGNTNCNATTTCCCNGANGC 410

REV_AGM_CON_SEQ_1 ------------------------------------------------------------ 222

MV_589246-1067_65_HMBSrevF07.ab1 TNGACACCCGATANCTCAAGACTCCTCCCGGCCTGCAANCTGATGGATNGNNAACATTTN 470

REV_AGM_CON_SEQ_1 ------------------------------------------------------------ 222

MV_589246-1067_65_HMBSrevF07.ab1 TNGATTGCCTTNCNTTCCATCNCTTCATCCCCCGGGNNCTGGAANNNCCNTCACTTTGTT 530

REV_AGM_CON_SEQ_1 ------------------------------------------------------------ 222

MV_589246-1067_65_HMBSrevF07.ab1 CTGACNNATGATNTTCCTGATCATCGTCTCTTGATCATCGACTCANTACTCNTCAGGGTG 590

REV_AGM_CON_SEQ_1 ------------------------------------------------------------ 222

MV_589246-1067_65_HMBSrevF07.ab1 CGTAAGGAGCACGAAATTTGACATAATTATCGTGCGCCGTCACTAGGCTTCTCAACAGCA 650

REV_AGM_CON_SEQ_1 ------------------------------------------------------------ 222

MV_589246-1067_65_HMBSrevF07.ab1 ACCANAATNTTGCCGCNTCGTCNNNATCACGCCATCTTTTGAGGAAGAGGTCTTCGTACT 710

REV_AGM_CON_SEQ_1 ------------------------------------------------------------ 222

MV_589246-1067_65_HMBSrevF07.ab1 TCCGGTACCAGTTGTCTANATCTTANACGAATTCNAATANATGATGGATGCCAGAGATTC 770

REV_AGM_CON_SEQ_1 ----------------------------- 222

MV_589246-1067_65_HMBSrevF07.ab1 GTANACTCCAACTCCTCCNGTCAGGGAAA 799

Mosquito 67

REV_AGM_CON_SEQ_1 -------------------------------------------------GTTACGAGCAG 11

MV_589246-1069_67_HMBSrevF09.ab1 NNNNNNNNNNNNNNNNNNCCTANNGNCNNTNTNNTGACTCGTAGTATACCTATACNATAG 60

* **

REV_AGM_CON_SEQ_1 TGATGCCTACCAG----------------------------------------------- 24

MV_589246-1069_67_HMBSrevF09.ab1 TAATGTGTGCCGAAGATCGATTGTCTTCGTGCGATGTTCTACCACATGGTAGAAATATTG 120

* *** * **

REV_AGM_CON_SEQ_1 -------------------------------CTGTGGGTCATCCTCA------GGGCCAT 47

MV_589246-1069_67_HMBSrevF09.ab1 CTCTTTATCTGTGATATCAATAAACGACTAGGAGTAGTTCATGTTCAGACTCCAGACTCC 180

** * **** *** * *

REV_AGM_CON_SEQ_1 CTTCATGCTGTATGCGG---GAAGGAG--------------------------------- 71

MV_589246-1069_67_HMBSrevF09.ab1 TCCANTCAGGTAACCTGAANGCCGGATNNCTNCNTGGGACTCCTGACCAAATCCCAANTG 240

* *** * * * ***

REV_AGM_CON_SEQ_1 ----------------------------------------GTGGGAATTGGTGAGAACAA 91

MV_589246-1069_67_HMBSrevF09.ab1 AATTNCCTTTCAAGTCGCAATTCCGTTCTTGAAGATCCCCAAACTGAAACGTGGGAATGA 300

* *** *** *

REV_AGM_CON_SEQ_1 ATGAGATTATATGCACTCNTGTTTATTACCCCCTCACCCTCCAGCTTTGGTACCTGGGCA 151

MV_589246-1069_67_HMBSrevF09.ab1 NTGGCAATATGCCCCGTTTAAGCTGTAAGCGT--CNCACCCAGACCTCGATGGATGCGTT 358

** * *** * * * * * * * * * * * * * * ** *

REV_AGM_CON_SEQ_1 GGGACATGG---ATGGTAGCCTGCATGGTGTC---------TTGTATGCTATCTGAGCCA 199

MV_589246-1069_67_HMBSrevF09.ab1 GGCGACTGTGAGAACGGACATNNTTTGGTGTTACGGCTTGTNGTTCTCCAATCTGNNACT 418

** ** * * * ****** * * * ***** *

REV_AGM_CON_SEQ_1 TCTAGACTC--------------------------------------------------- 208

MV_589246-1069_67_HMBSrevF09.ab1 CCTNANNTCCAGGAANNGCAAACTTCCTCCGGGGCTGCCTGCGGCTCGTTTGNNAACCTT 478

** **

REV_AGM_CON_SEQ_1 ------------------------------------------------------------ 208

MV_589246-1069_67_HMBSrevF09.ab1 GACATGATGGCCANNACCTTGCANANANCTTCATCCCTCGGGCGNNNNNCTCGNNNNTCA 538

REV_AGM_CON_SEQ_1 --------------------------------------------------CAGACTCCTC 218

MV_589246-1069_67_HMBSrevF09.ab1 CTTAATNCTGCCGATGATTTTCNGCATCAANGCTTCTNGATGANNGACGCCANACTCCTC 598

** *******

REV_AGM_CON_SEQ_1 CAGT-------------------------------------------------------- 222

MV_589246-1069_67_HMBSrevF09.ab1 GAGNGTGGTAGAATCGNCTGCGTCNNTCTGCGTGANCTGGAAGCTCTGTTACCGTTNGCT 658

**

REV_AGM_CON_SEQ_1 ------------------------------------------------------------ 222

MV_589246-1069_67_HMBSrevF09.ab1 GNGCNNGGANATNCGGACNCNGATACCTCTTTCAGCTTCTTCTCCATNTCATCGAACGAN 718

REV_AGM_CON_SEQ_1 ------------------------------------------------------------ 222

MV_589246-1069_67_HMBSrevF09.ab1 TACTCCGATGNNGCTTCNGGAGCTGAAGTTGACTTACTTGACNTGCNCCGTCNCGCTGAG 778

REV_AGM_CON_SEQ_1 ------------------------------------------------------------ 222

MV_589246-1069_67_HMBSrevF09.ab1 GCTTCTTACCGACTNTTGCGCNTCGTNNACTTCNCTCCGTCTGAGAGAGGTCTTCGTACT 838

REV_AGM_CON_SEQ_1 ------------------------------------------------------------ 222

MV_589246-1069_67_HMBSrevF09.ab1 TCNGTACCACGGTCGAAACNANCCGTTGTCNGATCNNNNACATTNCCNGGATNGNTNGAN 898

Mosquito 68

REV_AGM_CON_SEQ_1 -----------------GTTACGAGCAGTGATGCCTACCAGCTGTGGGTCATCCTCAGGG 43

MV_589246-1070_68_HMBSrevF10.ab1 NNNNNNNNNNNGNNNGANGNNNNANTANTGATGCGTNNNCNNNNNNGCTTTNN--TGACG 58

* * ****** * * * *

REV_AGM_CON_SEQ_1 CCATCTTCATGCTGTATGCGGGAAGGAGGTGGGAATTGGTGAGAACAAATGAGATTATAT 103

MV_589246-1070_68_HMBSrevF10.ab1 GTGGCGCGCTGGGACATCANGGANGCCTGTGGGATTTGCCCCG---CTTAGAGATTATAT 115

* ** ** *** * ****** *** * **********

REV_AGM_CON_SEQ_1 GCACTCNTGTTTATTACCCCCTCACCCTCCAGCTTTGGTACCTGGGCAGGGACATGGATG 163

MV_589246-1070_68_HMBSrevF10.ab1 TCNTATTTGTTTGTTG----GGGTNCACCCTACTCCCAATCTGGTGGTTNGAATGGGATG 171

* ***** ** * ** ** * * * ** *****

REV_AGM_CON_SEQ_1 GTAGCCTGCATGGTGTCTTGTATGCTATCTGAGCCATCTAGACTCCAGACTCCTCCAGT- 222

MV_589246-1070_68_HMBSrevF10.ab1 GTTCCCTGCATGGAATCCTGTATGCTATCTGANACTCCTAAACCCCCNACTCCTGNAATC 231

** ********* ** ************** * *** ** ** ****** * *

REV_AGM_CON_SEQ_1 ------------------------------------------------------------ 222

MV_589246-1070_68_HMBSrevF10.ab1 AGGTAACCTNCCACTCTCTNACAGGATTTNTCGNACCATGAAACTCTGTTCTGTTGTTTG 291

REV_AGM_CON_SEQ_1 ------------------------------------------------------------ 222

MV_589246-1070_68_HMBSrevF10.ab1 NNCATAGTCNGANNNATNTTGATGATGACTTGCCNATAGCTCGTGTATTNCAGACTCCAG 351

REV_AGM_CON_SEQ_1 ------------------------------------------------------------ 222

MV_589246-1070_68_HMBSrevF10.ab1 ACTCCTCCGGTCAGGAAAACTNNNTGGTAAAAGATGCGGGGTCCCTCCGACCNGCCAGAC 411

REV_AGM_CON_SEQ_1 ------------------------------------------------------------ 222

MV_589246-1070_68_HMBSrevF10.ab1 TTCCTAGATCANGATAAAATTCATTCTNCAGGCGAGANGGCCNCCGATGATGGGCAAGTT 471

REV_AGM_CON_SEQ_1 ------------------------------------------------------------ 222

MV_589246-1070_68_HMBSrevF10.ab1 GGTGCAAGGGAANANGCGTGNNCATACNCTCCAAACCCCCCGGGGAAAGTAANGCTTTTG 531

REV_AGM_CON_SEQ_1 ------------------------------------------------------------ 222

MV_589246-1070_68_HMBSrevF10.ab1 AATTGTTCCGCCGAGATCTTGCTGAGCTCTAAGTCTTCACCTCATCGGTNGGNANTCCCA 591

REV_AGM_CON_SEQ_1 ------------------------------------------------------------ 222

MV_589246-1070_68_HMBSrevF10.ab1 NCNNNCGTTANGGAATCNTCNGCGTCATTCTTCATGAGCTGGAAGCACTGNNNCNNTTTG 651

REV_AGM_CON_SEQ_1 ------------------------------------------------------------ 222

MV_589246-1070_68_HMBSrevF10.ab1 CTGAACAGGNANATTCGGACNCTGAATANCTCTTTCNCCTTCTTCNNNNTTNNNTCGAAC 711

REV_AGM_CON_SEQ_1 ------------------------------------------------------------ 222

MV_589246-1070_68_HMBSrevF10.ab1 GAGNACTCNNNATGNNGCTTCGGGAGCTNANNTNGANNTACTTGTCGAGCNCCNGTCACG 771

REV_AGM_CON_SEQ_1 ------------------------------------------------------------ 222

MV_589246-1070_68_HMBSrevF10.ab1 ATNAGGNTTCTTNACCGCNCTTTTTNNCCANNNTACNACTTCGCTCCGTCCTTGAGGANN 831

REV_AGM_CON_SEQ_1 ------------------------------------------------------------ 222

MV_589246-1070_68_HMBSrevF10.ab1 ANGTCTTCGTACTTTCGGTACCACCGGTNNAAACNANCCCGTTGCCGGGANCGTANACCA 891

REV_AGM_CON_SEQ_1 ------------------------------------------------- 222

MV_589246-1070_68_HMBSrevF10.ab1 NTTCCGGANGTTGGATGCCNAANACTCCNNNCTCCTCCNNGTCAGGNAA 94

Mosquito 69

REV_AGM_CON_SEQ_1 ------------------------------------------------------------ 0

MV_589246-1071_69_HMBSrevF11.ab1 NNNNNNNNTNNNNNNNNNNNNANNNNNGACTTTTNTNNTGNANNNTNTCCGGTCTGNNCT 60

REV_AGM_CON_SEQ_1 ----GTTACGAGCAGTGATGCCTACCAGCTGTGGGTCATCCT------------------ 38

MV_589246-1071_69_HMBSrevF11.ab1 ATGCGTGACGATCATCCCTTGTTTTNGGGCGAGGTTCANCCGCCTGGTANAGTTATTGCC 120

** **** ** * * * * ** *** **

REV_AGM_CON_SEQ_1 ------CAGGGCCATCTTC----------------------------------------- 51

MV_589246-1071_69_HMBSrevF11.ab1 AATTATCTGCGAGATGTCCTTGTTGACTCGAGTACTGCACTCTCAGACTCGCAAGACCTC 180

* * * ** * *

REV_AGM_CON_SEQ_1 ------------------------------------------------------------ 51

MV_589246-1071_69_HMBSrevF11.ab1 CCCACAAGTAAAACGGTAACAGACGTTAGAGTCCAGACTCCTCCAGTCAGGTAANAGGNA 240

REV_AGM_CON_SEQ_1 ------ATGCTGTATGCGGGAAGGAGGTGGGAATTGGTGAGAACAAATGAGATTATATGC 105

MV_589246-1071_69_HMBSrevF11.ab1 ATCGNTCTTGNCTCCNGGGAAANGTGTGGGGTGTNCGGGACTATGACTGTCACTCCATGA 300

* * ** ** * * *** * * ** * * ** * * ***

REV_AGM_CON_SEQ_1 ACTCNTGTTTATTACCCCCTCACCCTCCAGCTTTGGTACCTGGGCAGGGACATGGATGGT 165

MV_589246-1071_69_HMBSrevF11.ab1 CTT---------------CGACCCCTTCCGAACAGGTAGACCCGCANCCTCAGGGGTCNN 345

* * **** * * **** *** ** ** *

REV_AGM_CON_SEQ_1 A------GCCTGCATGGTGTCTTGTATGCTATCT-------------------------- 193

MV_589246-1071_69_HMBSrevF11.ab1 ATGCNCATTCTTGAGNGTCGCTTGTCCCTCTTCTCCCCNCTGGAGTANNCCCCTTTTTAN 405

* ** * ** ***** ***

REV_AGM_CON_SEQ_1 ------------------------------------------------------------ 193

MV_589246-1071_69_HMBSrevF11.ab1 GCCATANAGAGTTATACCCNGNNAGTCGTCACTCANNCNGGATNGNAAGCTAAGGTATCA 465

REV_AGM_CON_SEQ_1 --------------------------------------------GAGCCATCTAGACTCC 209

MV_589246-1071_69_HMBSrevF11.ab1 TTTGGAACGGCTGGNCNAATCATTTGCGTATTGNTGATCGACACGCCGGTAATAGACTCC 525

* ********

REV_AGM_CON_SEQ_1 AGACTCCTCCAGT----------------------------------------------- 222

MV_589246-1071_69_HMBSrevF11.ab1 NNACTCCTCCTGTCAGGTAANGGAGCTCNNGCAGCTGATCACCCGGCATCANCGACCANN 585

******** **

REV_AGM_CON_SEQ_1 ---------------------------------------- 222

MV_589246-1071_69_HMBSrevF11.ab1 ACTCCCNAGGGNGNNNTAAGGAGCACCAANTTGANATNGN 625

Mosquito 70

REV_AGM_CON_SEQ_1 ------------------------------------------------------------ 0

MV_589246-1072_70_HMBSrevF12.ab1 NNNNNNCNNAGNTNTCNTNNNCTGNNNACTCNNNACNNCCTCCTCTCAGGTAAGACGTAA 60

REV_AGM_CON_SEQ_1 ----------GTTACGAGCAGTGATGCCTACCAGCTGTG-GGTCATCCTCAGGGCCATCT 49

MV_589246-1072_70_HMBSrevF12.ab1 CTTCGATACACTTTANCAATGTGATAATG-GCAGCTGTTGATTACTTTTCTCCAACATAC 119

** ***** ******* * * ** ***

REV_AGM_CON_SEQ_1 TCATGCTGTATGCGGGAAGGAGGTGGGA-ATTGGTGAGAACAA--ATGAGATTATATGCA 106

MV_589246-1072_70_HMBSrevF12.ab1 TCTTCTACTACTCTGGAGTGGTGTGACTCATCTCTCAGTACCAGACTCCAGACTCCTCCA 179

** * ** * *** * *** ** * ** ** * * * **

REV_AGM_CON_SEQ_1 CTCNTGTTTATTACCCCCTCAC-------------------------------------- 128

MV_589246-1072_70_HMBSrevF12.ab1 GTCAGGTAAACTCCAGACTCCTCCAGTCAGGTAAGTNCTCGAGGGGTCTGGNACNCCAAA 239

** ** * * * ***

REV_AGM_CON_SEQ_1 -CCTCCAGCTTTGGTACCT-----------------GGGCAGGGACATGGATGGTAGCCT 170

MV_589246-1072_70_HMBSrevF12.ab1 GTNTGGATCTGCGGTAANTGTTGACTCGTCCGGGCGGGTAAGAGGCAAGTGNCGAAGTAG 299

* * ** **** * ** ** * ** * * **

REV_AGM_CON_SEQ_1 GCATGGTGTCT-----------TGTATGCTATCTGAGCCATCTAGACTCCAGACTCCTCC 219

MV_589246-1072_70_HMBSrevF12.ab1 NGGTGGGTTTTTGAGGANAGGGACGCNCTGNTGANANCCGTCCAGACTCNANACTCCTCC 359

*** * * * * ** ** ****** * ********

REV_AGM_CON_SEQ_1 AGT-------- 222

MV_589246-1072_70_HMBSrevF12.ab1 NNACNNGTAAN 37

Mosquito 71

REV_AGM_CON_SEQ_1 ------------------------------------------------------------ 0

MV_589246-1073_71_HMBSrevG01.ab1 NNNNNNNNNNNNGTNNNNNNTNNNNNNCGNAAATTNNNCNNNTNNTCTGTAGGGTCNNGT 60

REV_AGM_CON_SEQ_1 -----GTTACGAGCAGTGATGCCT-----------------------------------A 20

MV_589246-1073_71_HMBSrevG01.ab1 AATNNGTTACGTGNAAGGAGGCTCANGGCTCCTCCACCCCACGAATACTATCGAAGTTAA 120

****** * * ** ** *

REV_AGM_CON_SEQ_1 CCAGCT-----GTGGGTCATCCTCAGGGCCATCTTC-ATGCTGTATGCGGGAAGGA---- 70

MV_589246-1073_71_HMBSrevG01.ab1 CCGGCTCNGGNCNGGACCGGCCTCATGCATACATCTGAACCTGACTCCAGAAACCTGCCG 180

** *** ** * ***** * * * * *** * * * **

REV_AGM_CON_SEQ_1 ---G----------------------------GTGGGAATTGGTGAGAACAAATGAGATT 99

MV_589246-1073_71_HMBSrevG01.ab1 TCAGGTAAGACCCCCACTTCGACGCACAGCTAATNNCCCTGGATCNCTAGGATCTCNAAT 240

* * * * * * * * *

REV_AGM_CON_SEQ_1 ATATGCACTC---NTGTTTATTAC---------CCCCTCACCCTCCAGCTTTGGTACCT- 146

MV_589246-1073_71_HMBSrevG01.ab1 GCTTGGGATCTTCCTNTTTNNTNTGCGAGGATNCCCNTGCTCCTCGCCCCTTGGCTAANG 300

** ** * *** * *** * **** * ****

REV_AGM_CON_SEQ_1 ------------------------------------------------------------ 146

MV_589246-1073_71_HMBSrevG01.ab1 CATGCGGTGCCAGATCNGATGCTTCCTCTTGGCCCTAACTCAGCTCTCCTTTTCAGTGAC 360

REV_AGM_CON_SEQ_1 -----------------------------------------------------GGGCA-- 151

MV_589246-1073_71_HMBSrevG01.ab1 TTTCACTACNNGAGTCTGTGATAAGCCCAAACTCTTCCTTCCCTGTCGCTCTTGAGGAGC 420

* * *

REV_AGM_CON_SEQ_1 --GGGACATGGAT---GGTAGCCTGCATGGTGTCTTGTATGCTATCTGAGCCATCTAGA- 205

MV_589246-1073_71_HMBSrevG01.ab1 CATCGACNTGGATCCGGGAAGCNTGCNGNGTGTCCTTCCTCGGATAGNTGCTCGCAAGCG 480

*** ***** ** *** *** ***** * * ** ** * **

REV_AGM_CON_SEQ_1 --CTCCAGACTCCTCCAGT-------------- 222

MV_589246-1073_71_HMBSrevG01.ab1 GAANAAAAAATGCTCCTNGGCGGNNNNNNACTC 513

* * * ****

Mosquito 72

REV_AGM_CON_SEQ_1 -------------GTTACGAGCAGTGA-------------TGCCTACCAGCTGTGGGTCA 34

MV_589246-1074_72_HMBSrevG02.ab1 NNNNNNNNNNNNNNNCNNGCGCANTTNNNNNTAANGACCATGCATCAGCAGTGTGGCGTA 60

* *** * *** * ***** *

REV_AGM_CON_SEQ_1 TCCTC---------------------------------------------AGGGCCATCT 49

MV_589246-1074_72_HMBSrevG02.ab1 ACTTCGATACACTTTAGCGATGTGATAATGGCAGCTGTTGATTACTTTTCTCCAGCAGAC 120

* ** **

REV_AGM_CON_SEQ_1 TCATGCTGTATGCGGGAA------------------------------------------ 67

MV_589246-1074_72_HMBSrevG02.ab1 TCTTCTAGTACTCTGGANTGGTGTGACTCATCTCTCAGTACCAGACTCCAGACTCCTCCA 180

** * *** * ***

REV_AGM_CON_SEQ_1 -------------------------------------GGAGGTGGGAATTGGTGAGAACA 90

MV_589246-1074_72_HMBSrevG02.ab1 GTCAGGTAAAGTCCTCCAGTCATGTTATTCCGNTCGNNTAATTCGGGTATGGTGTCGAAC 240

* * ** ***** *

REV_AGM_CON_SEQ_1 A-----------ATGAGATTATATGCACTCNTGTTTATTACCCC-CTCACCCTCCAGCTT 138

MV_589246-1074_72_HMBSrevG02.ab1 AGTCACGCAACGCTGNTGTTGTGGCNCNGATTGGNNGGTNNCGAGGCNACTGANCCGTNT 300

* ** ** * ** * * ** * * *

REV_AGM_CON_SEQ_1 TGGTACCTGGGCAGGGACATGGATGGTAGCCTGCATGGTGTCTTGTATGCTATCTGAGCC 198

MV_589246-1074_72_HMBSrevG02.ab1 AGGTGGTTTGGAGGGG-C-----------NTTGGGTGTGGTTGAGACACCGTANAGACTC 348

*** * ** *** * ** ** ** * * ** *

REV_AGM_CON_SEQ_1 ATCTAGACTCCAGACTCCTCCAGT------------------------------------ 222

MV_589246-1074_72_HMBSrevG02.ab1 CANCANACTCCAGACTCCACCAGTCAAGNNANCTCANNACTCCTCGAACCCGGTAACGGN 408

* ************ *****

REV_AGM_CON_SEQ_1 ------------------------------------------------------------ 222

MV_589246-1074_72_HMBSrevG02.ab1 GGNGACGCNNCCGGGCATCCTGGTCCAAATGNAGANCATCNNGTCCAGANANCGGNNAGA 468

REV_AGM_CON_SEQ_1 ---------------------- 222

MV_589246-1074_72_HMBSrevG02.ab1 ACCTGGGCATCGNNNGGCAANN 490

Mosquito 73

REV_AGM_CON_SEQ_1 ------------------------------------------------------------ 0

MV_589246-1075_73_HMBSrevG03.ab1 NNNNNNNNNNNNNNNNNNNNNCGGAAATTANNNACTCACNCTCGTCGGTTCCGCAAANNA 60

REV_AGM_CON_SEQ_1 -------------------------------GTTACGAGCAGTGATGCCTACCAGCTGTG 29

MV_589246-1075_73_HMBSrevG03.ab1 TTCAGNAAGCTGNAGCTNNTGGCTGATCCATCTGTCAACGACTCATGACTACAACGGGTN 120

* * * * * *** **** * **

REV_AGM_CON_SEQ_1 G--GTCATCCT------------------------------------------------- 38

MV_589246-1075_73_HMBSrevG03.ab1 GGGGTCANAATTCCNNCTCCTCCGNAANNGTAAATGGTAAGTTGACTTTTGTATTCTTGT 180

* **** *

REV_AGM_CON_SEQ_1 --------------------------------CAGGGCCATCTTCATGCTG--------- 57

MV_589246-1075_73_HMBSrevG03.ab1 AACTGGGACANNGATNACAGGTTNTCTTCCAGTAAGGAAGTGTTANTACTNTGGNGAGGG 240

* ** * ** * **

REV_AGM_CON_SEQ_1 ------------------------------------------------------------ 57

MV_589246-1075_73_HMBSrevG03.ab1 GANGAGCTNNANNANCTTTTGNCATGGAAGGCTAGTTCTTAGGANCNCCNGCTNTGAGCN 300

REV_AGM_CON_SEQ_1 ----------------------------TAT-----GCGGGAAGGAGGTGGGAATTGGTG 84

MV_589246-1075_73_HMBSrevG03.ab1 NTGNCTTGTCTCNCAGCNNTTAATTCTGTTTTGCCAGCCCTCCAGTACTATCAATTCAGG 360

* * ** * * **** *

REV_AGM_CON_SEQ_1 AGAACAA--ATGAGATTATATGCACT--------CNTGTTTATTACCCCCTCA--CCCTC 132

MV_589246-1075_73_HMBSrevG03.ab1 AGANAANGTCTGTGAANAAAATCAGTCCTTNNNNATTGTTGAGTNAGGCCTGNTGNACAC 420

*** * ** ** * * ** * **** * * *** * *

REV_AGM_CON_SEQ_1 CAGCTTTGGTACCTGGGCAGGGACATGGATGG--TAGCCTGCATGGTGTCTTGTATGCTA 190

MV_589246-1075_73_HMBSrevG03.ab1 GGGATGTGGGAAACGGGGCGCTGCNNTCCTGCGGATATGCTCGTGGCGAACTTCCACCAA 480

* * *** * *** * * ** * *** * * * *

REV_AGM_CON_SEQ_1 TCTGAGCCATCTAGACTCCAGACTCCTCCAGT--------- 222

MV_589246-1075_73_HMBSrevG03.ab1 AANGTTCCTTGGAGACTCAANACTCCTCCAGTCATGTAANN 521

* ** * ****** * ***********

Mosquito 75

REV_AGM_CON_SEQ_1 ------------------------------------------------------------ 0

MV_589246-1077_75_HMBSrevG05.ab1 NNNNNNNNNNNNCNNNNNNNGNNNACTCCNNACTCCTCCACTCNGGTNNTACGTAGACTC 60

REV_AGM_CON_SEQ_1 ------------------------------------------------------------ 0

MV_589246-1077_75_HMBSrevG05.ab1 NTNAGTCAGGTAANNCTCCNGGCTCCNACACCCGTCAAANCTCAANNGTNTACTGGTGNN 120

REV_AGM_CON_SEQ_1 -GTTACGAGCAGTGATGCCTACCAGCTGTGGGTCATCCTCAGGGCCATCTTCATGCTGTA 59

MV_589246-1077_75_HMBSrevG05.ab1 TGTCANNCTTCGGGCTGCTTCATAACTGAGNGTCCGACTCCGACTCAT------NCGGTA 174

** * * * *** * * *** * *** *** * *** * ***

REV_AGM_CON_SEQ_1 TGCGGGAAGGAGGTGGGAATTGGTGAGAACAAATGAGATTATATGCACTCN--------- 110

MV_589246-1077_75_HMBSrevG05.ab1 GNG-----NGANGANCCCCACTCNGANNNNNCATGAACCTCCNTGNATCCNTGTGNTACT 229

** * ** **** * ** * **

REV_AGM_CON_SEQ_1 ---------------------------------------------------------TGT 113

MV_589246-1077_75_HMBSrevG05.ab1 NAAAACCTTTGCTGTTCCNNATTTCAGTGGGAATGTTTTCCNGGCGAANNGGGTNNTGGN 289

*

REV_AGM_CON_SEQ_1 TTATTACCCCCTCACCCTCCAGCTTTGGTACCTGGGCAGGGACATGG------------- 160

MV_589246-1077_75_HMBSrevG05.ab1 NAATTTCTGCGTCGCCATCNAAGGTCTCTNCCGGTGAGGGAAAACTGAGCCCTTCTTTTG 349

*** * * ** ** ** * * * ** * * ** * * *

REV_AGM_CON_SEQ_1 ------------------------------------------------------------ 160

MV_589246-1077_75_HMBSrevG05.ab1 AGAGACTTNNCTGCGAGGGTGAGGCAGAGGCCCAAACTCTGCCTACCCCCTCTCTGTTGG 409

REV_AGM_CON_SEQ_1 --------------------ATGGTAGCCTGCATGGTGTCTTGTATGCTATCT--GAGCC 198

MV_589246-1077_75_HMBSrevG05.ab1 GGAGGGATCCCNGNTCTCTGCTCCTCGTGCGNGNGGAATTTGCACGGACATCTGCCTTCC 469

* * * * ** * * * **** **

REV_AGM_CON_SEQ_1 ATCTAGACTCCAGACTCCTCCAGT---------- 222

MV_589246-1077_75_HMBSrevG05.ab1 GGGGNNNNNCCNGACTCCTCCNNTCAAGAAATNN 503

** ********* *

Mosquito 76

REV_AGM_CON_SEQ_1 ------------------------------------------------------------ 0

MV_589246-1078_76_HMBSrevG06.ab1 NNNNNNNNNNTTGTGTCGGCACACTGGNNTTTCCCGACTTGGAGAGACGTAGTTCCCTCC 60

REV_AGM_CON_SEQ_1 -----------------------GTTACGAGCAGTGATGCCTACCAGCTGTGG---GTCA 34

MV_589246-1078_76_HMBSrevG06.ab1 CAGACCAAAGCCTATGAGGGCAGCTTATCGGTGGTGTGACAGATCAGCCGTCGGGGCTCA 120

*** * *** * * **** ** * ***

REV_AGM_CON_SEQ_1 TCCTCAGGGCCATCTTCATGCTG----------------------------TATGC---- 62

MV_589246-1078_76_HMBSrevG06.ab1 AGCCCTGAGCAAACGTGGTGCGGGACTGCTGGGTGTCTGTCACCTCTGCGCTGTCCTAGA 180

* * * ** * * * *** * * * *

REV_AGM_CON_SEQ_1 -GGGAAGGAGGTGGGAATTGGTGAGA---------------------------------- 87

MV_589246-1078_76_HMBSrevG06.ab1 AGGCAAGGGGGCTTAAACTGCTCCATCCAAAGACTCCAGACTCCTCCAGTCAGGTAAAGG 240

** **** ** ** ** *

REV_AGM_CON_SEQ_1 --ACAAATGAGATTATATGCACTCNTGTTTATTACCCCCTCACCCTCC------------ 133

MV_589246-1078_76_HMBSrevG06.ab1 CCAGGAATGAGAGTTTTGTCCTACTTTGGGATAAGCTGGGACTGCTCCATTCTGGTAATG 300

* ******* * * * * * ** * * ****

REV_AGM_CON_SEQ_1 -----------------AGCTTTGGTACCTGGGCAGGGACATGGATGGTAGCCT------ 170

MV_589246-1078_76_HMBSrevG06.ab1 GAGAGGGTGTAANANACTCCAATTGCCCCTGGACCAGGTAAACANAAGTGCGCTGGCTTT 360

* * * ***** * ** * ** **

REV_AGM_CON_SEQ_1 --------GCATGGT--GTCTTGTATGCTATCTGAGCCATCTAGACTCCAGACTCC---- 216

MV_589246-1078_76_HMBSrevG06.ab1 ATTATTCTGCCTCTNGTGAACTATATCAATTCTTGCATANGAAGACTGGGGGCCCTCCCC 420

** * * * *** *** * ***** * * *

REV_AGM_CON_SEQ_1 ------------------------------------------------------------ 216

MV_589246-1078_76_HMBSrevG06.ab1 ACACAGCCTCTGCTCAGATAANTTTTTTCACCTGTGATGCNNCCATGAAAACATTCGNAT 480

REV_AGM_CON_SEQ_1 --TCCAGT---------------------------------------------------- 222

MV_589246-1078_76_HMBSrevG06.ab1 NCCGCAGGTGTGCGACATCTTTTCTCCTACCCCCCCTTCACAGCTCACAATTTTTTGGGA 540

***

REV_AGM_CON_SEQ_1 ------------------------------------------------------------ 222

MV_589246-1078_76_HMBSrevG06.ab1 CACATCGCGGAGGGGGAGGAGAGAGGAAAACCTGCCTGGGCCTCTGACANANACTNNGTT 600

REV_AGM_CON_SEQ_1 - 222

MV_589246-1078_76_HMBSrevG06.ab1 N 601

Mosquito 78

REV_AGM_CON_SEQ_1 --GTTACGAGCAGTG----ATGCC-------TACCAGCTG----TGGGTCATCCTCAGGG 43

MV_589246-1080_78_HMBSrevG08.ab1 NNNNNNNNNNNNNTGACCCATCGCNACNACNNAACTGCTGGACCGTGGCCAGACTCCAGA 60

** ** * * * **** ** ** *** *

REV_AGM_CON_SEQ_1 CCATCTTCATGCTGTATGCGGGA----AGGAGGTGGGAATTGGTGAGAACAA-------- 91

MV_589246-1080_78_HMBSrevG08.ab1 CTCCTCCAGTCAGGTAAATGTCATAATGGCAGCTGTTGATTACTTTTCTCCANNANACTC 120

* * *** * * * ** ** *** * * *

REV_AGM_CON_SEQ_1 ATGAGATTATATGCACTCNTGT---------TTATTACC------CCCTCACCCTCCAGC 136

MV_589246-1080_78_HMBSrevG08.ab1 TTCTANTACTCTGGANNGNTGTGACTCATCTCTCAGTACCAGACTCCAGACTCCTCCAGT 180

* * * ** * **** * * ** *******

REV_AGM_CON_SEQ_1 TTTGGTACCTGGGCAGGGACATGGATGGTAGC----CTGCATGGTGTCTTGTATGCTATC 192

MV_589246-1080_78_HMBSrevG08.ab1 C-AGGTAAT-GGGGGGGGGGACCGGTGGTGGTGATTTTCCTCCGNGTNGGCTGTACTCAN 238

**** *** *** * * **** * * * * ** * * **

REV_AGM_CON_SEQ_1 TGAGCCATCT---AGACTCCAGACTCCTCCAGT--------------------------- 222

MV_589246-1080_78_HMBSrevG08.ab1 GTACCGCTGGGACGCNGTGGCGACTNCTGGGGTTGTGAAAGACTGCANANGTNTCNANGG 298

* * * * **** ** **

REV_AGM_CON_SEQ_1 ----------------------- 222

MV_589246-1080_78_HMBSrevG08.ab1 TGGTAATTATTTGTGAACAGGGG 321

Mosquito 81

REV_AGM_CON_SEQ_1 -------------GTTACGAGCAGTGATGCCTACCAGCTGTGGGTCATCCTCAGGGCCAT 47

MV_589246-1083_81_HMBSrevG11.ab1 NNNNNNNNNNNNNNTNNCNTNNNNTGCNNACTCCAGACT----NCTCCNCTCAGGTAANA 56

* * ** ** * ** ******

REV_AGM_CON_SEQ_1 CTTCATGCTGTATGCGGGAAGGAGGTGGGAATTGGTGAGAACAAATGAGATTATATGCAC 107

MV_589246-1083_81_HMBSrevG11.ab1 CNCCANACTCGTT------------------CTGTCGTGTAAATGTGACAATGGTTGCTG 98

* ** ** * ** * * * * *** * * ***

REV_AGM_CON_SEQ_1 TCNTGTTTATTACC--CCCTCACCCTCCAGCTTTGGTA-CCTGGGCAGGGACATGGATGG 164

MV_589246-1083_81_HMBSrevG11.ab1 TTGATTTCTTTTCTCGANATTACTCTACTCCTATTCTGGATTGGGGTGACTCATATCTCA 158

* ** ** * * ** ** * ** * * **** * *** *

REV_AGM_CON_SEQ_1 TAGCCTGCATGGTGTCTTGTATGCTATCTGAGCCATCTAGACTCCAGACTCCTCCAGT-- 222

MV_589246-1083_81_HMBSrevG11.ab1 ATTCCTG--------ACTCCCGACTCCTCCAGTCAGGTAAACTCCAGACTCCTCCAGTCA 210

**** * ** ** ** ** ******************

REV_AGM_CON_SEQ_1 ---- 222

MV_589246-1083_81_HMBSrevG11.ab1 GGTA 214

Mosquito 82

REV_AGM_CON_SEQ_1 ----------------------------GT---------TACGAGCAGTGATGCCTACCA 23

MV_589246-1084_82_HMBSrevG12.ab1 NNNNNNNNNNNNTTNTCNGCGCANNTNNATTAACACCANCATCCGCAGTGTGACGTANCT 60

* * ****** * ** *

REV_AGM_CON_SEQ_1 ------------------------------GCTG-TGGGTCATC---------------- 36

MV_589246-1084_82_HMBSrevG12.ab1 TCGATNCACTTTACCAATGTGATAATGGCACCTGTTGATTACTTTTCTCCAACATACTCT 120

*** ** * *

REV_AGM_CON_SEQ_1 ------------------------------------------------------------ 36

MV_589246-1084_82_HMBSrevG12.ab1 TCTACTACTCTGAATTGGTGTGACTCATCTCTCAATACCTGACTCCAAACTCCTCCAGTC 180

REV_AGM_CON_SEQ_1 --CTCAGGGCCATCTTCATGCTGTATGCGGGAAGGAGGTGGGAATTGGTGAGAACAAATG 94

MV_589246-1084_82_HMBSrevG12.ab1 TCGTAAACTCCAGACTCCTCCAGTCAGGTAAGTACNAGATGAGTTAGGTGGNCAAAAGTN 240

* * *** ** * * ** * * * * **** * ** *

REV_AGM_CON_SEQ_1 AGA-----------------------------------------------TTATATGCAC 107

MV_589246-1084_82_HMBSrevG12.ab1 TGGGTCTGCTGNNATTCCAAATTCCTCCGGGCGGGTAAGAGNNNCGTGTACTCGAGACGG 300

* * * *

REV_AGM_CON_SEQ_1 TCNTGTTTATTACCCCCT------CACCCTCCAGCTTTGGTACCTGGGCAGGGACATGGA 161

MV_589246-1084_82_HMBSrevG12.ab1 TGGTTTTTTGTGGAACGGGGCGCCGAAANGGCAGCATCGATGGCGGGGGCNTGAGCTANA 360

* * *** * * * **** * * * * *** ** * *

REV_AGM_CON_SEQ_1 T--GGTAGCCTGCATGGTGTCTTGTATGCTATCTGAGCCATCTAG------ACTCCAGAC 213

MV_589246-1084_82_HMBSrevG12.ab1 GGGGCTAGGNTN-GGNATANCNTNGCTTTTACATGACCGNCATCGCCCTGCGCCCGACAC 419

* *** * * * * * ** *** * * * * * * **

REV_AGM_CON_SEQ_1 TCCTCCAGT------------------------------------------ 222

MV_589246-1084_82_HMBSrevG12.ab1 GCAACCCGACCGCNCTCGNTTGGGGGGGGGTNGGAGCCACCCCTTNTANCA 470

* ** *

Mosquito 83

REV_AGM_CON_SEQ_1 ------------------------------------------------------------ 0

MV_589246-1085_83_HMBSrevH01.ab1 NNNNNNNNNANNNNNNNNANNNNAGGAGATCNNCTTCNNTATTNNCCNGACGNGGAGTAT 60

REV_AGM_CON_SEQ_1 --------------------------------GTTACGAGCAGTGATGCCTACC------ 22

MV_589246-1085_83_HMBSrevH01.ab1 GGTGAATNNTGGNTCTCTAAATCACCCTGCCTGTCGTGTGAATCAATCCCTCACGAATTT 120

** * * * ** *** *

REV_AGM_CON_SEQ_1 -------AGC-------------------------------------------------- 25

MV_589246-1085_83_HMBSrevH01.ab1 GCNACTCATGTAACGTTACTCCTCCGTCCTGGTAACATAATCAACAGACTGATCAGGAAC 180

*

REV_AGM_CON_SEQ_1 -------------------------------------------------TGTGGGTCATC 36

MV_589246-1085_83_HMBSrevH01.ab1 GTACAGGAGACTCCAGACTCCTCCAGTCAGGTAANNGACTCGTCTGCCAGGAGAGTCATC 240

* * ******

REV_AGM_CON_SEQ_1 CTCA---------------------------------------GGGCCATCTTCATGCTG 57

MV_589246-1085_83_HMBSrevH01.ab1 ATCCCTTTACGGTCCACTATTTCCGAGGGAATGCTTTTCCCCCGACACGGCTCCCTGGNA 300

** * * ** * **

REV_AGM_CON_SEQ_1 TATGCGGG-------------------AAGGAGGTGGGAATTGGTG---AGAACAAATGA 95

MV_589246-1085_83_HMBSrevH01.ab1 AATACTGCTACCCCTTCAAACCTCTTCACTGGCGAGGGAAACAGTAACGTAAACTTTGGG 360

** * * * * * ***** ** *** *

REV_AGM_CON_SEQ_1 GATTATATGCACTCNTGTTTAT-------TACCCCCTCACCCTCCAGCTTTGGTACCTGG 148

MV_589246-1085_83_HMBSrevH01.ab1 AATCACATTCTGAGAGGATTATNCANATGTACACACTTCCACTGGCCTTTCAG-CGGCGG 419

** * ** * * **** *** * ** * ** ** * **

REV_AGM_CON_SEQ_1 GCAGGGACATGGATGGTAGCCTGCATGGTGTCTTGTATGCTATCTGAGCCATCTAGA--- 205

MV_589246-1085_83_HMBSrevH01.ab1 GTAGGGATCCCGGNATCNCTNCTCCTCNTTCCNCTGAAGTAANCACAGACATCTCCCTTC 479

* ***** * * * * * * * * * ** *****

REV_AGM_CON_SEQ_1 ------------------------------------------------------------ 205

MV_589246-1085_83_HMBSrevH01.ab1 CGGNNATNCTCNNNANGGCTCCAATCANGTAAGACCTCTTGCNGNGCTGCTTCTTCTGGT 539

REV_AGM_CON_SEQ_1 ------------------------------------------------------------ 205

MV_589246-1085_83_HMBSrevH01.ab1 GCGGANGATGATAANNTNNNTCGTNATTCACCACGNNGTGGTGTNGTANTTTTAACNGCT 599

REV_AGM_CON_SEQ_1 ------------------------------------------------------------ 205

MV_589246-1085_83_HMBSrevH01.ab1 CCACCGTGNGNNTCTCAATANNCAGCTATTCTTCTCAGTTGGGCTGGNTCTTATTNGGCA 659

REV_AGM_CON_SEQ_1 ---------CTCCAGACTCCTCCAGT------ 222

MV_589246-1085_83_HMBSrevH01.ab1 GCGTNAATACTCANNACTCCTCNNNCAAGNAA 691

*** *******

Mosquito 84

REV_AGM_CON_SEQ_1 ------------------------------------------------------------ 0

MV_589246-1086_84_HMBSrevH02.ab1 NNNNNNNNNNNNNNNNNNNNNNNNNNNTTTNNTCTCCTTNNAATGCCGATGACCGCNATC 60

REV_AGM_CON_SEQ_1 ------------------------------------------------------------ 0

MV_589246-1086_84_HMBSrevH02.ab1 ATATTTCTCAAAGCGGGGTAAGCACTGGCGCGGGATGCACCTTAATTGTGCAAACCACGC 120

REV_AGM_CON_SEQ_1 ------------------------------------------------------------ 0

MV_589246-1086_84_HMBSrevH02.ab1 TCTAGACTCCAGACTCCTCCAGTCAGGTAAANNCNCNCGCCGACANGCACCCCGTGCTAA 180

REV_AGM_CON_SEQ_1 ------GTTACGAGCAGTGATGCCTACCAGCT-------------GTGGGTCATCCTCAG 41

MV_589246-1086_84_HMBSrevH02.ab1 AGGGGGTTCCGATGCNTTGGAGGGTGCCAGCAGCGTCTCCGCCTTCTGGCTGTTCATCNT 240

* ** ** * * ***** *** * ** **

REV_AGM_CON_SEQ_1 GGCCATCTTCATGCTGTATGCGGGAA---------------------------GGAGGTG 74

MV_589246-1086_84_HMBSrevH02.ab1 GACTGCNTTCGTGTAAACNGCGANCACCTTGCTCGGCGANAATTCNNGAATCANCCAGTC 300

* * *** ** *** * **

REV_AGM_CON_SEQ_1 GGAATTGGTGAGAACAAATGAGATTATATGCACTCNTGTTTATTACCCCCTCACCCT--- 131

MV_589246-1086_84_HMBSrevH02.ab1 GGATATTGNGATCANNCGTTTGCNGGTATTNTCGGCAGATCCATGACCCCGACAGCTCCT 360

*** * * ** * * * *** * * * * **** **

REV_AGM_CON_SEQ_1 ----CCAGCTTTGGTACCTGGGCAGGGACATGGATGGTAGCCTGCATGGTGTCTTGTATG 187

MV_589246-1086_84_HMBSrevH02.ab1 CCCACCACGCTCAAGATGT-TCCCCGGAAATCCTCGCCTGACTGCGCCAACTTCCATATN 419

*** * * * * *** ** * * **** * ***

REV_AGM_CON_SEQ_1 CTATCTGAGCCATCTAGACTCCAGACTCCTCCAGT------------------------- 222

MV_589246-1086_84_HMBSrevH02.ab1 CGGAGTGGCCCCGGGAAAAACGACAGNCGGGCCGGNGAACAGGCANNGAGTCNCGTTGGC 479

* ** ** * * * * * * * *

REV_AGM_CON_SEQ_1 ---------------------------------- 222

MV_589246-1086_84_HMBSrevH02.ab1 GTGGGCAGGNNNCTGGNAGTGCTNTNAAACTNTG 51

Mosquito 85

REV_AGM_CON_SEQ_1 ------------------------------------------------------------ 0

MV_589246-1087_85_HMBSrevH03.ab1 NNNNNNNNNNNNNNNNANNNGCANGNTACNANCNNNTCTACANTCNNAACATCTNAGCAT 60

REV_AGM_CON_SEQ_1 ------------------------------------------------------------ 0

MV_589246-1087_85_HMBSrevH03.ab1 GTNNCTGACGAGACNGCGGACCCTGGNNACTTTNATNATGCATCTAAATTCTACTTACCC 120

REV_AGM_CON_SEQ_1 ------------------------------------------------------------ 0

MV_589246-1087_85_HMBSrevH03.ab1 CCCTCACNCAACCAGATTGGTAATGAAANACCGGATTGACTGGTTGCNAAGCTGGTCGCA 180

REV_AGM_CON_SEQ_1 ---------------------------------------GTTACGAGCAGTGATGCCTAC 21

MV_589246-1087_85_HMBSrevH03.ab1 TTTGCTGNNCTTATACTTCTCCTCCAGGACTGGAGAGGNNGGANGCCCNGNTNTTTCCAN 240

* * * * * * *

REV_AGM_CON_SEQ_1 CAGCTGTGGGTCATCCTCAGGGCCATCTTCAT---------------------------- 53

MV_589246-1087_85_HMBSrevH03.ab1 CGACTGAGGACATACTTCAATGCTATCCTACNNCAACGGCACAGAGCGCATGNTTATACC 300

* *** ** * *** ** *** *

REV_AGM_CON_SEQ_1 ------------------------------------------GCTGTATGCGGGAAGGAG 71

MV_589246-1087_85_HMBSrevH03.ab1 GCGGATGTGAAAATCCTTNCGACTCCCNACACCTCCATTAACGTAATCTACCGGACTCTT 360

* * * * ***

REV_AGM_CON_SEQ_1 GTGGGAATTGGTGAGAACAAATGAGAT--------------------------------- 98

MV_589246-1087_85_HMBSrevH03.ab1 CTCGGTGGCGCTCCGTCCANATATGCGCCATCCCAGTCGACTTTCTGCCGCGACCACTTC 420

* ** * * * ** ** *

REV_AGM_CON_SEQ_1 ------TATATGCACTCNTGTTTATTACCCCCTCACCCTC---CAGCT-----------T 138

MV_589246-1087_85_HMBSrevH03.ab1 CCCTGTGCTACTCTCTCATGTTTTGAACNTGCTGACCACCTTACTGGTNTCTACTCCGAT 480

** * *** ***** ** ** *** * * * * *

REV_AGM_CON_SEQ_1 TGGTACCTGGGCAGGGACATGG-------------------------------------- 160

MV_589246-1087_85_HMBSrevH03.ab1 TATTAATTGGGGGGTTACATCGNNNGANCTCCTCCNGCGCAATTTCTTCNNGTGCGGATG 540

* ** **** * **** *

REV_AGM_CON_SEQ_1 ------------------------------------------------------------ 160

MV_589246-1087_85_HMBSrevH03.ab1 AAGATCANNNANNTCNNNATATCACCACNNNTTGCTGGGGTAGNTTTNANNGCTCCACCG 600

REV_AGM_CON_SEQ_1 ----------------------ATGGTAGCCTGCATGGTGTCTTGTATGCTATCTGAGCC 198

MV_589246-1087_85_HMBSrevH03.ab1 GACTGACTCTCANCAGANNGCTANTGTTCTCGTTACGGNCTGCNNTCTTANANNNNAANN 660

* ** * * ** * * * * *

REV_AGM_CON_SEQ_1 ATCTAGACTCCAGACTCCTCCAGT------- 222

MV_589246-1087_85_HMBSrevH03.ab1 GTTGAGACTCCAGACTCCTCCAGTCAGGTAA 691

* ********************

Mosquito 86

REV_AGM_CON_SEQ_1 ------------------------------------------------------------ 0

MV_589246-1088_86_HMBSrevH04.ab1 NNNNNNNNNNNNNTNGNNNNNNAGNTACNNTCNNGACNNGTTTNNNAGAGTNTAAGCTAA 60

REV_AGM_CON_SEQ_1 ------------------GTTACGAGCAGT-------GATGCCTACCAGCTGTGGGTCAT 35

MV_589246-1088_86_HMBSrevH04.ab1 NATTCCCTNTAGATAGACGTTCCTGGACANCTTCTTTTAAGGNTCTCCCTTCTANATACC 120

*** * * * * * * * * *

REV_AGM_CON_SEQ_1 CCTCAGGGCCATCTTCATGCTGTATGCGGGAAGGA------------------------- 70

MV_589246-1088_86_HMBSrevH04.ab1 CCCNTCGGACTTCTGTTTGGTGAGTGAGGACCGGCGCGACTGGTGGCCAGTCCGGTCTCA 180

** ** * *** ** ** ** ** **

REV_AGM_CON_SEQ_1 -----------------------------GGTGGGAATTGGTGAGAACAAATGAGATT-- 99

MV_589246-1088_86_HMBSrevH04.ab1 TTTNNGCCACTTATCCTCCGGGTACTGGACTGGAGAATCTTCANNGCCCGNTTATCTTCC 240

* **** * * * **

REV_AGM_CON_SEQ_1 -----ATATGCACTCNTGTTTATTACCCCCTCACCCTCCAGCTTTGGTACCTGGGCAGGG 154

MV_589246-1088_86_HMBSrevH04.ab1 TCCGACTGAGGACATATTTTAATGCNATCCTANNCCACCGCCATAGATCGCATGATTATA 300

* * ** * ** ** *** ** ** * * * * * *

REV_AGM_CON_SEQ_1 ACATGGATGGTAGCCTGCA----------------------------------------- 173

MV_589246-1088_86_HMBSrevH04.ab1 TCACGGATGACCGACTCCTTGCGATGTTCTACTCCTCCATTAACGTAATCGACCGGACTC 360

** ***** * ** *

REV_AGM_CON_SEQ_1 ------------------------------------------------------------ 173

MV_589246-1088_86_HMBSrevH04.ab1 TTCTCGGTGGCGCTCCGTCCGTAAATGCACCATCCCAGCCGACTTTTTGCAGCGACCACT 420

REV_AGM_CON_SEQ_1 ------------------------------------------------------------ 173

MV_589246-1088_86_HMBSrevH04.ab1 TCCCCTGTGCTACTCTCTCGGCTTTTGAGCGTGCTGATCAGCTTAGCGTTGTCTACTCCG 480

REV_AGM_CON_SEQ_1 ------------------------------------------------------------ 173

MV_589246-1088_86_HMBSrevH04.ab1 ATTATTAATTGGGGGGTTACATCGGNNTNNCTCCTCAGGNGCAATTTCTTCANGTGCGGA 540

REV_AGM_CON_SEQ_1 ------------------------------------------------------------ 173

MV_589246-1088_86_HMBSrevH04.ab1 TGAAGATCACAAAGCTCGTCATANACCACGGTTTGCTTGNNTAGTTTTAACTGCTCTACC 600

REV_AGM_CON_SEQ_1 ------------------------------------TGGTGTCTTGTATGCTATCTGAGC 197

MV_589246-1088_86_HMBSrevH04.ab1 GTCCTGGCTCTCAACGGAAAGCTCCTGTTCTCGTTACGGCCTGCAATCTTANANNNGAAG 660

** * * * * **

REV_AGM_CON_SEQ_1 CATCTAGACTCCAGACTCCTCCAGT------- 222

MV_589246-1088_86_HMBSrevH04.ab1 CGTTGAGACTCCAGACTCCTCCNGTCAGGTAA 692

* * ***************** **

Mosquito 87

REV_AGM_CON_SEQ_1 ------------GTTACGAGCAGTGATGCCTACCAGCTGTGGGTCATCCTCAGGGCCATC 48

MV_589246-1089_87_HMBSrevH05.ab1 NNNNNNNNNNNNTTNNNGCGCANCTTTCCNTTAAGACCATGCATCAGCA---GTGNGGCG 57

* * *** * * * * ** *** * * *

REV_AGM_CON_SEQ_1 TTCATGCTGTATGCGGGAAGGAGGTGGGAAT-------------TGGTGAG---AAC--- 89

MV_589246-1089_87_HMBSrevH05.ab1 TAACTTCGATACACTTTAGCGATGTGATAATGGCAGCTGTTGATTACTTTTCTCCAGCAG 117

* * * ** * * ** *** *** * * *

REV_AGM_CON_SEQ_1 --AAATGAGATTATATGCACTCNTGTTTATTACCC-----CCTCACCCTCCAGCTTTGGT 142

MV_589246-1089_87_HMBSrevH05.ab1 ACTCTTCTAGTACTCTGGAGTGGTGTGACTCATCTCTCAGTACCAGACTCCAGACTC--- 174

* * * ** * * *** * * * ** ****** *

REV_AGM_CON_SEQ_1 ACCTGGGCAGGGACATGGATGGTAGCCTGCATGGTGTCTTGTATGCT------------- 189

MV_589246-1089_87_HMBSrevH05.ab1 CTCCAGTCAGGTAANGGCGTGCTGGTGTCGATGCTGNCGGGCANACTCCNGACTCNTCGG 234

* * **** * * ** * * * *** ** * * * **

REV_AGM_CON_SEQ_1 ------------------------------------------------------------ 189

MV_589246-1089_87_HMBSrevH05.ab1 TCANGNNAGGGGCCGTCGCANANGTCGGGCTGGNNNGAGGNGGGNGGGNGCGTCANGGTG 294

REV_AGM_CON_SEQ_1 -----------------------ATCTGAGCCATCTAGACTCCAGACTCCTCCAGT---- 222

MV_589246-1089_87_HMBSrevH05.ab1 GNGGNGGTTCTNNNGANACNGGTCGGCGCAGGTANNAGACTCTGGACTCCTCGGGCTAGN 354

* ****** ******** *

REV_AGM_CON_SEQ_1 ------------------------------------------------------------ 222

MV_589246-1089_87_HMBSrevH05.ab1 TGGGGCGCTCCNNGACAGCCAGNGNTTGCAACGCGTNANACGNNCACTGANGACGGCCCN 414

REV_AGM_CON_SEQ_1 ------------------------------------------------------------ 222

MV_589246-1089_87_HMBSrevH05.ab1 CTTGCAGCCCTCGCAACGAGCGGCGGTTCGGTGATANTGTNTGGTNTCTANTNACCGTCC 474

REV_AGM_CON_SEQ_1 ------------------------------------------------------------ 222

MV_589246-1089_87_HMBSrevH05.ab1 TCNTGTCGGTGGTTGTTCTAATAGANTCTGCTGNGCCGTGTNACANGCGGNGGAAGTCTN 534

REV_AGM_CON_SEQ_1 ----------- 222

MV_589246-1089_87_HMBSrevH05.ab1 ANAGTANGCTN 545

Mosquito 88

REV_AGM_CON_SEQ_1 ------------------------GTTACGAGCAG-TGATGCCTACCAGCTGTGGGTCAT 35

MV_589246-1090_88_HMBSrevH06.ab1 NNNNNNNNNNNNTNNCATANNNNNNTNACTCNNNANNCNTCCNCTCCAGTAANGGCGTAA 60

* ** * * **** ** *

REV_AGM_CON_SEQ_1 CCTCAGGGCCATCTTCATGCTGTATGCGGGAAGGAGGTGGGAATTGGTGAGAACAAA-TG 94

MV_589246-1090_88_HMBSrevH06.ab1 CTTC-GATACACTTTAGCGATG--TGATAATGGCAGCTGTTGATTACTTTTCTCCAACAG 117

* ** * ** ** * ** ** * ** ** *** * * ** *

REV_AGM_CON_SEQ_1 -------AGATTATATGCACTCNTGTTTATTA---------CCC-----CCTCACCCTCC 133

MV_589246-1090_88_HMBSrevH06.ab1 ACTCTTCTAGTACTCTGGANTGGTGTGACTCATCTCTCAGTACCAGACTCCAGACTCCTC 177

* * ** * * *** * * ** ** ** * *

REV_AGM_CON_SEQ_1 AGCTTTGGTACCTGGGCAGGGACATGGATGGTAGCCTGCATGGTGTCTT-------GTAT 186

MV_589246-1090_88_HMBSrevH06.ab1 CAGTCAGGTAAAGNGGGANNCGCNCNGGGCCGAGNGTACCANCTGGGTCNGGNGCNGAAA 237

* **** ** * * * ** * * ** * * *

REV_AGM_CON_SEQ_1 GCT------------------------ATCTGAGCCATCTAGACTCCAGACTCCTCCAGT 222

MV_589246-1090_88_HMBSrevH06.ab1 AGTANTGCNCGCTGCAATTGTTGGTTCGCCTGAGNGGAANGGCGGAANGTGTCNTCGNGA 297

* ***** * * ** ** *

REV_AGM_CON_SEQ_1 ------------------------------------------------------ 222

MV_589246-1090_88_HMBSrevH06.ab1 TGGTGGGNGTCAGTGANTCGGGGNGAGGGNANGGCAACCTAAAGATGTNNTNNA 351

Mosquito 89

REV_AGM_CON_SEQ_1 ----------------------------------------GTTACGAGCAGTGA------ 14

MV_589246-1091_89_HMBSrevH07.ab1 NNNNNNNNNNNNNANNNNNNGNNNNNTACNNNTTNGAGATGTTTCNNNGANTGTCTCCTT 60

*** * * **

REV_AGM_CON_SEQ_1 --TGCCTACC-AGCTGTGGGTC--------ATCCTCAGGGCCATCTTCATGCTGTATGCG 63

MV_589246-1091_89_HMBSrevH07.ab1 GNTTCNCTCGAAATCGAGGTTCTTGGACAACTTCTTTTNNTCCCTTTCCTTCTANNTACC 120

* * * * * ** ** * ** * *** * ** * *

REV_AGM_CON_SEQ_1 GGAAGGAGGTGG-GAATTGGTGAGAACAAATGAGATTA----TATGCACTCNTGTTTA-- 116

MV_589246-1091_89_HMBSrevH07.ab1 GGNNTCGGATAGCTGTCCGGTTAGGAAAGATCGGAACGACTGGNNNTTCTCCTGTCTCAT 180

** * * * *** ** * * ** ** *** *** *

REV_AGM_CON_SEQ_1 ------------------------------------------------------------ 116

MV_589246-1091_89_HMBSrevH07.ab1 CCTGCCATCCACCCTCCGGGTACTGTCCTGGAGAATCTTCACGGCCCCTTCATCTTCCTC 240

REV_AGM_CON_SEQ_1 --------------------TTACCCCCTCACCCTCCAGCTTTGGTACCTGGGCAGGGAC 156

MV_589246-1091_89_HMBSrevH07.ab1 CNACTGAGGACATACTTTAATGCAATCCTCNNCCACCGCCATAGATTGCCTGAATAGATC 300

* **** ** ** * * * * * * * *

REV_AGM_CON_SEQ_1 ATGGATGGTAGCCTGCA------------------------------------------- 173

MV_589246-1091_89_HMBSrevH07.ab1 GTGGATGTCCGACTCCTCGCGTTGTTCTACATGAGCATTAACGTAATCGACCGGACTCTT 360

****** * ** *

REV_AGM_CON_SEQ_1 ------------------------------------------------------------ 173

MV_589246-1091_89_HMBSrevH07.ab1 CTCGGTGGCGCTCCGTCCATAAATGCACCATCCCAGCCGAGTTTTTGCAGCGACCACTTC 420

REV_AGM_CON_SEQ_1 ------------------------------------------------------------ 173

MV_589246-1091_89_HMBSrevH07.ab1 CCCTGTGCTACTCTCTCGGCTTTTGAGCGTGCTGATCAGCTTAGCGTTGTCTACTCCGAT 480

REV_AGM_CON_SEQ_1 ------------------------------------------------------------ 173

MV_589246-1091_89_HMBSrevH07.ab1 TATTAATTGGGGGGTTACATCGGTATANCTCCTCAGGGGCAATTTCTTCAGGTGCGGATG 540

REV_AGM_CON_SEQ_1 ------------------------------------------------------------ 173

MV_589246-1091_89_HMBSrevH07.ab1 AAGATCACAAAGCTCGTCATAAACCACGGTTTGCTGGGGTAGTTTTAACTGCTCTACGGT 600

REV_AGM_CON_SEQ_1 ----------------------------------TGGTGTCTTGTATGCTATCTGAGCCA 199

MV_589246-1091_89_HMBSrevH07.ab1 CCTGGCTCTCAACGGAAAGCTCCTGTTCTCGTTACGGCCTGCNNTNNTANNNNCGANNCG 660

** * * ** *

REV_AGM_CON_SEQ_1 TCTAGACTCCAGACTCCTCCAGT-------- 222

MV_589246-1091_89_HMBSrevH07.ab1 TTGAGACTCCAGACTCCTCCNNTCANGTAAA 691

* ***************** *

Mosquito 91

REV_AGM_CON_SEQ_1 ------------------------------------------------------------ 0

MV_589246-1093_91_HMBSrevH09.ab1 NNNNNNNNNNNGTGANNNCTNCNTANCTNNTGAACNTCNCCCAACCAACTGAAAGTTTCN 60

REV_AGM_CON_SEQ_1 ----------------GTTACGAGCAGTG---------ATGCCTACCAGCTGTGGG---- 31

MV_589246-1093_91_HMBSrevH09.ab1 NNAGTTTGAACGACTCGATGATAGCTCAACAGTCTAACCTGTCTGTGAGCTGTTTGATGG 120

* * *** ** ** ****** *

REV_AGM_CON_SEQ_1 ---------------TC---ATCCTCAGGGCCATCTTCATGCTGTATGCGGGAAGGAGGT 73

MV_589246-1093_91_HMBSrevH09.ab1 ACCAACAGCTTCTGCTAACTGACCATAGAAACACAGCCATCCAGCATGGTCCAGAGAGAC 180

* ** ** ** *** * * *** * ***

REV_AGM_CON_SEQ_1 GGGAATTGGTGAGAACAAATGAGATTATATGCACTCNTGTTTATTA-CCCCCTCACCCTC 132

MV_589246-1093_91_HMBSrevH09.ab1 TCCA----GACTCCTCCAGTCAGGTAAGTTCCCGCTCAACTTATGCTCCTCCACGACTTC 236

* * * * * ** * * * * **** ** ** * * **

REV_AGM_CON_SEQ_1 CAGCTTTGG--TAC-CTGGGCAGGGACATGGATGGTAGCCTGCATG-------------- 175

MV_589246-1093_91_HMBSrevH09.ab1 CCACTCCAAACTCCTCCATCAAGGGAACAGCGCCGTTTGCGTCTTGTTGCCGTANTACAG 296

* ** * * * ***** * ** * * **

REV_AGM_CON_SEQ_1 ---------GTGTCTTGTATGCTATCTGAGCCATCTAGACTCCAGACTCCTCCAGT---- 222

MV_589246-1093_91_HMBSrevH09.ab1 ACTCCNNACTCCTCCTCTCAAGTAATCCAGTCAGGTAAACTCCAGACTCCTCCAGACTCG 356

** * * ** ** ** ** *****************

REV_AGM_CON_SEQ_1 ------------------------------------------------------------ 222

MV_589246-1093_91_HMBSrevH09.ab1 TAAGAACAGGAAANGTGNGGGATGCATCCCCGGCTNGTTTGAGATGGGGCGACANGGATG 416

REV_AGM_CON_SEQ_1 ------------------------------------------------------------ 222

MV_589246-1093_91_HMBSrevH09.ab1 ANGCACNACACCANCNACGTNACCCGGTGGAGGAAGGGGNCNTGAAACTCCNTCCCATCG 476

REV_AGM_CON_SEQ_1 ------------------------------------------------------------ 222

MV_589246-1093_91_HMBSrevH09.ab1 GTGTCCCAATGCCCTNGGAGCACCACAGNACCNGGGGTCTATATATNCACGATAATCCGT 536

REV_AGM_CON_SEQ_1 ----------------- 222

MV_589246-1093_91_HMBSrevH09.ab1 GAAAAANCCGAGNCTAN 553

Mosquito 92

REV_AGM_CON_SEQ_1 ------------------------------------------------------------ 0

MV_589246-1094_92_HMBSrevH10.ab1 NNNNNNNGNNNANTGTGTCGNCACACNNNNAATTTCCCGACTTGGAGAGCTGTAGTTCCC 60

REV_AGM_CON_SEQ_1 --------------------------GTTACGAGCAGTGATGCCTACCAGCTGTG---GG 31

MV_589246-1094_92_HMBSrevH10.ab1 TCCCAGACCAAAGCCTATGAGGGCAGCTTATCGGCGGCGTGACAGATCAGCCGTCGGGGC 120

*** ** * * * * **** ** *

REV_AGM_CON_SEQ_1 TCATCCTCAGGGCCATCTTCATGC--------TGTATGCGGGAAGGAGGTGGGAATTGGT 83

MV_589246-1094_92_HMBSrevH10.ab1 TCAAGCCCTGAGCAAACGTGGTACGGGACTGCTGGGTGTCTGTCACCTCTGCGCTGTCCT 180

*** * * * ** * * * * * ** ** * ** * * *

REV_AGM_CON_SEQ_1 GAGAACAAATGAGATTATATGCACTCNTGTTTATTACCCCCTCACCCTCCAGC------- 136

MV_589246-1094_92_HMBSrevH10.ab1 AGAAGGCAAGGGGGCTTAAACTGCTCCATCCAAAGACTCCAGACTCCTCCAGTCAGGTAA 240

* ** * * * * *** * ** ** *******

REV_AGM_CON_SEQ_1 -------TTTGGTACCTGGGCAGGGACATGGATGGTAGCCTGCATGGTGTCTTGTATGCT 189

MV_589246-1094_92_HMBSrevH10.ab1 GGGGCNGGTTGGGACTTTGGCTTCCTTGTGGATCAGCGCNTGCCATTTCGCTCNTGGGAT 300

**** ** * *** ***** ** *** * ** * * *

REV_AGM_CON_SEQ_1 AT--CTGAGCCATCTAGACTCCAGACTCCTCCAGT------------------------- 222

MV_589246-1094_92_HMBSrevH10.ab1 GGAGACCACTGTAACANACTCCAAACTCCCCCGGACCGGTAAACAGAAGTGCTACCNGAT 360

* * ****** ***** ** *

REV_AGM_CON_SEQ_1 ------------------------------------------------------------ 222

MV_589246-1094_92_HMBSrevH10.ab1 TATTTTTCTGCCTCTGGTTAAATAGATGAATTCTTGCATAAATAAACCGGGGGGATCCTA 420

REV_AGM_CON_SEQ_1 ------------------------------------------------------------ 222

MV_589246-1094_92_HMBSrevH10.ab1 CACANATGGGCTGCTCAAAGATTNCCTCTNACCNCGNNGCCTCCCNNTCAACTTTCCGTT 480

REV_AGM_CON_SEQ_1 ------------------------------------------------------------ 222

MV_589246-1094_92_HMBSrevH10.ab1 GCTTCCGCTGTGGNGCATGTNTTTCTTGNCTGCCNGTCNCAGNTATNAANNNNTCGGNNT 540

REV_AGM_CON_SEQ_1 -------------------------------------------------- 222

MV_589246-1094_92_HMBSrevH10.ab1 GCACTGNGNGAAGAANNNGNNANANNANNNNNNNGNNNATCTNNNNNNCA 590

Mosquito 93

REV_AGM_CON_SEQ_1 ------------------------------------------------------------ 0

MV_589246-1095_93_HMBSrevH11.ab1 NNNNNNNNNNNNNNNNNNNNNTNNNNNNNCCCCCACTNNCCTGNTNCCGTGTGTGAGAGC 60

REV_AGM_CON_SEQ_1 ------------------------------------------------GTTACGAGCAGT 12

MV_589246-1095_93_HMBSrevH11.ab1 CCCCGAAAGGCTGGACACGGATGGCTNAGGGGNGCTCCTGNCCTCGTGGTTACAACAACT 120

***** * * *

REV_AGM_CON_SEQ_1 ---------------------------------------------GATGCCTACCAGCTG 27

MV_589246-1095_93_HMBSrevH11.ab1 NNNNNNACAGGGTTANTGGAACGCCTATTGGACAAGTCTGAGTCTCACTGCAGCAACATG 180

* * * * **

REV_AGM_CON_SEQ_1 TGGGTCATCCTCAGGGCCATCTTCATGCTGT--ATGCGGGAAGGAGGTGGG-AATTGGTG 84

MV_589246-1095_93_HMBSrevH11.ab1 TGGGCCGCCAANNACCACAGCTTCGNNTCATGCCTGACANAGGGAGGATCGCTAGGATGG 240

**** * * ** **** * ** * ***** * * *

REV_AGM_CON_SEQ_1 AGAACAAATGAGATTATATGCACTCNTGTTTA--TTACCCCCTCACCCTCCAGCTTTGGT 142

MV_589246-1095_93_HMBSrevH11.ab1 AGAGCTNCTTTGCTTNTTCCTATTTAAGTGTNAGGGATGCCCCTGCTCCCCAC-GTTGGG 299

*** * * * ** * * * ** * * *** * * *** ****

REV_AGM_CON_SEQ_1 ACCTGGG----CAGGGACATGGATGGTAGCCTGCATGGTGTCTTGTATGCTATC------ 192

MV_589246-1095_93_HMBSrevH11.ab1 GCTAATGCATGCGGTGCAAGANATGGACGCTTCCTCTGNGCCTTCCCTCAGCTCTTCCTT 359

* * * * * * **** ** * * * * *** * **

REV_AGM_CON_SEQ_1 --------TGAGCCATCTAGACTCCAGACTCCTCCAGT---------------------- 222

MV_589246-1095_93_HMBSrevH11.ab1 TGAGTTGCTATCACACCAAGACTCTGTGATAAGCCCAAACANNNNCTTTCCTGTCTCTCT 419

* ** * ****** * **

REV_AGM_CON_SEQ_1 ------------------------------------------------------------ 222

MV_589246-1095_93_HMBSrevH11.ab1 TNAGGANGTATCNCCNTGNNNCNNCGAAGCTTCGCGCGNNTNNCGCNCGGANGNNTNCNC 479

REV_AGM_CON_SEQ_1 ------------------------------------ 222

MV_589246-1095_93_HMBSrevH11.ab1 NGAGGNGGNANNNNNNNNGNNTNNNNNNNNNANNNN 51

Mosquito 94

REV_AGM_CON_SEQ_1 ------------------------------------------------------------ 0

MV_589246-1096_94_HMBSrevH12.ab1 NNNNNNNNNNNNGNNNNNNNGCNCANCTTTNATTNANANATNATCAGTGGTGTGACTGTA 60

REV_AGM_CON_SEQ_1 -----------GTTACGAGCAGTGATGCCTACCAGCTGTGG-GTCATCCTCAGGGCCATC 48

MV_589246-1096_94_HMBSrevH12.ab1 NCTTCGATACACTTTGTAAATGTGATAATG-GCAGCTGTTGATTACTTTTCTCCAGCAGA 119

** * ***** ******* * * * ** **

REV_AGM_CON_SEQ_1 TTCATGCTGTATGCGGGAAGGAGGTGGGA-ATTGGTGAGAA------------CAAATGA 95

MV_589246-1096_94_HMBSrevH12.ab1 CTCTTCTAGTACTCTGGAGTGGTGTGACTCATCTCTCAGTACCAGACTCCAGACTCCTCC 179

** * *** * *** * *** ** * ** * * *

REV_AGM_CON_SEQ_1 GATTATATGCACTCNTGTTTATTACCCCCT---CACCCTCCAGCTTT-GGTACCTGGGCA 151

MV_589246-1096_94_HMBSrevH12.ab1 AGTCAGGTAANCTCCTTACTCCTCCCCTCCCGTAAACTCCCTACTCCTCCAGTCAGGTAA 239

* * * *** * * * *** * * * ** ** * ** *

REV_AGM_CON_SEQ_1 GGGACATGGATGGTAG------------------------------------------CC 169

MV_589246-1096_94_HMBSrevH12.ab1 GGAATGGATATGCTGTAGGCGTTGGATTCCTGTCGAGGTAAAGATTCAAATTANNAAATA 299

** * *** *

REV_AGM_CON_SEQ_1 TGCATGGTGTCTTG-----------TATGCTATCTGAGCCATCTAGACTCCAGACTCCTC 218

MV_589246-1096_94_HMBSrevH12.ab1 TGGATGGTTTTTTGTTGTGATTGACTCACTGTAGATATCCGTCCAGACTCCAGACTCCTC 359

** ***** * *** * * ** ** ****************

REV_AGM_CON_SEQ_1 CAGT--------- 222

MV_589246-1096_94_HMBSrevH12.ab1 CAATCAGGTAANN 372

** *

Mosquito 96

REV_AGM_CON_SEQ_1 ---------------------GTTACGAGCAGTGATGCCTACCAGCTGTGGG-TCATCCT 38

MV_589246-2007_96_HMBSrev_A07.ab1 NNNNNNNNNNNNNNNANNNNNNNTCNCNTNNNNGAANCANNNNNCCNGAGGCGNAAACCT 60

* ** * * * ** * ***

REV_AGM_CON_SEQ_1 CAGGGC----------------------------C-ATCTTCATGCTGTATGCGGGAAGG 69

MV_589246-2007_96_HMBSrev_A07.ab1 AAACTCCGAGTCCCGAGGCAANAGGCTAGAGGCCNCAGGTGATTACTTTTCNCCNGAAGN 120

* * * * * ** * * ****

REV_AGM_CON_SEQ_1 AGGTGGGAATTGGTGAGAACAAATGAGAT------------------------TATATGC 105

MV_589246-2007_96_HMBSrev_A07.ab1 NCT-TNNCATAAGCCGGACTGCAGACTCTTCTCTCTCAGTAACNGACTCCAGACTCCTCC 179

** * ** * * * *

REV_AGM_CON_SEQ_1 ACTCNTGTTTATTACC---CCCTCACCCTCCAGCTTTGGTACCTGGGCAGGGACATGGAT 162

MV_589246-2007_96_HMBSrev_A07.ab1 AGTCAGGTAANGTAACNNGGNCTCATCTCTTGGTTGTGGTTTCGGGGACCATTNGGGNAA 239

* ** ** ** * **** * * * **** * *** * *

REV_AGM_CON_SEQ_1 GGTAGCCTGCATGGTGTCTTGTATGCTATCTGAGCCATCTAGACTCCA----GACTC--- 215

MV_589246-2007_96_HMBSrev_A07.ab1 NGTGCCATACNNGGTGTGATGTGCTCTTTTTCTGCGAATCATCNNNNCGTTNAACGCACN 299

** * * * ***** *** ** * * ** * * ** *

REV_AGM_CON_SEQ_1 --CTCCAGT--------------------------------------------- 222

MV_589246-2007_96_HMBSrev_A07.ab1 TGCTGCCTTTGTTATTCTGGAGGGCATGCCTGTTCCGNTGGCTTGTTTGGTCGC 353

** * *

Mosquito 97

REV_AGM_CON_SEQ_1 ------------------------------------------------------------ 0

MV_589246-2008_97_HMBSrev_A08.ab1 NNNNNNNNTTTCNNGCGTAACTTTCCNTTAAGACCAGCATCAGCAGTGTGGCGTAACTTC 60

REV_AGM_CON_SEQ_1 ------GTTACGAGCAGTGATGCCTACCAGCTGTGGGTCATCCTCAGGGCCATCTTCATG 54

MV_589246-2008_97_HMBSrev_A08.ab1 GATACACTTTAGCGATGTGATAATGGCAGCTGTTGATTACTTTTCTCCAGCAGACTCTTC 120

** * * ***** * ** * * ** ** ** *

REV_AGM_CON_SEQ_1 CTGTATGCGGGAAGGAGGTGGGA-ATTGGTGAGAACAA--ATGAGATTATATGCACTCNT 111

MV_589246-2008_97_HMBSrev_A08.ab1 TAGTACTCTGGAGTGGTGTGACTCATCTCTCAGTACCAGACTCCAGACTCCTCCAGTCAG 180

*** * *** * *** ** * ** ** * * * ** **

REV_AGM_CON_SEQ_1 GTTTATTACCCCCTCACCCTCCAGCTTTGGTACCTGGGCAGGGACATGGATGGTAGCCTG 171

MV_589246-2008_97_HMBSrev_A08.ab1 GTAAACTCCANAC---TCCTCCA-GTCAGGTAAGCGGNNNCGGN------------NNNT 224

** * * * * ****** * **** ** **

REV_AGM_CON_SEQ_1 CATGGTGTCTTGTATGCTATCTGAGCCATCTAGACTCCAGACTC---------------- 215

MV_589246-2008_97_HMBSrev_A08.ab1 CNNAACGGTNTNTGTGATATGAGGGATATCGAGGCTGCGNAGTCNGGAAATATATCAANT 284

* * * * ** *** * * *** ** ** * * **

REV_AGM_CON_SEQ_1 ------------------------------------------------------------ 215

MV_589246-2008_97_HMBSrev_A08.ab1 GACTCCNACTCCNACTCNNTNTCACGCTTTCNGGAATTAGCGNACTCCNGGTCCGACTCC 344

REV_AGM_CON_SEQ_1 -----------------------------------------CTCCAGT------------ 222

MV_589246-2008_97_HMBSrev_A08.ab1 GAATCAGGNTTGCNTATCCGAGNGNNTCATAGTCTTNACTCNTACTGTCAGGTAAAGGAC 404

* * **

REV_AGM_CON_SEQ_1 -------------------------- 222

MV_589246-2008_97_HMBSrev_A08.ab1 TGGGAGGCGGCCNANCCGGTNTNGGA 430

Mosquito 98

REV_AGM_CON_SEQ_1 GTTACGAGCAGTGATGCCTACCAGCTGTGGGTCATCCTCAGGGCCATCTTCATGCTGTAT 60

MV_589246-2009_98_HMBSrev_A09.ab1 -NNNNNNNNNNNGTTTCGGCGCAACTTTCCATTAAGACCAGCATCAGCAGTGTGGCGTAA 59

* * * ** ** * * * *** ** * ** ***

REV_AGM_CON_SEQ_1 GCGGGAAGGAGGTGGGAATTGGTGAGAACAAATGAGATTATATGCACTCNTGTTTATTAC 120

MV_589246-2009_98_HMBSrev_A09.ab1 CTT------CGATACACTTTAG-------CGATGTGATAATGGCAGCTGTTGATTACTTT 106

* * ** * *** *** ** ** ** *** *

REV_AGM_CON_SEQ_1 CCCCTCACCCTCCAGCTTTGGTACCTGGGCAGGGACATGGATGGTAGCCTGCATGGTGTC 180

MV_589246-2009_98_HMBSrev_A09.ab1 TCTCCAGCAGAC-TCTTCTAGTA-CTCTGGAGTGGTGTGACTCAT-CTCTCAGTACCAGA 163

* * * * * * *** ** * ** * ** * * ** *

REV_AGM_CON_SEQ_1 TTGTATGCTATCTGAGCCATCTAGACTCCAGACTCCTCCAGT------------------ 222

MV_589246-2009_98_HMBSrev_A09.ab1 CTCCAGACTCCTCCAGTCAGGTAAACTCCAGACTCCTCCAGTCAGGTAAGNGGTCCCGAA 223

* * ** ** ** ** ******************

REV_AGM_CON_SEQ_1 ------------------------------------------------------------ 222

MV_589246-2009_98_HMBSrev_A09.ab1 GACTCCNNACTCCTCNNGTCNGGTACAANTTTGCNATGCACTCCTGNGTGGGTAANNAAA 283

REV_AGM_CON_SEQ_1 ------------------------------------------------------------ 222

MV_589246-2009_98_HMBSrev_A09.ab1 CCAATTAATAGACATGGGNGNTTNTTGNTCTTCAACAGAGGNNGAAGAGATCAGATGTCN 343

REV_AGM_CON_SEQ_1 ------------------------------------------------------------ 222

MV_589246-2009_98_HMBSrev_A09.ab1 TACNGAACACTGAGCCTAGCGGGTNNNNNGGGAGGNNTTGNGTTTTATTAGAAANGTGTN 403

REV_AGM_CON_SEQ_1 ------------------------------ 222

MV_589246-2009_98_HMBSrev_A09.ab1 ATNGGTGNTGGTTGGGGGGAGGGGCAGGTN 433

Mosquito 99

REV_AGM_CON_SEQ_1 GTTACGAGCAGTGATGCCTACCAGCTGTGGGTCATCCTCAGGGCCATCTTCATGCTGTAT 60

MV_589246-2010_99_HMBSrev_A10.ab1 NNNNNNNNNNNNGTTTCGGCGCNNCTTTCCNTT------AAGACCAGCATCAGCAGTGTG 54

* * * * ** * * * * *** * ***

REV_AGM_CON_SEQ_1 GCGGGAAGGAGGTGGGAATTGGTGAGAACAAATGAGATTATATGCACTCNTGTTTATTAC 120

MV_589246-2010_99_HMBSrev_A10.ab1 GCGTAACTTCGATACACTTTAG-------CGATGTGATAATGGCAGCTGTTGATTACTTT 107

*** * * * ** * *** *** ** ** ** *** *

REV_AGM_CON_SEQ_1 CCCCTCACCCTCCAGCTTTGGTACCTGGGCAGGGACATGGATGGTAGCCTGCATGGTGTC 180

MV_589246-2010_99_HMBSrev_A10.ab1 TCTCCAGCAGAC-TCTTCTAGTA-CTCTGGAGTGGTGTGACTCAT-CTCTCAGTACCAGA 164

* * * * * * *** ** * ** * ** * * ** *

REV_AGM_CON_SEQ_1 TTGTATGCTATCTGAGCCATCTAGACTCCAGACTCCTCCAGT------------------ 222

MV_589246-2010_99_HMBSrev_A10.ab1 CTCCAGACTCCTCCAGTCAGGTAAACTCCAGACTCCTCCAGTCAGGTAAANGGCNNGGAN 224

* * ** ** ** ** ******************

REV_AGM_CON_SEQ_1 ------------------------------------------------------------ 222

MV_589246-2010_99_HMBSrev_A10.ab1 ANTGTGNGCGNCCGNGTGNACCAGGNNNANNAAGCCNTCTNATCCGGAAAAAATNGCAGT 284

REV_AGM_CON_SEQ_1 ------------------------------------------------------------ 222

MV_589246-2010_99_HMBSrev_A10.ab1 CACCCAGACTGTGTGGNNAGTNGTGANAAGGGGNNGACGGANAAACCCNCTTNTANTGCT 344

REV_AGM_CON_SEQ_1 ------------------------------------------------------------ 222

MV_589246-2010_99_HMBSrev_A10.ab1 GTANGNTTTTTTGNCGAATGGNGGCNGACTCCAAAANCAAAANNNTGTAANNNATGGTGG 404

REV_AGM_CON_SEQ_1 ------------------------------------------------------------ 222

MV_589246-2010_99_HMBSrev_A10.ab1 CGGGGGGGGAGGTGGCGCAGGANAGATTAGGGGTCGANAGACNNGAATGATTTTTTCTCG 464

REV_AGM_CON_SEQ_1 ------------------------------------------------------------ 222

MV_589246-2010_99_HMBSrev_A10.ab1 TAANATGNNGACNNCGGGATGGGAAGTNCCCNTNGGCNNNNAGANNANAAGGGTTGCTGA 524

REV_AGM_CON_SEQ_1 ------------ 222

MV_589246-2010_99_HMBSrev_A10.ab1 GGGAAGTTTTTN 536

Mosquito 100

REV_AGM_CON_SEQ_1 ----------------------------GTT---------ACGAGCAGTGATGCCTACC- 22

MV_589246-2011_100_HMBSrev_A11.ab1 NNNNNNNNNNNGTTTCGGCGCACTTTCCATTAAGACCAGCATCAGCAGTGTGGCGTAACT 60

** * ******* ** ** *

REV_AGM_CON_SEQ_1 -----------------------------AGCTGT-GGGTCATCCTCAGGGCCATCTTCA 52

MV_589246-2011_100_HMBSrev_A11.ab1 TCGATACACTTTAGCGATGTGATAATGGCAGCTGTTGATTACTTTTCTCCAGCAGACTCT 120

****** * * * ** ** **

REV_AGM_CON_SEQ_1 TGCTGTATGCGGGAAGGAGGTGGGA-ATTGGTGAGAACAA--ATGAGATTATATGCACTC 109

MV_589246-2011_100_HMBSrev_A11.ab1 TCTAGTACTCTGGAGTGGTGTGACTCATCTCTCAGTACCAGACTCCAGACTCCTCCAGTC 180

* *** * *** * *** ** * ** ** * * * ** **

REV_AGM_CON_SEQ_1 NTGTTTATTACCCCCTCACCCTCCAGCTTTGGTACCTGGGCAGGG--------------- 154

MV_589246-2011_100_HMBSrev_A11.ab1 AGGTAAACTCC----NACTCCTCCAGTCAGGTAAAGCGGTCNGACACTCNNCNCGGACTC 236

** * * * ******* * * ** * *

REV_AGM_CON_SEQ_1 -----------------------------------------------ACATGGATGGTAG 167

MV_589246-2011_100_HMBSrev_A11.ab1 NNGTAATTTCGTAAACCGACCCCAGANNCCAGAGTCCTCCGCCTCCTTCATNNATGTAA- 295

*** *** *

REV_AGM_CON_SEQ_1 CCTGCATGGTGTCTTGTATGCTATCT---------------------------------- 193

MV_589246-2011_100_HMBSrev_A11.ab1 TCACNCGGTTCTATTTCCGACTTTCCCAGCTAACCGCTGCGCCGCCNAATTCCAATCCGC 355

* * * * ** ** **

REV_AGM_CON_SEQ_1 ------------GAGCCATCTAGACTCCAGACTCCTCCAGT------------------- 222

MV_589246-2011_100_HMBSrev_A11.ab1 TCGNNAACTTCCGGTCGACNNTNACTCCGGACTCCTCCATGCAAGTAAAACCTGGGCATC 415

* * * ***** **********

REV_AGM_CON_SEQ_1 ------------------------------------------------------------ 222

MV_589246-2011_100_HMBSrev_A11.ab1 NGCANGGGTTCTGGATANTGCTGCCCTNCGGNAANANCGCCTAAAGCCTGNGNGGAANCC 475

REV_AGM_CON_SEQ_1 ------------------------------- 222

MV_589246-2011_100_HMBSrev_A11.ab1 TGGGCATAGACGAAGAGTTTTTGGGCNTGTA 506

Mosquito 101

REV_AGM_CON_SEQ_1 GTTAC---GAGCAGTGATGCCTACC-----AGCTGTGGGTCATCCTCAGGGCCATCTTCA 52

MV_589246-2012_101_HMBSrev_A12.ab1 GNNNNNNNNNNNNNNGNNNNNNNANAACNGNNNACTCCANACTCCTCCAGTCAGGTAANN 60

* * * ***** * *

REV_AGM_CON_SEQ_1 TGCTGTATGCGGGAAGGAGGTGGGAATTGGTGAGAACAAATGAGATTATATGCACTCNTG 112

MV_589246-2012_101_HMBSrev_A12.ab1 CACTAACTTCGATAC--------------AGTAANAGCGATGTGATAATGGCAGCTGTTG 106

** * ** * * * *** *** ** ** **

REV_AGM_CON_SEQ_1 TTTATTACCCCCTCACCCTCCAGCTTTGGTACCTGGGCAGGGACATGGATGGTAGCCTGC 172

MV_589246-2012_101_HMBSrev_A12.ab1 ATTACTTTTCTCCAGCAGAC-TCTTCTAGTAC-T-------------------------- 138

*** * * * * * * * **** *

REV_AGM_CON_SEQ_1 ATGGTGTCTTGTATGCTATCTGAGCCATCTAGACTCCAGACTCCTCCAGT---------- 222

MV_589246-2012_101_HMBSrev_A12.ab1 CTGGAGTGGTGTGACTCATCTCTCAGTACCAGACTCCAGACTCCTCCAGTCAGGTAAATT 198

*** ** *** **** * ********************

REV_AGM_CON_SEQ_1 ------------------------------------------------------------ 222

MV_589246-2012_101_HMBSrev_A12.ab1 TTAGAGAATTGGNANTTAGGGTTAAAGAAGTTTTCCCGAGNATTTTNTTTGNAANTTGNT 258

REV_AGM_CON_SEQ_1 ------------------------------------------------------------ 222

MV_589246-2012_101_HMBSrev_A12.ab1 TTTNNNATNTNCTAATGTTTCCNANAATTTTTTTTGNCNGGTGNCAAACTCNGNATTCCT 318

Mosquito 102

REV_AGM_CON_SEQ_1 ------------------------------------------------------------ 0

MV_589246-2013_102_HMBSrev_B01.ab1 NNNNNNNNNNGGNNNNNNTTNNTNCNNNNNANNNNNGNGANAAGTTGTTNCGCCACATAA 60

REV_AGM_CON_SEQ_1 ---------------------------------------GTTACGAGCAGTGATGCCTAC 21

MV_589246-2013_102_HMBSrev_B01.ab1 ACANGAAACAGGNTTTCTTTATANANAATCTGTCAATGGGATNTCCCCGATGGTTCCA-A 119

* * * ** * **

REV_AGM_CON_SEQ_1 CAGCTGTGGGTCATCCTCAGGGCCATC---TTCATGCTGTATGCGGGAAGGAGGTGG--- 75

MV_589246-2013_102_HMBSrev_B01.ab1 CAACAGATTGTGATTCTCATCAGCTTCTGCAACATGAGCTGTGGGAGCAAACATCCTCAT 179

** * * ** ** **** * ** **** * ** * * *

REV_AGM_CON_SEQ_1 ---------GAATTGGTGAGAACAAATGAGATTATA-----TGCACTCNTGTTTATTACC 121

MV_589246-2013_102_HMBSrev_B01.ab1 TGGCTGGTGGAGAGGTGGAGAACGATCCACCTCTCCAAACCTCCTCTGATCTGGCTACAA 239

** * ****** * * * * * ** * * *

REV_AGM_CON_SEQ_1 CCCTCACCC--------------------------------------------------- 130

MV_589246-2013_102_HMBSrev_B01.ab1 CCAGCAGCCATGGACTAGACTCCAGACTCCTCCAGTCAGGTAAAGTCCTNCTCCCTGCTN 299

** ** **

REV_AGM_CON_SEQ_1 -----TCCAGCTTTGGTACC---------------------------------------- 145

MV_589246-2013_102_HMBSrev_B01.ab1 AAGCAGCTCCCTACAGTANCTTGCAAAGGACANCATCCNNTNGGGTTTTGAATCTCCCCG 359

* ** *** *

REV_AGM_CON_SEQ_1 ------------------------------------TGGGCAGGGACATGGATGGTAGCC 169

MV_589246-2013_102_HMBSrev_B01.ab1 NANANNGAGACTCCGACTCCTCCAGTCAAGTAAGACTGTGCATCGAATGGGGTAGGAGGC 419

** *** ** ** * * ** *

REV_AGM_CON_SEQ_1 TGCATGGTGTCTTGTATGCTATCTGAGCCATCTAGACTCCAGACTCCTCCAGT------- 222

MV_589246-2013_102_HMBSrev_B01.ab1 TTCNAGGNNTCCTGGGGCCNTC--------CTGAGACTCCNNNCTCCTCCAGTCAGGTAA 471

* * ** ** ** * ******* **********

REV_AGM_CON_SEQ_1 ---- 222

MV_589246-2013_102_HMBSrev_B01.ab1 NGTG 475

Mosquito 103

REV_AGM_CON_SEQ_1 ------------------------------------------------------------ 0

MV_589246-2014_103_HMBSrev_B02.ab1 NNNNNNNNNNNNNNTNNNNTGNNNNNNNNNNGTGNNNGGNTGATGACTGCNNTCNNATTT 60

REV_AGM_CON_SEQ_1 --------GTTACGAGCAGTGATGCCTAC---CAGC-TGTGGGTCATCCTCAGGGCCATC 48

MV_589246-2014_103_HMBSrev_B02.ab1 GGAACTTCTTCGCCAGCACTGGCGCGGGATGCACCTTACTTGTTCACCCNCGCNCTAGAC 120

* * **** ** ** * * *** ** * *

REV_AGM_CON_SEQ_1 TTCATGCTGTATGCGGGAAGGAGGTGGGAA------------------------------ 78

MV_589246-2014_103_HMBSrev_B02.ab1 TCCATACTCCTNGGGTCAAGTAAANNTCCAATTTTTGGCCTTGNCCTTCGNNAATGCAAC 180

* *** ** * * *** * *

REV_AGM_CON_SEQ_1 ------------------------------------------------------------ 78

MV_589246-2014_103_HMBSrev_B02.ab1 ACTCCAAACTCATCCNGTAAAGAAAATAACACGGGGACACCTNCCTNCGGGTAACTNNGN 240

REV_AGM_CON_SEQ_1 ------------------------------------------------------------ 78

MV_589246-2014_103_HMBSrev_B02.ab1 GTNCCNNATGGANNCCNCTCCCTCNAACACCCCCGGAGCCTCTCCCCTCTCACTGCTGCC 300

REV_AGM_CON_SEQ_1 --------------------------------------------------------TTGG 82

MV_589246-2014_103_HMBSrev_B02.ab1 GTCCNCACCCAGAGAACCTCCGNAGACTCCAGACTCCTCCGGTCAGGTAAANNNTTCTAN 360

*

REV_AGM_CON_SEQ_1 TGAGAACAAATGAGATTATATGCACTCNT-------------------------GTTTAT 117

MV_589246-2014_103_HMBSrev_B02.ab1 ANGGGGNNANTGCGCTTTNNNAGCCTCTGGAGTATCANGATNAATATTTCCCCGATGTGG 420

* * ** * ** *** * *

REV_AGM_CON_SEQ_1 TACCCCCTCACCCTCCAGCTT--------------------------------------- 138

MV_589246-2014_103_HMBSrev_B02.ab1 TATGCCAGANAACTCAAGACTCACCCNGGCCGGNAAGCGGATCATTNGNGAACGTTTTCA 480

** ** *** ** *

REV_AGM_CON_SEQ_1 -----------------------TGGTACCTGGGCAGGGACATGGATGGTA--------G 167

MV_589246-2014_103_HMBSrev_B02.ab1 TGGTTGNANNNNNTTCCAANTGCTTTTACNTCGGCTTATANCTCGTTCATTGCTTGTTCT 540

* *** * *** * * * * *

REV_AGM_CON_SEQ_1 CCTGCATGGT-GTCTTGTATGCTATCTGAGCCATCTAGACTCCAGACTCCTCCAGT---- 222

MV_589246-2014_103_HMBSrev_B02.ab1 GNCGAATGATTTTCCGGATGNNNGCCTCAGAGATCAANACTCCNTACTCCTCCAGTCGCG 600

* *** * ** * ** ** *** * ***** ***********

REV_AGM_CON_SEQ_1 --- 222

MV_589246-2014_103_HMBSrev_B02.ab1 TAA 603

Mosquito 104

REV_AGM_CON_SEQ_1 -----------------------GTTA---------CGAGCAGTGATGCCTACC------ 22

MV_589246-2015_104_HMBSrev_B03.ab1 NNNNNNNNTTCGGCGCNNTTTCCNTTAAGACCAGCATCAGCAGTGTGGCGTAACTTCGAT 60

*** ******* ** ** *

REV_AGM_CON_SEQ_1 ------------------------AGCTGT-GGGTCATCCTCAGGGCCATCTTCATGCTG 57

MV_589246-2015_104_HMBSrev_B03.ab1 ACACTTTAGCGATGTGATAATGGCAGCTGTTGATTACTTTTCTCCAGCAGACTCTTCTAG 120

****** * * * ** ** ** * *

REV_AGM_CON_SEQ_1 TATGCGGGAAGGAGGTGGGA-ATTGGTGAGAACAA--ATGAGATTATATGCACTCNTGTT 114

MV_589246-2015_104_HMBSrev_B03.ab1 TACTCTGGAGTGGTGTGACTCATCTCTCAGTACCAGACTCCAGACTCCTCCAGTCAGGTA 180

** * *** * *** ** * ** ** * * * ** ** **

REV_AGM_CON_SEQ_1 TATT------------------------ACCCCCTCACCCTCCAGCTTTGGTACCTGGGC 150

MV_589246-2015_104_HMBSrev_B03.ab1 AACTCCAGACTCCTCCAGTCAGGTAANNATGTGTTCNNTCTCNATCTCNGGTAGNNNGTA 240

* * * ** *** * ** **** *

REV_AGM_CON_SEQ_1 AGGGACATGGATGGTAGC---CTGCATGGTGTC--------------------------- 180

MV_589246-2015_104_HMBSrev_B03.ab1 GTTGAAATTTAGGCTCTGGTACTGCAGGGTTTTTANTTTCTAGATGTGGGAAATGTTCAG 300

** ** * * * ***** *** *

REV_AGM_CON_SEQ_1 ---------------------------------------------------------TTG 183

MV_589246-2015_104_HMBSrev_B03.ab1 TCNGACGAAATCGGTCTGGGACCGGTCCCGGTTTAGGACGGAGTCCGATTTGACTAAACC 360

REV_AGM_CON_SEQ_1 TATGCTATCTGAGCCATCTAGACTCCAGACTCCTCCAGT---------- 222

MV_589246-2015_104_HMBSrev_B03.ab1 TTCCATGTTGTGTTCCTTGANACTCCAGACTCGTCAAGTCAGGTAAAGG 409

* * * * * * *********** ** ***

Mosquito 105

REV_AGM_CON_SEQ_1 ----------------------------------------GTTACGAGCAGTGATGCCTA 20

MV_589246-2016_105_HMBSrev_B04.ab1 NNNNNNNNNNNNGTTTCNNGCGCANCTTTCCNTTAAGACCAACATCAGCAGTGTGGCGTA 60

* ******* ** **

REV_AGM_CON_SEQ_1 CC------------------------------AGCTGT-GGGTCATCCTCAGGGCCATCT 49

MV_589246-2016_105_HMBSrev_B04.ab1 ACTTCGATACACTTTAGCGATGTGATAATGGCAGCTGTTGATTACTTTTCTCCAGCAGAC 120

* ****** * * * ** **

REV_AGM_CON_SEQ_1 TCATGCTGTATGCGGGAAGGAGGTGGG-AATTGGTGAGAACA--AATGAGATTATATGCA 106

MV_589246-2016_105_HMBSrev_B04.ab1 TCTTCTAGTACTCTGGAGTGGTGTGACTCATCTCTCAGTACCAGACTCCAGACTCCTCCA 180

** * *** * *** * *** ** * ** ** * * * **

REV_AGM_CON_SEQ_1 CTCNTGTTTATTACCCCCTCACCCTCCAGCTTTGGTACCTGGGCAGGGACATGGATGGTA 166

MV_589246-2016_105_HMBSrev_B04.ab1 GTCAGGTNNTTTTTCCGGAAACCCTAG-------CAAACGGTTCAAGAG---TGGTGGTT 230

** ** ** ** ***** * * * ** * * ****

REV_AGM_CON_SEQ_1 GCCTGCATGGTGTCTTGTATGCTATCTGAGCCATCTAGACTCCAGACTCCTCCAGT---- 222

MV_589246-2016_105_HMBSrev_B04.ab1 GCCGCCA--------------ACTGTGGGTCTGCGCAGACTCCAGACTCCTCCAGTCAGG 276

*** ** * * ********************

REV_AGM_CON_SEQ_1 ------------------------------------------------------------ 222

MV_589246-2016_105_HMBSrev_B04.ab1 TAAAAACCGCATCNCNTGTAAGANCTTTTNTNTNNTGGATCATCTCTGTTGANGGCTGGA 336

REV_AGM_CON_SEQ_1 ------------------------------------------------------------ 222

MV_589246-2016_105_HMBSrev_B04.ab1 GGCCTACTTTCGNAGTTGGGTTGTCCNAACTGATAAATTAGTATCTGTAAGATNANNCTA 396

REV_AGM_CON_SEQ_1 --------------------------------------- 222

MV_589246-2016_105_HMBSrev_B04.ab1 CNGNGAGTTTGGGGATTGNTGATAGTGACTGCGGACTGG 435

Mosquito 106

REV_AGM_CON_SEQ_1 ------------------GTTACGAGCAGTGATGCCTACCAGCTGTGGGTCATCCTCAGG 42

MV_589246-2017_106_HMBSrev_B05.ab1 NNNNNNNNNNNCNNNGNTGTTACGAGCAGTGATGCCTACCAACTGTGGGTCATCCTCAGG 60

*********************** ******************

REV_AGM_CON_SEQ_1 GCCATCTTCATGCTGTATGCGGGAAGGAGGTGGGAATTGGTGAGAACAAATGAGATTATA 102

MV_589246-2017_106_HMBSrev_B05.ab1 GCCATCTTCATGCTGTATGAGGGAAGGAGGTGGGATTTGGTGAGAACAA--GAGATTATA 118

******************* *************** ************* *********

REV_AGM_CON_SEQ_1 TGCACTCNTGTTTATTACCCCCTCACCCTCCAGCTTTGGTACCTGGGCAGGGACATGGAT 162

MV_589246-2017_106_HMBSrev_B05.ab1 TGCACTCTTGTTTATTACCCCCTCGCCCTCCAGCTTTGGTACCTGGGCAGGGACATGGAT 178

******* **************** ***********************************

REV_AGM_CON_SEQ_1 GGTAGCCTGCATGGTGTCTTGTATGCTATCTGAGCCATCTAGACTCCAGACTCCTCCAGT 222

MV_589246-2017_106_HMBSrev_B05.ab1 GGTAGCCTGCATGGTCTCTTGTATGCTATCTGAGCCGTCTAGACTCCAGACTCCTCCAGT 238

*************** ******************** ***********************

REV_AGM_CON_SEQ_1 ------------------------------------------------------------ 222

MV_589246-2017_106_HMBSrev_B05.ab1 CAGGTAAAGNNATGGCAGTCCCCATTTCTCCAATCAGGTAAAATGNCCCCCCCCTCGGCT 298

REV_AGM_CON_SEQ_1 ------------------------------------------------------------ 222

MV_589246-2017_106_HMBSrev_B05.ab1 GGTTTCTTTCTCCTTCTGGGGCTCCAGGACANGACCCCCTTGCATTGCAAGGAAATCCCC 358

REV_AGM_CON_SEQ_1 ------------------------------------------------------------ 222

MV_589246-2017_106_HMBSrev_B05.ab1 CATCTCAAACAGCGCATGCCTCAACTGTGGGGAGGCCATGAAACGTGGGATCAGAACCTG 418

REV_AGM_CON_SEQ_1 ------------------ 222

MV_589246-2017_106_HMBSrev_B05.ab1 GGCATCAGCAAGGCANNN 436

Mosquito 107

REV_AGM_CON_SEQ_1 ------------------------------------------------------------ 0

MV_589246-2018_107_HMBSrev_B06.ab1 NNNNNNNNNNNCNGNTNNNNNNNNNTGCACTGCTGGCAGCTNNNGCNNANNTTTGGNGTG 60

REV_AGM_CON_SEQ_1 ----------------GTTACGAGCA-----------------------------GTGAT 15

MV_589246-2018_107_HMBSrev_B06.ab1 CGTGCCCTCCCCAGATCTTATGAGGACCTGGGGGACTCGGATGGGCTTGGGGGGGTGGCT 120

*** *** * * *

REV_AGM_CON_SEQ_1 GCCTACCAGCTGTGGGTCATCCTCAGGGCCATCTTCATGCTGTATGCGGGAAGGAGGTGG 75

MV_589246-2018_107_HMBSrev_B06.ab1 CANGTCCTGTGATGGAGCACGCTCAGCACGGTAANTCAGCTCAGGGGCAGACAGACTCCA 180

** * *** ** ***** * * *** * ** **

REV_AGM_CON_SEQ_1 GAATTGGTGAGAACAAATGAGA-TTATATGCACTCNTGTTTATTACCCCCTCACCCTCCA 134

MV_589246-2018_107_HMBSrev_B06.ab1 GACTCCT-CCAGTCAGGTAANNGNTTGCTGTCCT--NGGCTNTTTTCTCCTTTATCTGCT 237

** * ** * * * ** ** * * ** * *** ** *

REV_AGM_CON_SEQ_1 GCTTTGGTACCTGGGCAGGGACATGGATGGTAGCCT---GCATGGTGTCTTG-------T 184

MV_589246-2018_107_HMBSrev_B06.ab1 GCTTGGGNAANCAATAAGAN--ATAGCTGCACCATTACTGACAACTCTATTTGAGAGCCG 295

**** ** * ** ** * ** * * * * **

REV_AGM_CON_SEQ_1 ATGCTATCTGAGCCATCTAGACTCCAGACTCCTCCAGT---------------------- 222

MV_589246-2018_107_HMBSrev_B06.ab1 TTGTCTGAAACAGNNTCAANACTCCAGACTCCTCCAGTCAGGTAANTCNNTTACTGCTTG 355

** ** * ******************

REV_AGM_CON_SEQ_1 ------------------------------------------ 222

MV_589246-2018_107_HMBSrev_B06.ab1 NTCGCCCTTTGTCATCANGNAGNCGGANTGGGCTCCTATTNA 397

Mosquito 108

REV_AGM_CON_SEQ_1 GTTACGAGCAGTGATGCCTACCAGCTGTGGGTCATCC----------------------- 37

MV_589246-2019_108_HMBSrev_B07.ab1 NNNNNNNNNNNNNCGCNCTANNAGCCNTTTGTAACTGTAGTATACCTATACGATAGTAAT 60

*** *** * ** *

REV_AGM_CON_SEQ_1 -----------TCAGGGCCATCTTCATGCTGTATGCGGGAAGGAGGTGGGAAT------- 79

MV_589246-2019_108_HMBSrev_B07.ab1 GTGTGCACGAAGATCCATTGTCTTCGTGCGATGTTCTACCACATGGTAGAAATATTGCTC 120

***** *** * * * * *** * ***

REV_AGM_CON_SEQ_1 -----TGGTG-------------------------------------------------- 84

MV_589246-2019_108_HMBSrev_B07.ab1 TTTATCTGTGATATCAATAAACGACTAGGAGTAGTTCATGTTCAGACTCCAGACTCCTCC 180

***

REV_AGM_CON_SEQ_1 ------------------------------------------------------------ 84

MV_589246-2019_108_HMBSrev_B07.ab1 AGTCAGGTAAAACGGCAACAGACCTTANACTCCAGACTCCTCCAGTCAGGTAAACNAGAC 240

REV_AGM_CON_SEQ_1 --------------AGAACAAATGAGATTATATGCACTCNTGTTTATTACCCCCTCACCC 130

MV_589246-2019_108_HMBSrev_B07.ab1 NCCTTCGCCTNCAGNGAACTAANAGGAGCCNTTNGAAACAGNNNANCTACCCCGGTGCNC 300

**** ** ** * * * ****** * *

REV_AGM_CON_SEQ_1 TCCAGCTTTGGTAC---------------------------------------------- 144

MV_589246-2019_108_HMBSrev_B07.ab1 ACCGACTCCTNTNCCTNACCNTGACTGGCTCNTCNNATCCCGTACTCCCATGTAGACTGC 360

** ** * *

REV_AGM_CON_SEQ_1 ------------------------------------------------------------ 144

MV_589246-2019_108_HMBSrev_B07.ab1 ATAAAGTCNACNTCTACCCCAAGCTATCCGGGTTGCGCNAAAGTTTCCANATGGCTTTNC 420

REV_AGM_CON_SEQ_1 ------------------------------------------------------------ 144

MV_589246-2019_108_HMBSrev_B07.ab1 TTTTGANTTGCCGGNATGATTCCTNTCNCTNNANNNGTACTGCGATTTCATCACCCCGNG 480

REV_AGM_CON_SEQ_1 -----------------------CTGGGCAGGGACATGGATGGTAGCC---TGCATGGTG 178

MV_589246-2019_108_HMBSrev_B07.ab1 TAACNTCACCGAATTGTGGGCCGATATCNATGGNGATCGGCGGTTGGNNGCTGCGTNCGG 540

* * ** ** * *** * *** * *

REV_AGM_CON_SEQ_1 TCTTGTATGCTATCTGAGCCA-----------TCTAGACTCCAGACTCCTCCAGT----- 222

MV_589246-2019_108_HMBSrev_B07.ab1 TCTNNGGAGTTTTTTCCGCNAAAATACTTCTTNCTGAAAACAATACTCACCGAGTTGANT 600

*** * * * * ** * ** * * * **** * ***

REV_AGM_CON_SEQ_1 ------------------------------------------------------------ 222

MV_589246-2019_108_HMBSrev_B07.ab1 AACACACTTGTNTTGCACNTGTNCATCTTANACTCATTCGTGGGTTGAGAACAGGNGNCG 660

REV_AGM_CON_SEQ_1 -------------------------------------------------- 222

MV_589246-2019_108_HMBSrev_B07.ab1 TGATTNTGNANNNATTCTTCCTCGGAGAACCTGTGGNGCAGACTGGNANG 710

Mosquito 110

REV_AGM_CON_SEQ_1 ------------------------------------------------------------ 0

MV_589246-2021_110_HMBSrev_B09.ab1 NNNNNNNNGNAGNNNNCNNNNNCTAGCNTNNCTNNNNNACANNTCCTCACAAGTAATGTC 60

REV_AGM_CON_SEQ_1 ------------------------------------------------------------ 0

MV_589246-2021_110_HMBSrev_B09.ab1 GTAACTTCGATACACTTTAGCGATGTGATAATGGCAGCTGTTGATTACTTTTCTCCAGCA 120

REV_AGM_CON_SEQ_1 ----------GTTACGAGCAGTGATGCCTACC---------------------------- 22

MV_589246-2021_110_HMBSrev_B09.ab1 GACTCTTCTAGTACTCTGGAGTGGTGTGACTCATCTCTCAGTACCAGACTCCAGACTCCT 180

** * **** ** *

REV_AGM_CON_SEQ_1 ------AGCTGTGGGTCATCCTCA-GGGCCATCTTCATGCTGTATGCGGG-AAGGAGGTG 74

MV_589246-2021_110_HMBSrev_B09.ab1 CCAGTCAGGTAAGGGGTTTCCNGCGNGGNNGCCTCGGGTCTGGCCTCGGGGANNANNNCN 240

** * *** *** ** ** *** **** *

REV_AGM_CON_SEQ_1 GGAATTGGTGAGAACAAATGAGATTATATGCACTCNTGTTTATTACCCCCTCACCCTCCA 134

MV_589246-2021_110_HMBSrev_B09.ab1 TCAAAGTGCGAATACTAATGTGAATTGCNNAATTGTCGAATCATCNAACCTTTGAACGCA 300

** * ** ** **** ** * * * * * * *** **

REV_AGM_CON_SEQ_1 GCTTTGGTACCTGGGCAG------GGACATGGATGGT--AGCCTGCATGGTGTC------ 180

MV_589246-2021_110_HMBSrev_B09.ab1 CATTGCGCCCTTTGGGATTCCAAAGGGNNTGCCTGTTCGAGCGTCATTTGTACCCTTAAG 360

** * * * ** * ** ** ** * *** * * ** *

REV_AGM_CON_SEQ_1 --TTGTATGCTATCTGAGCCATCTAGACTCCAGACTCCTCCAGT---------------- 222

MV_589246-2021_110_HMBSrev_B09.ab1 CTTTGCTCGGTGTNGGGNGCNTNTGTCTNTCACGAGAGTCGCCTTANAATGATTGGGGCC 420

*** * * * * * * * ** ** *

REV_AGM_CON_SEQ_1 ------------------------------------------------------------ 222

MV_589246-2021_110_HMBSrev_B09.ab1 GACNTACTGGTNTCGGAGCGCAGCACAATTCTTGCACTTNGAGGANCCTTGGTTGAGCAT 480

REV_AGM_CON_SEQ_1 ------------------------------------------------------------ 222

MV_589246-2021_110_HMBSrev_B09.ab1 NNTNGNGACCACNTTTATTTGAGCTTTTGACNTCCGATCAGNGTAGGGANACGTCGCTGA 540

REV_AGM_CON_SEQ_1 -------------------------------------- 222

MV_589246-2021_110_HMBSrev_B09.ab1 GCTTAACGCATATTAATAAGNGGAGGNNNANNNANNCA 578

Mosquito 111

REV_AGM_CON_SEQ_1 ----------------------------GTT---------ACGAGCAGTGATGCCTACC- 22

MV_589246-2022_111_HMBSrev_B10.ab1 NNNNNNNNNNNGTTTCGGCGCNCTTTCCATTAAGACCAGCATCAGCAGTGTGGCGTAACT 60

** * ******* ** ** *

REV_AGM_CON_SEQ_1 -----------------------------AGCTGT-GGGTCATCCTCAGGGCCATCTTCA 52

MV_589246-2022_111_HMBSrev_B10.ab1 TCGATACACTTTAGCGATGTGATAATGGCAGCTGTTGATTACTTTTCTCCAGCAGACTCT 120

****** * * * ** ** **

REV_AGM_CON_SEQ_1 TGCTGTATGCGGGAAGGAGGTGG------------------------------------- 75

MV_589246-2022_111_HMBSrev_B10.ab1 TCTAGTACTCTGGAGTGGTGTGACTCATCTCTCAGTACCAGACTCCAGACTCCTCCAGTC 180

* *** * *** * ***

REV_AGM_CON_SEQ_1 ----------------------------------------GAATTGGTGAGAACAAAT-- 93

MV_589246-2022_111_HMBSrev_B10.ab1 AGGTNNANTANNNCCNNNTNNNNGCTTTTNTGGTTTGAAGGAAATTCTGGTACGGAGTGA 240

*** * ** * * *

REV_AGM_CON_SEQ_1 -----GAGATTATATGCACTCNTGTTTA--TTACCCCCTCAC-CCTCCAGCTTTGGTACC 145

MV_589246-2022_111_HMBSrev_B10.ab1 AGTCCATACTNNGCTGCGCTCCTGTGTGCTTGACCTGATGGACCATCACCCCTTTCTCCC 300

* *** *** *** * * *** * * ** * ** * **

REV_AGM_CON_SEQ_1 TGGGCAGGGACATG-----GATGGTAGCCTGCATGGTGTCTT------------------ 182

MV_589246-2022_111_HMBSrev_B10.ab1 CGGCCAAANGCGNNGAGGCGCTGTAAACCTCNATGACNCCACCTNTCAACCACCGACAAT 360

** ** * * ** * *** *** *

REV_AGM_CON_SEQ_1 -----------------------GTATGCTATCTGAGCCATCTAGACTCCAGACTCCTCC 219

MV_589246-2022_111_HMBSrev_B10.ab1 GGCTCCGTGNNGGTGCNTGTTCCTGGTCGTACCATAATCCAGCAGACTCCAGACTTCTNA 420

* ** * * * ************ **

REV_AGM_CON_SEQ_1 AGT--------------------------------------------------------- 222

MV_589246-2022_111_HMBSrev_B10.ab1 NTCANGTAAANAGATGAANACTTCTNCNACAAATNGTAAACGTNGANCNNGATNNNNANC 480

REV_AGM_CON_SEQ_1 ------------------------------------------------------------ 222

MV_589246-2022_111_HMBSrev_B10.ab1 NCCNNTTTTGACTNTCGCCNNCTATCNNGNANGGANNNANNCCNNNTTTTNTTTATCTAN 540

REV_AGM_CON_SEQ_1 ------------------------------------ 222

MV_589246-2022_111_HMBSrev_B10.ab1 ANTAGGATGGNAANGTATCANNTTANTNGGTNNAGN 576

Mosquito 112

REV_AGM_CON_SEQ_1 ------------------------------------------------------------ 0

MV_589246-2023_112_HMBSrev_B11.ab1 NNNNNNNNANNNNNNNNNTNNNNNNNNNNCNGCTTNCCNNTNNNACCNGCATCAGCGGTG 60

REV_AGM_CON_SEQ_1 ------------------------------------------------------------ 0

MV_589246-2023_112_HMBSrev_B11.ab1 TGACNCTACTTCCATACNCTTTATCAATGTGATAATGGCAGCTGTTGATTACTTTTCTCC 120

REV_AGM_CON_SEQ_1 --------------GTTACGAGCAGTGATGCCTAC------------------------- 21

MV_589246-2023_112_HMBSrev_B11.ab1 ANCAGACTCTTCTAGTACTCTGGAGTGGTGTGACTCATCTCTCAGTACCAGACTCCAGAC 180

** * **** **

REV_AGM_CON_SEQ_1 ---------CAGCTGTGGGTCATCCTCAGGGCCATCTTCATGCTGTATGCG-GGAAGGAG 71

MV_589246-2023_112_HMBSrev_B11.ab1 TCCTCCANTCAGGTAAAACTTTCAATAACGGATCTCTTGGTTCTGGCATCGATGAAGAAC 240

*** * * * * ** **** * *** ** **** *

REV_AGM_CON_SEQ_1 GTGGGAATTGGTGAGAACAAATGAG--------ATT-----ATATGCACTCNTGTTTATT 118

MV_589246-2023_112_HMBSrev_B11.ab1 GCAGCGAAATGCGATACGTAGTGTGAATTGTAGAATTCANTGAATCATCGAATCTTTGAA 300

* * * * ** * * ** * * * ** * * ***

REV_AGM_CON_SEQ_1 ACCCCC-----------------------------------------------TCACCCT 131

MV_589246-2023_112_HMBSrev_B11.ab1 CGCACATTGCGCCCTTTGGTATTCCAAAGGGCATGCCTGTTCGAGCGTCATTTGTACCCT 360

* * *****

REV_AGM_CON_SEQ_1 CCAGCTTTGGTACCTGGGCAGGGACATGGATGGTAGC----------------------- 168

MV_589246-2023_112_HMBSrev_B11.ab1 CAAGCTTTGCTTGGTGTTGGGGGTCTTTGTCTCTCACGAGACTCGTGNTANANTGATTGG 420

* ******* * ** *** * * * * *

REV_AGM_CON_SEQ_1 ----CTGCATGGTG--------------------TCTTGT--------------ATGCTA 190

MV_589246-2023_112_HMBSrev_B11.ab1 CAGCCGACCTACTGGTTTCGGAGCGCAGCACAATTCTTGCACTTTGAATCAGACTTGGTT 480

* * * ** ***** ** *

REV_AGM_CON_SEQ_1 TCTGAGCCATCTAGACTCCAGACTCCTCCAGT---------------------------- 222

MV_589246-2023_112_HMBSrev_B11.ab1 GAGCATCCATCAAGACCACATTTTTTTCNNCTTTTGACCTCGGATCAGGTAGGGATACCC 540

* ***** **** ** * ** *

REV_AGM_CON_SEQ_1 -------------------- 222

MV_589246-2023_112_HMBSrev_B11.ab1 GCTGAACTTAAGCATATNNG 560

Mosquito 113

REV_AGM_CON_SEQ_1 ------------------------------------------------------------ 0

MV_589246-2024_113_HMBSrev_B12.ab1 NNNNNNNNNNCGNNGNTNNCNGNGNGCNNNCTTNNCCNNNTANAGNANCCNNCATCAGCA 60

REV_AGM_CON_SEQ_1 ------------------------------------------------------------ 0

MV_589246-2024_113_HMBSrev_B12.ab1 GTANNGGCGTAACTTCGATACACTTTAGCGATGTGATAATGGCAGCTGTTGATTACTTTT 120

REV_AGM_CON_SEQ_1 ------------------GTTACGAGCAGTGATGCCTACCAGCTGTGGGTC--ATCCTCA 40

MV_589246-2024_113_HMBSrev_B12.ab1 CTCCAGCAGACTCTTCTAGTACTCTGGAGTGGTGTGACTCATCTCTCAGTACCAGACTCC 180

** * **** ** ** ** * ** * ***

REV_AGM_CON_SEQ_1 GG--------------------------GCCATCT------------------------- 49

MV_589246-2024_113_HMBSrev_B12.ab1 AGACTCCTCCAGTCAGGNNNNNNACNNGGGGGTNNNNNTTNTCGGTTNACGAAGAATCCC 240

* * *

REV_AGM_CON_SEQ_1 ---------------TCATGCTGTATGCGGGAAGGAGGTGGGAATTGGTGAGAACAAATG 94

MV_589246-2024_113_HMBSrev_B12.ab1 GCGCNGTGANNANGGATATCCNNTAGGCGAACGATTCGTATCAACNTAAAGATTCAAATT 300

** * ** *** ** ** *****

REV_AGM_CON_SEQ_1 AGATTATATGCACTCNTGTTTATTAC---------------------------------- 120

MV_589246-2024_113_HMBSrev_B12.ab1 AACTGATATGGATGGNTTTTTGTTGGGATTGACTCANTGNNNATATCCTGCCCGACTCCG 360

* * ***** * ** *** **

REV_AGM_CON_SEQ_1 ----------------------------CCCCTCACCCTCCAGCTTTGGTACCTGGGCAG 152

MV_589246-2024_113_HMBSrev_B12.ab1 CACTCCTCGGGGCANGTAATCTTTGNNTCTCATGAGACTCGNGCTANNATGATTGGCAGG 420

* * * * *** *** * *** *

REV_AGM_CON_SEQ_1 GGACATGGATGGTAGCCTGCATGGTGTCTTGTATGCTATCTGAGC--------------- 197

MV_589246-2024_113_HMBSrev_B12.ab1 CGANCTAGTGGTTTGGGAGTGGATNCGAATTTTTGCNNTTTGAATNAGACTTGGTGGACT 480

** * * * * * * * * *** * ***

REV_AGM_CON_SEQ_1 ---CATCTAGACTCCAGACTCCTCCAGT-------------------------------- 222

MV_589246-2024_113_HMBSrev_B12.ab1 NTCCATCAAGGNNACGTTTTACTCAAATTTTGANCGCCGATCACGNANGGATATNCGCNG 540

**** ** * * *** * *

REV_AGM_CON_SEQ_1 ------------------------------------------ 222

MV_589246-2024_113_HMBSrev_B12.ab1 AANTTAANNGTGAANTAAGGGGANANNNCGANATANNANTTT 582

Mosquito 114

REV_AGM_CON_SEQ_1 -----------------------GTTA---------CGAGCAGTGATGCCTACCA----- 23

MV_589246-2025_114_HMBSrev_C01.ab1 NNNNNNNTTCGNCGCAACTTTCNATTAAGACCAGCATCAGCAGTGTGGCGTAACTTCGAT 60

*** ******* ** ** *

REV_AGM_CON_SEQ_1 -------------------------GCTGT-GGGTCATCCTCAGGGCCATCTTCATGCTG 57

MV_589246-2025_114_HMBSrev_C01.ab1 ACACTTTAGCGATGTGATAATGGCAGCTGTTGATTACTTTTCTCCAGCAGACTCTTCTAG 120

***** * * * ** ** ** * *

REV_AGM_CON_SEQ_1 TATGCGGGAAGGAGGT----------------------------------------GGGA 77

MV_589246-2025_114_HMBSrev_C01.ab1 TACTCTGGAGTGGTGTGACTCATCTCTCAGTACCAGACTCCAGACTCCTCCAGTCAGGTA 180

** * *** * ** ** *

REV_AGM_CON_SEQ_1 ATTGGTGAGAACAAATG-----AGATTATATGCACTCNTGTTTATT--------ACCCCC 124

MV_589246-2025_114_HMBSrev_C01.ab1 AANGCNCTCAGCAAAACCGTTTNGNTGNTGNGCTCNGCTATCACTGCAGCAACTCCACCG 240

* * * **** * * * ** * * * * * **

REV_AGM_CON_SEQ_1 TCACCCTCCAG-CTTTGGTACCTGGGCAGGGACATGGAT---GGTAGCCTGCATGGTGTC 180

MV_589246-2025_114_HMBSrev_C01.ab1 AAAGACTCCGCACTCCTCTAGTCNGGTAANAACATTCAATTAGCAAATATGGATGGTTTT 300

* **** ** ** ** * **** * * * ** ***** *

REV_AGM_CON_SEQ_1 T-----------TGTATGCTATCTGAGCCATCTAGACTCCAGACTCCTCCAGT------- 222

MV_589246-2025_114_HMBSrev_C01.ab1 TAGTTGTGATTGACTCACTGTACATATCCGTCCAGACTCCANACTCCTCCATTCAGGTAA 360

* * * * ** ** ******** ********* *

REV_AGM_CON_SEQ_1 --- 222

MV_589246-2025_114_HMBSrev_C01.ab1 ANN 36

Mosquito 115

REV_AGM_CON_SEQ_1 -------------GTTACGAGCAGTGATGCCTACCAGCTG--TGGGTCATCCTCAGGGCC 45

MV_589246-2026_115_HMBSrev_C02.ab1 NNNNNNNNNNNNNNNNNNNNNNNCTGCNNACTCCCNACTCCTCCNGTCAGGTAAGACGCN 60

** ** ** ** **** **

REV_AGM_CON_SEQ_1 ATCTTCATGCTGTATGCGGGAAG----------GAGGTGGGAATTGGTGAGAACAAATG- 94

MV_589246-2026_115_HMBSrev_C02.ab1 TTTTTGATCACGTNACCGATCTGGCGCNGGATGCTGTTGGTNGTTTTCCCGAGCTACTCT 120

* ** ** ** ** * * *** ** ** * * *

REV_AGM_CON_SEQ_1 -----------------------AGATTATATGCACTCNTGTTTATTACCCCCTCACCCT 131

MV_589246-2026_115_HMBSrev_C02.ab1 ACTANAATTCTGAAGTCGGGTAATCAATATANNAATGCCCGTTNCCCGAAACAAAACCCT 180

* **** * * *** * *****

REV_AGM_CON_SEQ_1 CCAG---------CTTTGGTAC---C----------------------------TGGGCA 151

MV_589246-2026_115_HMBSrev_C02.ab1 GCCGGCNACTCCGCAGTCGTCCATGCTGGTAANCNGACTCCANACTCCTCCANTCAGGTA 240

* * * * ** * * ** *

REV_AGM_CON_SEQ_1 GGGACATGGATGGTAGCCTGCATGGTG--------------------------------- 178

MV_589246-2026_115_HMBSrev_C02.ab1 ANGGTATGCCTAAACGCCNGAATCCTNCCNNTTGGGGAAANNNAATGAGAGCCAAATCGN 300

* *** * *** * ** *

REV_AGM_CON_SEQ_1 -------------------TCTTGTATGCTATCT------------------GAGCCATC 201

MV_589246-2026_115_HMBSrev_C02.ab1 GATTCNAATCTGTTTGGTTACTGNANTGNNACCNTGCGTGGATNCGNNGGNGCCGATTCC 360

** ** * * * *

REV_AGM_CON_SEQ_1 TAGACTCCAGACTCCTCCAGT----------- 222

MV_589246-2026_115_HMBSrev_C02.ab1 TTGACTNGNGACTCCTCNGGTCAGGTAACGGN 392

* **** ******** **

Mosquito 116

REV_AGM_CON_SEQ_1 -------------------------GTTACGAGCAGTGATGCCTACCAGCTGTGGGTCAT 35

MV_589246-2027_116_HMBSrev_C03.ab1 NNNNNNNNNNNNNNGGGNNNGNGNNCTTTNTNTTNNGANNNNNTCCGTGGTGTGGCGTAT 60

** * * * ***** **

REV_AGM_CON_SEQ_1 CCTCAGGGCCATCTTCATGCTGTA-TGCGGGAAGGAGGTGGGAATTGGTGAGAACAAATG 94

MV_589246-2027_116_HMBSrev_C03.ab1 CTTCGATACACTTT-GGCGATGTGGTAATGGATGCTGTTGATTAGTTTTCTCCAGNAGAG 119

* ** * * * * *** * *** * * ** * * * * * *

REV_AGM_CON_SEQ_1 A-----GATTATATGCACTCNTGTTTATTACCC-----CCTCACCCTCCAGCTTTGGTAC 144

MV_589246-2027_116_HMBSrev_C03.ab1 TCTTCTAGTACTCTGGAGTGGTGTGACTCATCTCTCAGTGCCAGACTCCAGACTCC---T 176

* * ** * * *** * * * ** ****** *

REV_AGM_CON_SEQ_1 CTGGGCAGGGACATGGATGGTAGCCTGCATGGTGTCTTGTATGCTATCTGAGCCATCTAG 204

MV_589246-2027_116_HMBSrev_C03.ab1 CCAGTCAGGTAANGGCGTGCTGGTGTCG------------------ATGCTGGCGGTCAG 218

* * **** * * ** * * * * * **

REV_AGM_CON_SEQ_1 ACTCCAGACTCCTCCAGT--------- 222

MV_589246-2027_116_HMBSrev_C03.ab1 ACTCCAGACTCCTCCAGTCAGGTAANG 245

******************

Mosquito 117

REV_AGM_CON_SEQ_1 ------------------------------------------------------------ 0

MV_589246-2028_117_HMBSrev_C04.ab1 NNNNNNNNNCGNAGNATNNNNTAACTGCATNACTCCANACTCCTCCAGTCAGGTAAGACT 60

REV_AGM_CON_SEQ_1 --------------GTTACGA--------------------------------------- 7

MV_589246-2028_117_HMBSrev_C04.ab1 GTATCTTCCATACNCTTTAGCGATGTGATAATGGCAGCTGTTGATTACTTTTCTCCAGCA 120

** *

REV_AGM_CON_SEQ_1 -----------------GCAGTGATGCCTACCAGCTGTGGGT--CATCCTCAGGGCCATC 48

MV_589246-2028_117_HMBSrev_C04.ab1 GACTCTTCTANTCCTCTGGAGTGGTGTGACTCATCTCTCAGTACCAGACTCCAGACTCCT 180

* **** ** ** ** * ** ** *** * *

REV_AGM_CON_SEQ_1 TTCATGCTGTATGCGGGAAGGAGGTGGGAATTGGTGAGAACAAATGAGATTATATGCACT 108

MV_589246-2028_117_HMBSrev_C04.ab1 CCAGTCAGGTAANCTCTGTTCAGGTAAGGTTTNTCGGNTCAGGNAANNNTGAAAAACGCT 240

* *** * **** * ** * * * * * **

REV_AGM_CON_SEQ_1 CNTGTTTATTACCCCCTCACCCTCCAGCTTTGGTACCTGGGCAGGGACATGGATGGTAGC 168

MV_589246-2028_117_HMBSrev_C04.ab1 NGGGNTGTATATCNGTTNNTGNTTCCACTAATATCGANGTANCGATTAATATTTNAAGAT 300

* * ** * * * * ** * * * ** *

REV_AGM_CON_SEQ_1 CTGCATGGTGTCTTGTATGCT----------ATCTGAGCCATCTAGACTCCAGACTCCTC 218

MV_589246-2028_117_HMBSrev_C04.ab1 ATGGAGCGTTTNTTTTTCTGATCCNNGNCTGTACAGATCCGGCGTTACTCTAAACTCTTC 360

** * ** * ** * * ** ** * **** * **** **

REV_AGM_CON_SEQ_1 CAGT------------------------------ 222

MV_589246-2028_117_HMBSrev_C04.ab1 CACACTCGNAAAAGTTGGGNGACCCTGANTCTCA 394

**

Mosquito 118

REV_AGM_CON_SEQ_1 ------------------------GTTACGAGCAGTGATGCCTACCAGCTGTGGGTCATC 36

MV_589246-2029_118_HMBSrev_C05.ab1 NNNNNNNNNNNNNNNNANNNGNNCTTTCCNTTAAGACCAGCATCAGCAGTGTGGCGTAAC 60

** * ** ** * ***** * *

REV_AGM_CON_SEQ_1 CTCAGGGCCATCTTCATGCTGTATGCGGGAAGGAGGTGGGAATTGGTGAGAACAAATGAG 96

MV_589246-2029_118_HMBSrev_C05.ab1 TTCGATACACTTTAGCGATGTGATAATGGCAGCTGTTGATTACTTTTCTCCAGCA--GAC 118

** * * * ** ** ** * ** * * * * * **

REV_AGM_CON_SEQ_1 ATTATATGCACTCNTGTTTATTACCCCCTCACCCTCCAGCTTTGGTACCTGGGCAGGGAC 156

MV_589246-2029_118_HMBSrev_C05.ab1 TCTTCTAGTACTCTGG-------AGTGGTGTGACTCATCTCTCAGTACCAGACTCCAGAC 171

* * **** * * *** * ***** * ***

REV_AGM_CON_SEQ_1 ATGGATGGTAGCCTGCATGGTGTCTTGTATGCTATCTGAGCCATCTAGACTCCAGACTCC 216

MV_589246-2029_118_HMBSrev_C05.ab1 TCCTCCAGTCAGGTAAANGGNCTCTTGNATGCNGTCTGANCCNGCTGGACTCCNGACTCC 231

** * * ** ***** **** ***** ** ** ****** ******

REV_AGM_CON_SEQ_1 TCCAGT----------------------------- 222

MV_589246-2029_118_HMBSrev_C05.ab1 TCCCGNTCNGGTAAGCGNGCNNGCATTGAGTANTN 266

*** *

Mosquito 119

REV_AGM_CON_SEQ_1 -------------------------------------------------GTTACGAGCAG 11

MV_589246-2030_119_HMBSrev_C06.ab1 NNNNNNNNNNNCNNNTNNNNNNNNNAGCNCTCTGGCAGCTTTTGCCCACCTTTGGGGTAC 60

** * * *

REV_AGM_CON_SEQ_1 TGATGCCTACCAGCTGTGGGTCATCCTCAGGGCCAT---------------------CTT 50

MV_589246-2030_119_HMBSrev_C06.ab1 GTGCCCTCCCCAGATCTTACACAGCACCTGGGGGAGCAGGATGGGCTTGGGGGGGTGGCT 120

* **** * * ** * * *** * *

REV_AGM_CON_SEQ_1 CATGCTGTATGCGGGAAGGAGGTGGGAATTGGTGAGAACAAATG-------AGATT---- 99

MV_589246-2030_119_HMBSrev_C06.ab1 CATGTCCTGTGATGGAGCACGCTCAGCACGGGGCTCAGCTCAGGGGCAGACAGACTCCAG 180

**** * ** *** * * * * ** * * * * *** *

REV_AGM_CON_SEQ_1 --ATATGCACTCNTGTTTA-------TTAC------------CCCCTCACCCTCCAGCTT 138

MV_589246-2030_119_HMBSrev_C06.ab1 ACTCCTCCAGTCAGGTAAAACATAGTTGACCCGANAAGTGCTCTACTTTCNCTGCTGCTT 240

* ** ** ** * * ** * ** * ** * ****

REV_AGM_CON_SEQ_1 TGGTACCTGGGCAGGGACATGGATGGTAGCCTG---CATGGTGTCT-------TGTATGC 188

MV_589246-2030_119_HMBSrev_C06.ab1 G--TTCNANCAATAGGAGATAGCTGCACCATTACTGACAACTCTATTTGAGAGCCGTTGT 298

* * *** ** * ** * * * * **

REV_AGM_CON_SEQ_1 TATCTGAGCCATCTAGACTCCAGACTCCTCCAGT------- 222

MV_589246-2030_119_HMBSrev_C06.ab1 CTGAACCAGTATCAAGACTCCAGACTCCTCCAGTCAGGTAA 339

*** ********************

Mosquito 121

REV_AGM_CON_SEQ_1 -------GTTACGAGCAGTGATGCCTACCAGCTGTG-GGTCATCC---TCAGGGCCATCT 49

MV_589246-2032_121_HMBSrev_C08.ab1 NNNNNNNNNNCGNANNNNNNATNNCTGCANACTCCAGACTCCTCCAGTCAGGTAANACGT 60

* ** ** * ** ** *** * * *

REV_AGM_CON_SEQ_1 TCATGCTGTATGCGGGAAGGAGGTGGGAATTGG------TGAGAA--------------- 88

MV_589246-2032_121_HMBSrev_C08.ab1 ATCTTCCATACACTTTANCGATGTGATAATGGCAGCTGTTGATTACTTTTCTCCNGCAGA 120

* * ** * * ** *** *** * *** *

REV_AGM_CON_SEQ_1 CAAATGAGATTATATGCACTCNTGTTTATTACCC-----CCTCACCCTCCAGCTTTGGTA 143

MV_589246-2032_121_HMBSrev_C08.ab1 CTCTTCTAGTACTCTGGAGTGGTGTGACTCATCTCTCAGTACCAGACTCCAGACTC---C 177

* * * * ** * * *** * * * ** ****** *

REV_AGM_CON_SEQ_1 CCTGGGCAGGGACATGGATGGTAGCCTGCATGGTGTCTTGTATGCTATCTGAGCCATCTA 203

MV_589246-2032_121_HMBSrev_C08.ab1 TCCAGTCAGGTAANGGNCNGCTGGTGTCGATGCTGG------------------CTGGCC 219

* * **** * * * * * * *** ** *

REV_AGM_CON_SEQ_1 GACTCCAGACTCCTCCAGT-------------------------------- 222

MV_589246-2032_121_HMBSrev_C08.ab1 CACTCCAGACTCCTCCAGTCAAGTAANNGTTCGNGNGTCGAGTTGCCCCTT 270

******************

Mosquito 122

REV_AGM_CON_SEQ_1 ----------------GTTACGAGCAGTGATGCCTACCAGCTGT------GGGTCATCCT 38

MV_589246-2033_122_HMBSrev_C09.ab1 NNNNNNNNNNNNNNNNNNNNNNNNNNCNNNNNCTNAACCGCTGGACATTGGCCTCNTCCT 60

* * * **** * ** ****

REV_AGM_CON_SEQ_1 CA---------GGGCCATCTTCATGCTGTATGCGGGAAGGAG------------------ 71

MV_589246-2033_122_HMBSrev_C09.ab1 GACTCCTCCCGGGGGTAAACTGGTGCGGGATGCGCCTTAATTGTGCAAACCACGCTCTNC 120

* *** * * *** * *****

REV_AGM_CON_SEQ_1 ------------------------------------------------------------ 71

MV_589246-2033_122_HMBSrev_C09.ab1 ACTCCNANTCCTCCAGTCGGGTAACTCATCTCTCAGTACCNGACTCCAGACTCCTCCAGT 180

REV_AGM_CON_SEQ_1 -------------------------------------------------GTGGGAATTGG 82

MV_589246-2033_122_HMBSrev_C09.ab1 CANGTAANNTCANNTGGGGGCCCTCCGGTTCTGGCTTCTCCTNNTTCNTTTTTGAACGTG 240

* *** *

REV_AGM_CON_SEQ_1 TGAGAACAAATGAGATTATATGC--------ACTCNTGTTTATTACCC---CCTCACCCT 131

MV_589246-2033_122_HMBSrev_C09.ab1 NGCGAACTAATGTAAATTGNTGNNTNNNCCNNNTTCNTTTNNTNAACGCNCNTTGCGCCC 300

* **** **** * * ** * ** * * * * **

REV_AGM_CON_SEQ_1 CCAGCTTTGGTACCTGGGCAGGGACATGGATGGTAGCCTGCATGGTGTCTT--GTATGCT 189

MV_589246-2033_122_HMBSrev_C09.ab1 TTTGNTATTCCAAATGGNATGCGGGTTCCGGCGCCNCTTGCACCCTCAGCTTTGNNNGGN 360

* * * * *** * * * * * **** * * * *

REV_AGM_CON_SEQ_1 ATCTGAGCCATCTAGACTCCAGACTCCTCCAGT--------------------------- 222

MV_589246-2033_122_HMBSrev_C09.ab1 GTTGGGCGCNNGGNGCCNCCNCCCTTTNCNNGTNNATNATTGGCANCAGACCATCTGGTT 420

* * * * * ** ** * **

REV_AGM_CON_SEQ_1 ------------------------------------------------------------ 222

MV_589246-2033_122_HMBSrev_C09.ab1 TCGGANCGTAGTACTTTTCTCGNCATTTGANCCGCCCTTGGTCTACCACCNATCATGACC 480

REV_AGM_CON_SEQ_1 ------------------------------------------------------------ 222

MV_589246-2033_122_HMBSrev_C09.ab1 ACNTTTTTTTCANCNTCCNNACTCCTATCAGGNAGTGANNCNCNCTGANNTNNNNNTATT 540

REV_AGM_CON_SEQ_1 ---------------------- 222

MV_589246-2033_122_HMBSrev_C09.ab1 AATAATNNGANNNNAAGAAACA 562
